# Nine Unique Iridoids and Iridoid Glycosides From *Patrinia scabiosaefolia*

**DOI:** 10.3389/fchem.2021.657028

**Published:** 2021-03-29

**Authors:** Zhenhua Liu, Yun Niu, Li Zhou, Lijun Meng, Sitan Chen, Mengke Wang, Jiangmiao Hu, Wenyi Kang

**Affiliations:** ^1^National Center for Research and Development of Edible Fungus Processing Technology, Henan University, Kaifeng, China; ^2^State Key Laboratory of Phytochemistry and Plant Resources in West China, Kunming Institute of Botany, Chinese Academy of Sciences, Kunming, China; ^3^College of Life Sciences, University of Chinese Academy of Sciences, Beijing, China; ^4^Joint International Research Laboratory of Food & Medicine Resource Function, Henan University, Kaifeng, China

**Keywords:** *Patrinia scabiosaefolia*, iridoid, iridoid glycoside, bis-iridoid glycoside, cytotoxic activity

## Abstract

*Patrinia scabiosaefolia* is a medical and edible Chinese herb with high nutritional and medicinal value. The continuing study of its chemical constituents led to the discovery of nine unique iridoids and iridoid glycosides, including three new iridoids (**1**-**3**) and six previously unknown irioid glycosides (**5**-**10**), and one known compound (**4**). Among them, compound **1** was a deformed iridoid, while compounds **3**, **5**-**7**, and **10** formed a new ring in their skeletons which was uncommon in this genus. For compound **3**, the new ring existed between C-3 and C-10, while a 1,3-dioxane appeared between C-7 and C-10 in compounds **5**-**7** and **10**. Moreover, compound **10** was a bis-iridoid glycoside, which was the first reported in *P*. *scabiosaefolia*. And the sugar of irioid glycosides (**5**-**10**) was glucose at C-11, except in **9** which had a 5-deoxyglucose moiety. All their structures were confirmed based on the extensive spectroscopic analysis, including IR, UV, HR-ESI-MS, ECD, and 1D- and 2D-NMR experiments. Their cytotoxic activities against HL-60, A-549, SMMC-7721, MCF-7, SW480 were also tested.

**Graphical Abstract:**
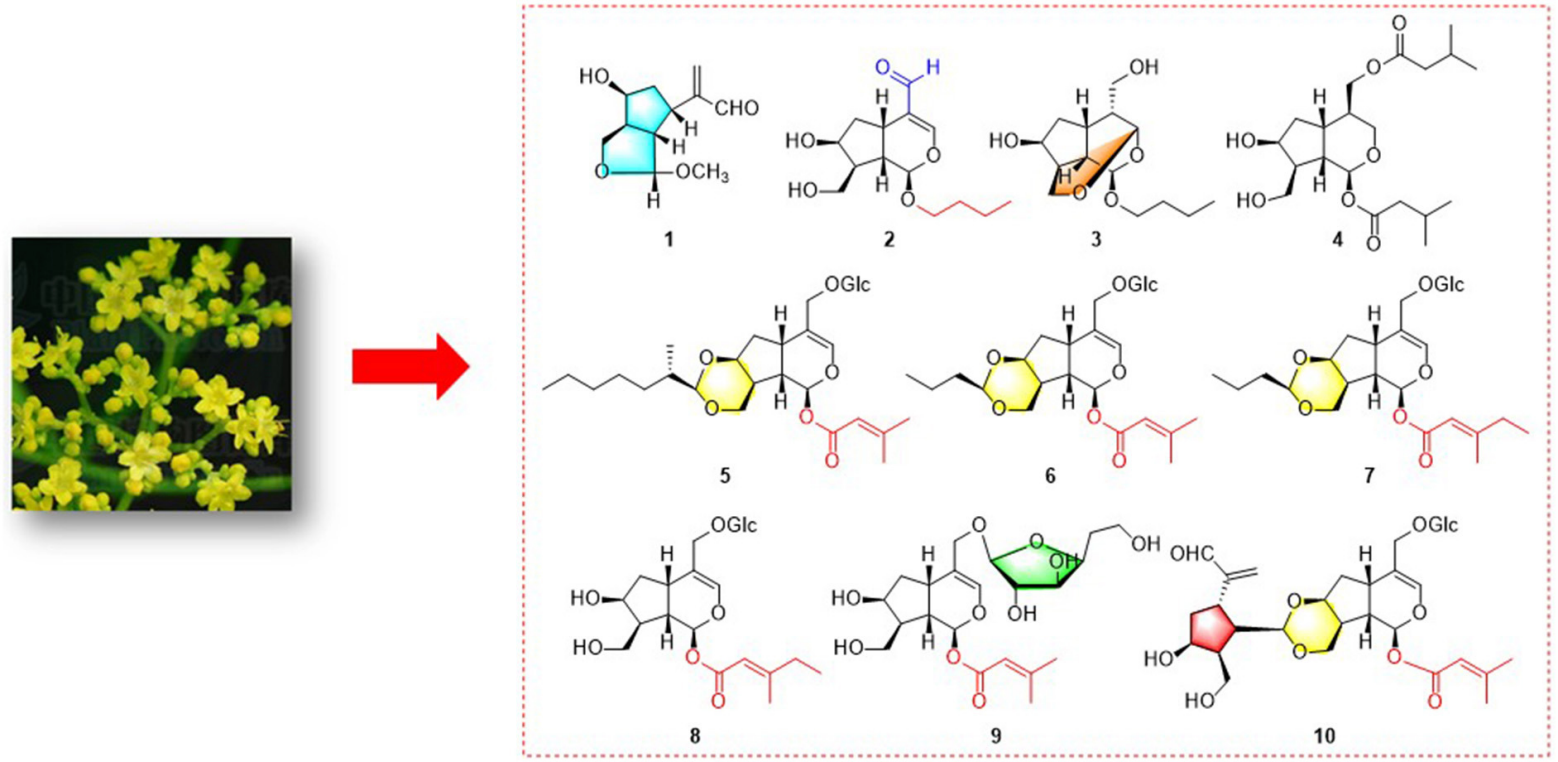
Nine unique iridoids and iridoid glycosides from *Patrinia scabiosaefolia*.

## Introduction

There are about 400 species in 13 genera of Valerianaceae, mostly distributed in northern temperate zones and the subtropics or frigid zones. In China, this family comprises three genera, *Nardostachys, Valeriana*, and *Patrinia*, which can be found all over the country (Delectis Flora Reipublicae Popularis Sinicae Agendae Academiae Sinicae Edita., 1986). Plants from the genus *Patrinia*, such as *P. scabiosaefolia, P. villosa*, and *P. heterophylla*, have a long history of use as a traditional Chinese medicine for detoxification, swelling, empyema, liver protection, and cholagogue,. Some species are also edible as wild herbs, for example *P. scabiosaefolia, P. villosa, P. punctiflora*, and *P. angustifolia* (Xiao et al., 2007).

Among them, *P. scabiosaefolia* is a medical and edible Chinese herb, first recorded in “Sheng Nong's Herbal Classic.” It is of great nutritional value and is enriched with amino acids, vitamins, β-carotene, and trace elements. The content of vitamin C per 100 grams is 42.65 mg, which is higher than that in some vegetables and fruits. Also, there is 14995.69 mg of amino acids in it, including eight kinds of essential amino acids which account for 36.06% of the total amount of amino acids (Zhong et al., 2001). It was also verified to have effects on the initial stages of edema, appendicitis, endometriosis, and inflammation (Delectis Flora Reipublicae Popularis Sinicae Agendae Academiae Sinicae Edita., 1986).

Phytochemical research showed that different kinds of compounds existed in the *Patrinia* genus, including triterpenes, iridoids, flavones, lignans, and their glycosides (Kim and Kang, 2013). Among these, iridoids are considered as the main components with diverse structures and various activities, which attract our attention to further explore the plant *P. scabiosaefolia*. As a result, we found a series of iridoids from ethyl acetate extract of *P. scabiosaefolia*, including three bis-iridoids which were first reported (Liu et al., 2017a,b; Liu et al., 2019).

**Figure 1:**
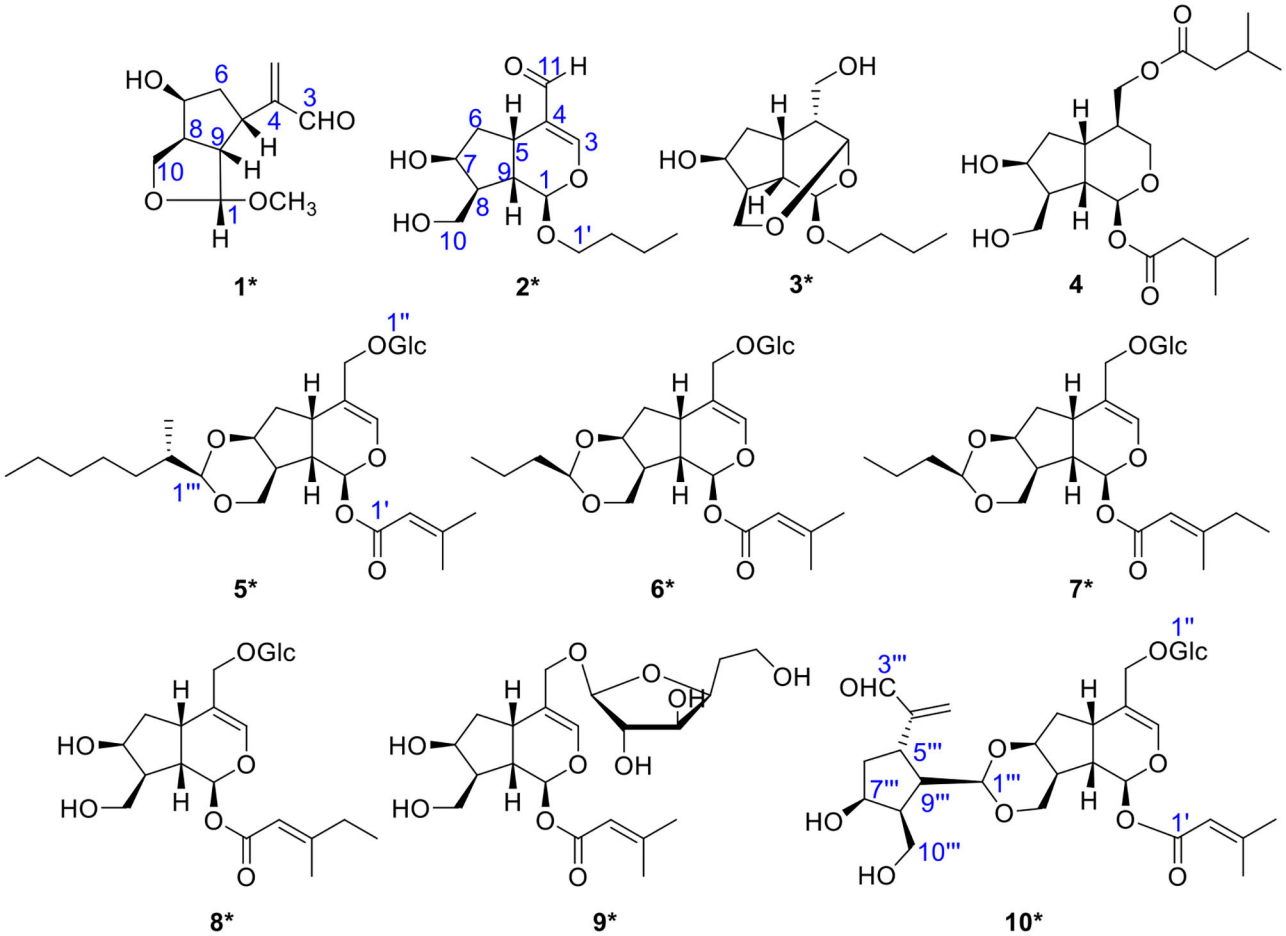
The chemical structures of compounds **1**-**10** (*new compounds).

In the continuing study, chemical constituents on *n*-butanol extract of *P*. *scabiosaefolia* led to the discovery of nine unique iridoids and iridoid glycosides, including three new iridoids (**1**-**3**) and six novel irioid glycosides (**5**-**10**), and one known compound (**4**) (Figure 1, Graphical Abstract). Among them, compound **1** was a deformed iridoid, compound **3** formed a cycle between C-3 and C-10, compounds **5**-**7** with a 1,3-dioxane between C-7 and C-10, and compound **10** was a bis-iridoid glycoside, which was the first reported in *P*. *scabiosaefolia*. Herein, we discussed their isolation, structure elucidation, and their cytotoxic activities.

## Experimental Section

### General Experimental Procedures

Optical rotation was obtained on a JASCO P-1020 digital polarimeter (Horiba, Tokyo, Japan). UV spectra were measured by a Shimadzu UV-2401 PC spectrophotometer (Shimadzu, Kyoto, Japan). IR spectra were obtained on a Bruker Tensor 27 infrared spectrophotometer (Bruker Optics GmbH, Ettlingen, Germany) with KBr pellets. Mass spectra were performed on an API QSTAR time-of-flight spectrometer (MDS Sciqaszex, Concord, Ontario, Canada) and LC/MS-IT-TOF (Shimadzu, Kyoto, Japan) spectrometer. NMR spectra were recorded on Bruker DRX-500 and Av III-800 instruments with TMS as the internal standard (Bruker, Bremerhaven, Germany). The chemical shifts were given in δ (ppm) with reference to the solvent signal. Column chromatography was performed on silica gel (200–300 and 300–400 mesh, Qingdao Marine Chemical Inc., Qingdao, China), Lichroprep Rp-18 gel (40–63 μm, Merck, Darmstadt, Germany), MCI gel CHP-20P (75–150 μm, Mitsubishi Chemical Corp., Tokyo, Japan), Sephadex LH-20 (20–150 μm, Amersham Biosciences, Uppsala, Sweden), and YMC^*^GEL ODS-A-HG (50 μm, YMC Co. Ltd. Japan). Fractions were monitored by TLC, and spots were visualized by UV light and sprayed with 10% H_2_SO_4_ in EtOH, followed by heating.

### Plant Material

The whole plants of *Patrinia scabiosaefolia* were collected in October 2010 from Shucheng county, Anhui Province, People's Republic of China, and were stored in a cool and dry place at room temperature. The material was identified by Prof. Shou-Jin Liu in Anhui University of Chinese Medicine and a voucher specimen (Wan1295) was deposited in Anhui University of Chinese Medicine. The plants of *P*. *scabiosaefolia* are common in the local area and the collection was permitted. We also ensured that the local population of *P*. *scabiosaefolia* was not destroyed through the means of collection at different locations.

### Extraction and Isolation

The air-dried and powdered whole plants (29 kg) of *P*. *scabiosaefolia* were extracted with 95% ethanol (3 × 75 L) under room temperature and concentrated under reduced pressure. Then the residue (3 kg) was dissolved in water and partitioned successively with *n*-butanol to yield *n*-butanol extract (0.85 kg) after concentration. The *n*-butanol extract was subjected to silica gel column chromatography eluted with a gradient of CHCl_3_-MeOH (8:1→ 0:1, v/v) to obtain five fractions 1-5 by TLC plate analysis.

Fraction 2 (22 g) was separated by silica gel column chromatography eluted with a gradient system of CHCl_3_-MeOH (6:1→ 1:1, v/v) to afford 4 subfractions (Fr.2-1 to Fr.2-4). Fr.2-1 (5.9 g) was performed by Rp-18 column chromatography (MeOH-H_2_O, 50:50→ 100:0, v/v) to afford 6 subfractions (Fr.2-1-1 to Fr.2-1-6). Fraction 2-1-2 (1.0 g) was applied to Sephadex LH-20 column chromatography (MeOH-H_2_O, 90:10, v/v) and then purified by silica gel column chromatography repeatedly to obtain **1** (1.0 mg). Fraction 2-2-3 (1.3 g) was submitted to silica gel column chromatography eluted with a gradient of EtOAc-MeOH (4:1→ 1:1, v/v) to afford A (180 mg) and B (240 mg), and then purified by semi-prep. HPLC (MeOH-H_2_O, 65:35, v/v) to obtain **3** (7.0 mg) and **4** (16.0 mg) respectively. Fraction 2-2-4 (2.1 g) was separated by the same methods as Fraction 2-2-3 and purified by semi-prep. HPLC (MeOH-H_2_O, 55:45, v/v) to afford **5** (1.0 mg) and **10** (16.0 mg).

Fraction 3 (15 g) was subjected to silica gel column chromatography eluted with a gradient system of CHCl_3_-MeOH (5:1→ 1:1, v/v) to afford 6 subfractions (Fr.3-1 to Fr.3-6). Fr.3-3 (4.3 g) was separated by Rp-18 column chromatography (MeOH-H_2_O, 50:50→ 100:0, v/v) to get 5 subfractions (Fr.3-3-1 to Fr.3-3-5). Fraction 3-3-3 (560 mg) was applied to Sephadex LH-20 column chromatography (MeOH-H_2_O, 90:10, v/v), and silica gel column chromatography eluted with CHCl_3_- MeOH (4:1, v/v) to obtain **2** (3.0 mg).

Fraction 4 (28 g) was submitted to Rp-18 column chromatography (MeOH-H_2_O, 30:70→ 100:0, v/v) to obtain 6 subfractions (Fr.4-1 to Fr.4-6). Fr.4-2 (3.4 g) was applied to silica gel column chromatography eluted with a gradient system of EtOAc-MeOH (4:1→ 1:1, v/v), then isolated by Sephadex LH-20 column chromatography (MeOH-H_2_O, 90:10, v/v), and further purified by semi-prep. HPLC (MeOH-H_2_O, 55:45, v/v) to afford **6** (3.0 mg) and **7** (10 mg). Fr.4-4 (5.6 g) was repeatedly separated by silica gel column chromatography and Sephadex LH-20 column chromatography (MeOH-H_2_O, 90:10, v/v), and finally purified by semi-prep. HPLC (MeOH-H_2_O, 55:45, v/v) to afford **8** (3.0 mg) and **9** (16.0 mg).

*Patrirscabioin M (**1**)*: colorless oil; [α]^23^_D_-32.2 (*c*0.03, MeOH); UV (MeOH) λ_max_ (log ε): 204 (3.42) nm; IR (KBr) ν_max_ 3,428, 2,925, 2,855, 1,632, 1,384, 1,101 cm^−1^; ^1^H and ^13^C NMR data, see Table 1; positive ESIMS *m/z* 235 [M + Na]^+^; HREIMS *m/z* 235.0943 [M + Na]^+^ (calcd for C_11_H_16_O_4_Na, 235.0941).

**Table 1:** The ^1^H and ^13^C NMR data of **1-4** (CD_3_OD, δ in ppm, *J* in Hz).

**Position**	**1**		**2**		**3**	
	**δ_H_**	**δ_C_**	**δ_H_**	**δ_C_**	**δ_H_**	**δ_C_**
1	4.89 (s)	111.2 d	4.92 (d, 5.7)	104.1 d	5.19 (d, 2.8)	99.5 d
3	9.55 (s)	196.3 d	7.40 (br s)	164.1 d	5.49 (d, 1.7)	94.7 d
4	-	154.1 s	-	125.6 s	2.00 (m)	34.5 d
5	2.91 (q, 7.5)	40.2 d	3.06 (q, 8.3)	31.2 d	3.51 (m)	29.5 d
6a	1.85 (m)	40.8 t	2.24 (m)	41.9 t	1.97 (m)	36.6 t
6b	1.78 (m)		1.55 (m)		2.02 (m)	
7	4.26 (q, 4.8)	73.3 d	4.27 (dt, 2.2, 5.1)	73.5 d	4.77 (t, 3.0)	74.4 d
8	2.86 (m)	48.6 d	1.93 (m)	49.4 d	2.57 (m)	50.2 d
9	2.47 (dd, 7.2, 8.8)	57.1 d	2.08 (m)	42.7 d	2.90 (m)	46.1 d
10a	4.19 (dd, 1.6, 8.8)	66.6 t	3.78 (dd, 12.7, 5.6)	62.3 t	4.37 (dd, 6.7, 10.5)	62.4 t
10b	3.75 (t, 8.0)		3.65 (m)		4.21 (m)	
11a	6.40 (d, 1.6)	134.5 t	9.21 (s)	193.3 d	3.79 (d, 9.6)	61.8 t
11b	6.14 (s)				4.21 (m)	
OMe	3.24 (s)	54.5 q				
R_1_-1′a			3.89 (m)	70.7 t	3.91 (m)	67.5 t
1′b			3.61 (m)		3.48 (m)	
2′a			1.57 (m)	32.7 t	1.53 (m)	32.2 t
2′b			1.39 (m)			
3′			1.40 (m)	20.3 t	1.31 (m)	19.7 t
4′			0.93 (t, 7.4)	14.1 q	0.81 (t, 7.4)	14.0 q

*Patrirscabioin N (**2**)*: light yellow oil; [α]24 D-42.6 (*c*0.05, MeOH); UV (MeOH) λ_max_ (log ε): 248 (3.72) nm; 203 (3.69) nm; IR (KBr) ν_max_ 3,425, 2,927, 2,874, 1,727, 1,625, 1,384, 1,161, 1,088 cm^−1^; ^1^H and ^13^C NMR data, see Table 1; positive ESIMS m/z 293 [M + Na]^+^; HREIMS m/z 293.1354 [M + Na]+ (calcd for C_14_H_22_O_5_Na, 293.1359).

*Patrirscabioin O (**3**)*: light yellow oil; [α]^23^_D_-11.3 (*c*0.05, MeOH); UV (MeOH) λ_max_ (log ε): 203 (3.03) nm; IR (KBr) ν_max_ 3,444, 2,926, 2,870, 1,725, 1,634, 1,383, 1,088 cm^−1^; ^1^H and ^13^C NMR data, see Table 1; positive ESIMS m/z 295 [M + Na]^+^; HREIMS m/z 295.1515 [M + Na]+ (calcd for C_14_H_24_O_5_Na, 295.1516).

*Patrinoside B (**5**)*: light yellow oil; [α]^23^_D_-54.6 (*c*0.02, MeOH); UV (MeOH) λ_max_ (log ε): 218 (4.15) nm; IR (KBr) ν_max_ 3,426, 2,929, 2,873, 1,727, 1,642, 1,076 cm^−1^; ^1^H and ^13^C NMR data, see Table 2; positive ESIMS *m/z* 593 [M + Na]^+^; HREIMS *m/z* 593.2931 [M + Na]^+^ (calcd for C_29_H_46_O_11_Na, 593.2932).

**Table 2:** The ^1^H and ^13^C NMR data of **5-9** (CD_3_OD, δ in ppm, *J* in Hz).

**Position**	**5**		**6**		**7**		**8**		**9**	
	**δ_H_**	**δ_C_**	**δ_H_**	**δ_C_**	**δ_H_**	**δ_C_**	**δ_H_**	**δ_C_**	**δ_H_**	**δ_C_**
1	5.90 (d, 5.0)	93.0 d	5.88 (d, 5.2)	93.1 d	5.90 (d, 5.0)	93.1 d	6.60 (d, 5.1)	92.6 d	5.91(d, 5.5)	93.2 d
3	6.35 (s)	139.8 d	6.30 (s)	139.9 d	6.36 (s)	139.8 d	6.62 (s)	139.0 d	6.36 (s)	140.1 d
4	-	117.3 s	-	117.2 s		117.2 s	-	116.1 s	-	116.5 s
5	3.10 (q, 8.6)	34.8 d	3.11 (q, 8.6)	34.8 d	3.12 (q, 8.5)	34.7 d	3.45 (q, 7.9)	33.5 d	3.02 (m)	34.1 d
6a	2.18 (m)	39.3 t	2.17 (m)	39.3 t	2.18 (m)	39.2 t	2.42 (m)	41.0 t	2.06 (m)	40.9 t
6b	1.76 (m)		1.74 (m)		1.76 (m)		2.04 (m)		1.82 (m)	
7	4.15 (t, 3.6)	79.3 d	4.18 (t, 3.8)	79.2 d	4.18 (t, 3.8)	79.2 d	4.74 (m)	72.7 d	4.32 (m)	73.3 d
8	2.56 (m)	41.6 d	2.54 (m)	41.6 d	2.56 (m)	41.6 d	2.36 (m)	48.7 d	1.95 (m)	49.2 d
9	1.66 (m)	42.4 d	1.66 (d, 9.2)	42.3 d	1.68 (m)	42.3 d	2.74 (m)	42.2 d	2.18 (m)	42.7 d
10a	4.04 (d, 11.9)	67.1 t	4.02 (d, 3.7)	67.1 t	4.02 (m)	67.1 t	4.35 (m)	62.1 t	3.80 (dd, 7.6, 9.6)	62.2 t
10b	3.98 (dd,12.1, 2.0)		4.26 (d, 12.0)		4.27 (d, 11.6)		4.27 (m)		3.73 (dd,5.6, 11.0)	
11a	4.26 (d, 11.2)	69.7 t	4.09 (d, 11.6)	69.7 t	4.09 (d, 11.5)	69.8 t	4.53 (d, 11.9)	69.2 t	4.06 (d, 11.4)	69.7 t
11b	4.10 (d, 11.7)						4.28 (m)		4.24 (d, 11.7)	
R_1_-1'	-	166.4 s		166.4 s		166.6 s		165.5 s		166.4 s
2'	5.71 (t, 1.2)	116.1 d	5.71 (t, 1.2)	116.1 d	5.68 (br s)	114.6 d	5.73 (q, 1.2)	114.5 d	5.71 (t, 1.4)	116.2 d
3'	-	161.2 s		161.2 s		166.2 s	-	164.2 s		161.0 s
4'	1.94 (s)	27.6 q	1.93 (d, 1.0)	27.6 q	2.22 (q, 4.4)	34.8 t	1.92 (dq, 1.0, 7.5)	33.8 t	1.93 (d, 1.2)	27.6 q
5'	2.17 (s)	20.6 q	2.17 (d, 1.0)	20.6 q	1.09 (t, 7.4)	12.3 q	0.80 (t, 7.4)	11.9 q	2.17 (d, 1.2)	20.6 q
3'-Me					2.17 (s)	19.2 q	2.14 (d, 1.2)	19.0 q		
R_11_-1”	4.29 (d, 7.8)	103.2 d	4.27 (d, 7.9)	103.2 d	4.29 (d, 7.8)	103.3 d	4.88 (d, 7.8)	104.2 d	4.22(d, 7.8)	103.8 d
2”	3.19 (dd, 9.1, 7.9)	75.1 d	3.18 (m)	75.1 d	3.19 (t, 7.9)	75.1 d	4.03 (m)	75.3 d	3.09 (dd, 7.8, 9.0)	76.9 d
3”	3.26 (m)	78.0 d	3.26 (m)	77.9 d	3.26 (m)	78.0 d	4.24 (m)	78.6 d	3.59 (m)	72.2 d
4”	3.26 (m)	71.7 d	3.26 (m)	71.7 d	3.27 (m)	71.7 d	4.24 (m)	71.7 d	3.52 (m)	73.9 d
5”a	3.34 (t, 8.7)	78.2 d	3.34 (m)	78.1 d	3.34 (m)	78.1 d	3.94 (m)	78.7 d	1.91 (m)	36.4 t
5”b									1.35 (m)	
6”a	3.87 (dd, 12.1, 2.0)	62.8 t	3.86 (dd, 12.4, 1.9)	62.8 t	3.87 (dd, 12.0, 2.0)	62.8 t	4.55 (dd, 2.5, 12.5)	62.8 t	3.56 (m)	65.6 t
6”b	3.65 (dd, 11.9, 5.5)		3.65 (dd, 11.9 5.5,)		3.65 (dd, 11.9, 5.5)		4.37 (m)		3.56 (m)	
R_10_-1”'	4.45 (q, 1.9)	103.8 d	4.52 (t, 5.1)	102.0 d	4.52 (t, 5.1)	102.0 d				
2”'	1.38 (m)	45.4 d	1.51 (m)	38.2 t	1.52 (m)	38.2 t				
3”'a	1.50 (m)	22.6 t	1.39 (m)	18.2 t	1.40 (m)	18.2 t				
3”'b	1.32 (m)				0.91 (t, 7.4)	14.3 q				
4”'a	1.46 (m)	29.2 t	0.90 (t, 7.4)	14.3 q						
4”'b	1.27 (m)									
5”'	1.28 (m)	30.6 t								
6”'	1.28 (m)	24.2 t								
7”'	0.89 (d, 7.8)	14.4 q								
2”'-Me	0.88 (d, 7.4)	12.0 q								

*Patrinoside C (**6**)*: light yellow oil; [α]^24^_D_-61.4 (*c*0.14, MeOH); UV (MeOH) λ_max_ (log ε): 209 (4.16) nm, 217 (4.18) nm; IR (KBr) ν_max_ 3,431, 2,926, 2,876, 1,727, 1,637, 1,384, 1,075 cm^−1^; ^1^H and ^13^C NMR data, see Table 2; positive ESIMS *m/z* 537 [M + Na]^+^; HREIMS *m/z* 537.2302 [M + Na]^+^ (calcd for C_25_H_38_O_11_Na, 537.2306).

*Patrinoside D (**7**)*: light yellow oil; [α]^24^_D_-54.2 (*c*0.05, MeOH); UV (MeOH) λ_max_ (log ε): 209 (4.06) nm, 217 (4.06) nm; IR (KBr) ν_max_ 3,429, 2,924, 2,855, 1,724, 1,635, 1,384, 1,075 cm^−1^; ^1^H and ^13^C NMR data, see Table 2; positive ESIMS *m/z* 551 [M + Na]^+^; HREIMS *m/z* 551.2467 [M + Na]^+^ (calcd for C_26_H_40_O_11_Na, 551.2463).

*Patrinoside E (**8**)*: light yellow oil; [α]^23^_D_-23.7 (*c*0.08, MeOH); UV (MeOH) λ_max_ (log ε): 205 (3.95) nm, 214 (3.94) nm; IR (KBr) ν_max_ 3,429, 2,925, 2,857, 1,725, 1,635, 1,384, 1,077 cm^−1^; ^1^H and ^13^C NMR data, see Table 2; positive ESIMS *m/z* 497 [M + Na]^+^; HREIMS *m/z* 497.1988 [M + Na]^+^ (calcd for C_22_H_34_O_11_Na, 497.1993).

*Patrinoside F (**9**)*: light yellow oil; [α]^24^_D_-74.0 (*c*0.32, MeOH); UV (MeOH) λ_max_ (log ε): 207 (4.18) nm; IR (KBr) ν_max_ 3,441, 2,925, 2,860, 1,727, 1,639, 1,121, 1,065 cm^−1^; ^1^H and ^13^C NMR data, see Table 2; positive ESIMS *m/z* 467 [M + Na]^+^; HREIMS *m/z* 467.1889 [M + Na]^+^ (calcd for C_26_H_40_O_11_Na, 467.1888).

*Patrirscabiobisin D (**10**)*: light yellow oil; [α]^23^_D_-34.1 (*c*0.17, MeOH); UV (MeOH) λ_max_ (log ε): 209 (4.24) nm, 217 (4.25) nm; IR (KBr) ν_max_ 3,426, 2,926, 1,724, 1641, 1,077 cm^−1^; ^1^H and ^13^C NMR data, see Table 3; positive ESIMS *m/z* 663 [M + Na]^+^; HREIMS *m/z* 663.2627 [M + Na]^+^ (calcd for C_31_H_44_O_14_Na, 663.2623).

**Table 3:** The ^1^H and ^13^C NMR data of **10** (CD_3_OD δ in ppm, *J* in Hz).

**Position**	**δ_H_**	**δ_C_**
1	5.80 (d, 5.7)	93.3 d
3	6.35 (s)	139.9 d
4		117.2 s
5	3.11 (q, 8.0)	34.9 d
6a	2.04 (m)	39.3 t
6b	1.66 (m)	
7	3.26 (m)	77.9 d
8	2.45 (m)	48.3 d
9	2.46 (m)	41.8 d
10a	3.93 (d, 11.9)	66.9 t
10b	3.78 (dd, 12, 3.1)	
11a	4.27 (dd, 7.8, 3.4)	69.6 t
11b	4.06 (dd, 12.8, 4.2)	
R_1_-1'		166.3 s
2'	5.70 (t, 1.3)	116.0 d
3'		161.3 s
4'	1.93 (d, 1.0)	27.6 q
5'	2.18 (d, 1.0)	20.6 q
R_11_-1”	4.27 (d, 7.8)	103.3 d
2”	3.19 (dd, 9.0, 7.9)	75.1 d
3”	3.35 (m)	78.1 d
4”	3.26 (m)	71.7 d
5”	4.01 (t, 3.7)	78.8 d
6”a	3.86 (dd, 12.6, 1.9)	62.8 t
6”b	3.65 (11.9, 5.5)	
R_10_-1”'	4.07 (d, 4.2)	102.3 d
3”'	9.54 (s)	196.9 d
4”'		151.7 d
5”'	3.45 (m)	37.5 d
6”'a	2.09 (m)	39.4 t
6”'b	1.66 (m)	
7”'	4.38 (t, 4.2)	73.2 d
8”'	2.20 (m)	46.1 d
9”'	1.60 (m)	42.1 d
10”'	3.73 (d, 7.0)	63.5 t
11”'a	6.25 (s)	134.1 t
11”'b	6.15 (s)	

### Cytotoxicity Assays

The following human tumor cell lines were used: HL-60, SMMC-7721, A-549, MCF-7, and SW-480. These were obtained from ATCC (Manassas, VA, USA). All the cells were cultured in RPMI-1640 or DMEM medium (Hyclone, Logan, UT, USA), supplemented with 10% fetal bovine serum (Hyclone) at 37°C in a humidified atmosphere with 5% CO_2_. Cell viability was assessed by conducting colorimetric measurements of the amount of insoluble formazan formed in living cells based on the reduction of 3-(4,5-dimethylthiazol-2-yl)-5-(3-carboxymethoxyphenyl)-2- (4-sulfopheny)-2H-tetrazolium (MTS) (Sigma, St. Louis, MO, USA) (Monks et al., 1991). In brief, 100 μL of adherent cells were seeded into each well of a 96-well cell culture plate and allowed to adhere for 12 h before drug addition, while suspended cells were seeded just before drug addition, both with an initial density of 1 × 10^5^ cells/mL in 100 μL medium. Each tumor cell line was exposed to the test compound at various concentrations in triplicate for 48 h, with cisplatin and paclitaxel (Sigma) as positive controls. After the incubation, MTS (100 μg) was added to each well, and the incubation continued for 4 h at 37 °C. The cells were lysed with 100 μL of 20% SDS-50% DMF after removal of 100 μL medium. The optical density of the lysate was measured at 490 nm in a 96-well microtiter plate reader (Bio-Rad 680). The IC50 value of each compound was calculated by the Reed and Muench method (1938).

### Computational Study

The CHARMM force field and DFT/TDDFT calculations were performed with Discovery Studio 4.0 and Gaussian09 program package, respectively. Conflex conformational search generated low-energy conformers within a 20 kcal/mol energy window and were subjected to geometry optimization using DFT method without imposing any symmetry constraints at the B3LYP/6-31G(d) level. Frequency calculations were carried out using the same level to verify that the molecular structures were true minimum. The calculated ECD spectra were generated by the program SpecDis2 using a Gaussian band shape with 0.3eV exponential half-width from dipole-length dipolar and rotational strengths (Bruhn et al., 2013).

## Results and Discussion

**Figure 2:**
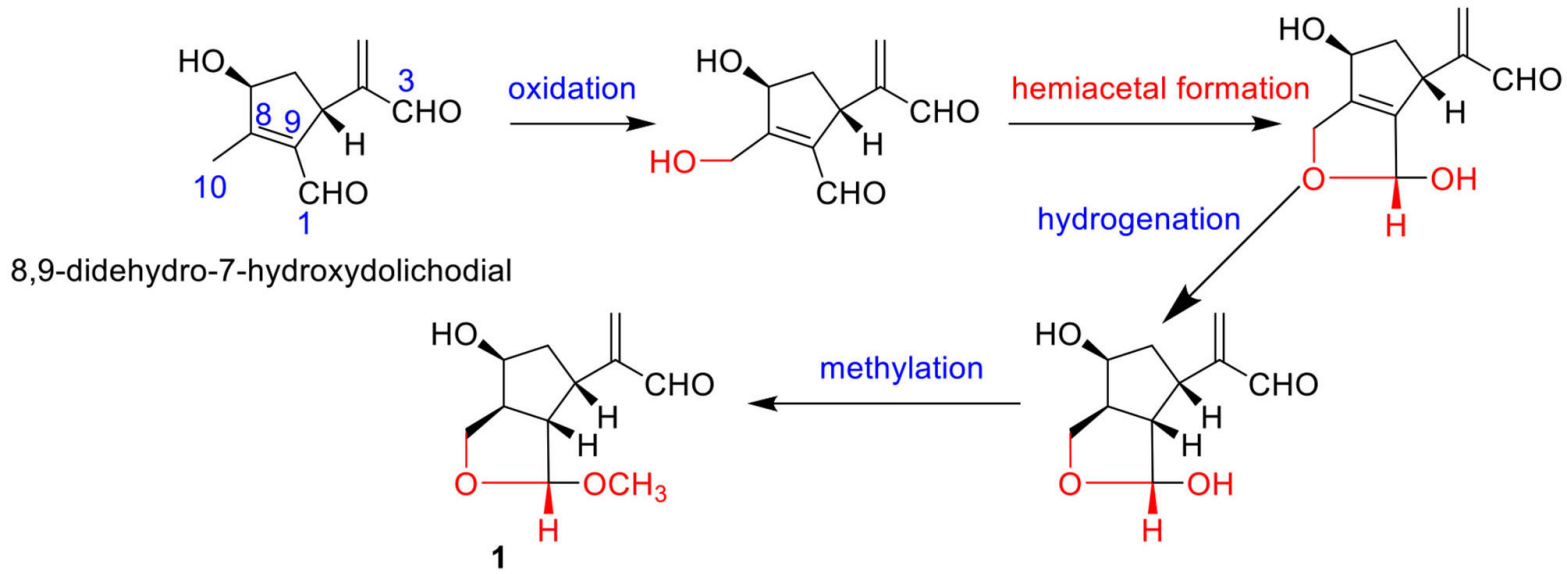
The possible pathway for the transformation of compound **1**.

**Figure 3:**
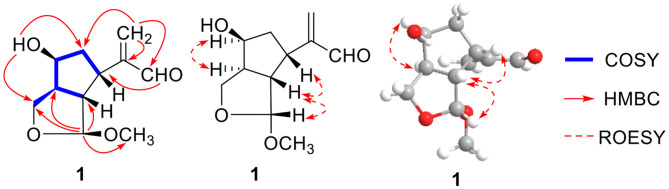
Key ^1^H-^1^H COSY, HMBC, and ROESY correlations of compound **1**.

Compound **1** was indicated as C_11_H_16_O_4_ by HR-ESI-MS [m/z 235.0943 [M + Na]^+^, calcd. for 235.0941]. Preliminary inspection of the ^1^H and ^13^C NMR (Table 1) spectroscopic data of compound **1** revealed the presence of one conjugated aldehyde group [δ_H_ 9.55 (s), δ_C_ 196.3 (d)] and a pair of double bond [δ_C_ 154.1 (s), δ_C_ 134.5 (t)], two oxygenated methine groups [δ_H_ 4.89 (s), δ_C_ 111.2 (d); δ_H_ 4.26 (q), δ_C_ 73.3 (d)], and one oxygenated methylene at δ_C_ 66.6 (t). These data were similar to those of 8,9-didehydro-7-hydroxydolichodial (Veith et al., 1986), except for the disappearance of conjugated aldehyde and double bonds, and the appearance of two methines and one oxygenated methane, especially the change of methyl at C-10 to oxygenated methylene. Therefore, we speculated that the methyl at C-10 of 8,9-didehydro-7-hydroxydolichodial was oxidized to oxygenated methylene, and then underwent a hemiacetal formation with the aldehyde group at C-1 to form the deformed iridoid—compound **1** with 5/5 rings, which occurred simultaneously to the hydrogenation at C-8 and C-9, and finally the C-1 was methylated (Figure 2). This conclusion was confirmed by HMBC spectrum with the key correlations from H-1 [δ_H_ 4.89 (s)] to C-10 (δ_C_ 66.6), C-8 (δ_C_ 48.6), and C-9 (δ_C_ 57.1), and OMe (δ_C_ 54.5) were observed, and the correlations of H-1/H-9 and H-9/H-5 in the ROESY spectrum (Figure 3).

**Figure 4:**
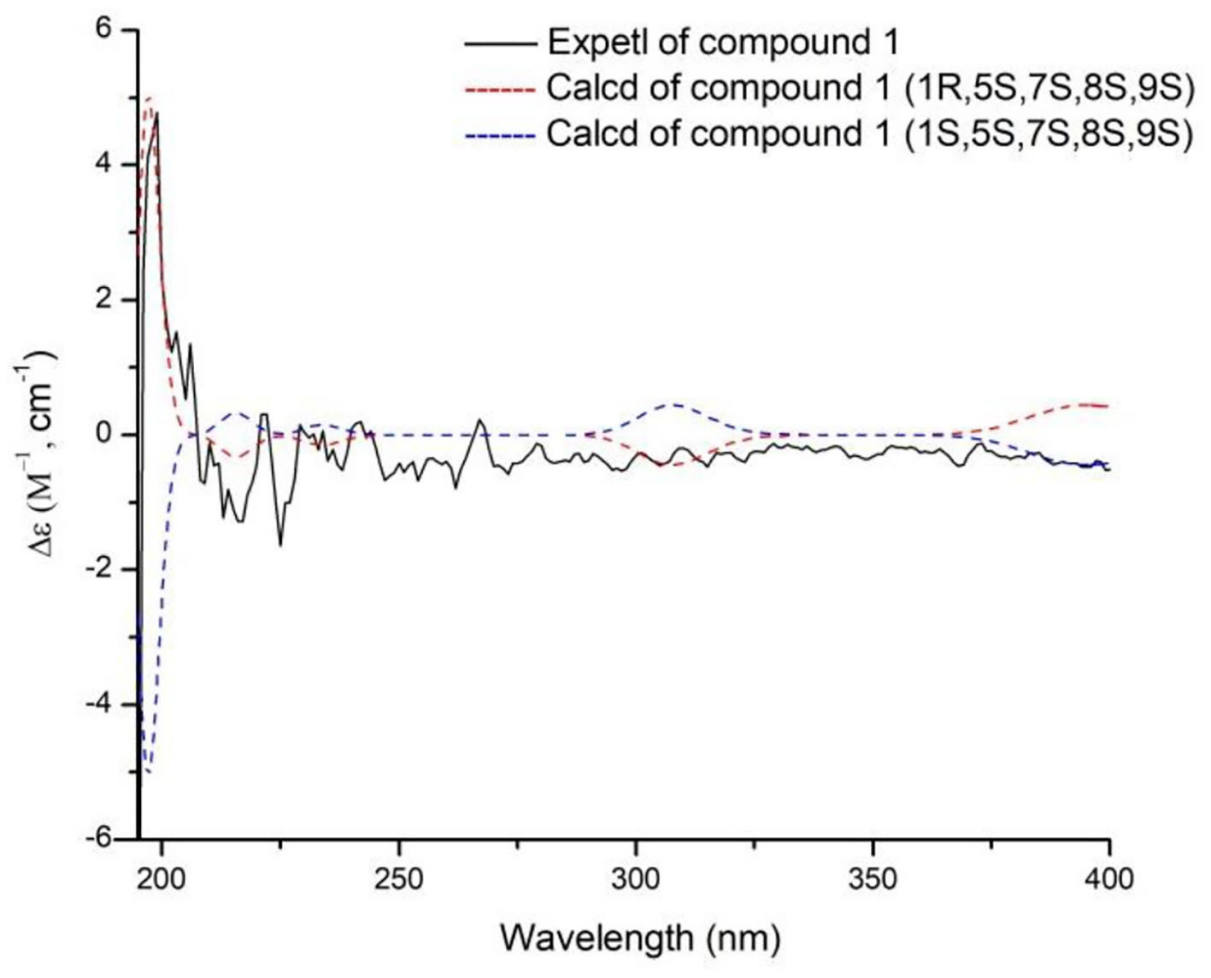
Calculated and experimental ECD spectra of compound **1** at TDDFT/B3LYP/6-31G(d) level.

The absolute configurations of H-5 and H-7 were both *S* deduced from the comparison of chemical shift value to the known compound 8,9-didehydro-7-hydroxydolichodial (Veith et al., 1986). The α-orientation of H-8 was elucidated by the correlation of H-7 with H-8 in ROESY (Figure 3). The β-orientations of H-1 and H-9 were confirmed by the ROESY correlations from H-5 to H-1 and H-9 (Figure 2). And the *R*-configuration of the new formed acetal methine at C-1 was further confirmed by the comparison of experimental and calculated ECD spectra, which the positive Cotton effect near 200 nm in calculated ECD spectra of *R*-configuration at C-1 agreed with the experimental ECD spectra (Figure 4). Hence, the structure of compound **1** was defined as (1*R*,5*S*,7*S*,8*S*,9*S*)-1,10-epoxy-7,10-dihydroxy-dolichodial, named Patriscabioin M.

**Figure 5:**
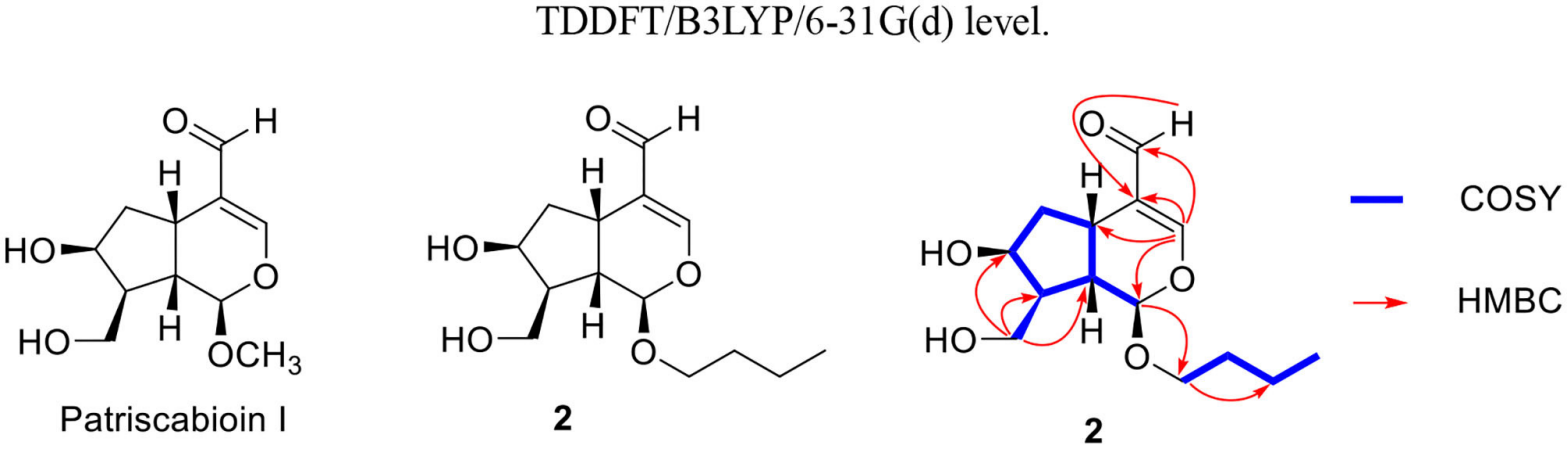
Key ^1^H-^1^H COSY and HMBC correlations of compound **2** and the structure of patriscabioin I.

Compound **2**, a light yellow oil, had a molecular formula of C_14_H_22_O_5_ based on HRESIMS ([M + Na]^+^, *m/z* 293.1354, calcd. for 293.1359). Detailed comparisons of ^1^H-NMR and ^13^C-NMR (Table 1) suggested it was a typical iridoid and included a hemiketal methine at δ_H_ 4.92 (d, *J* = 5.7 Hz, H-1) and δ_C_ 104.1 (d, C-1), a conjugated olefinic bond at δ_C_ 193.3 (d, C-11); δ_C_ 164.1 (d, C-3), δ_C_ 125.6 (s, C-4), and one oxygenated methylene at δ_C_ 62.3 (t, C-10). These data were similar to patriscabioin I (Liu et al., 2017), except for the substituent at C-1 (Figure 5). In compound **2** it was *n*-butanol group [δ_C_ 70.7 (t), 32.7 (t), 20.3 (t), 14.1 (q)], while it was methyl in patriscabioin I. The correlation from H-1 [4.92 (d, 5.7)] to C-1' [δ_C_ 70.7 (t)] in HMBC implied the location of *n*-butanol group at C-1 (Figure 5). The configuration of compound **2** was the same as patriscabioin I. Therefore, the structure of compound **2** was defined as (1*R*,5*S*,7*S*,8*S*,9*S*)-1-*n*-butoxy-7,10-dihydroxy-11-aldehyde-5,6-dihydrovaltrate hydrin, named patriscabioin N.

**Figure 6:**
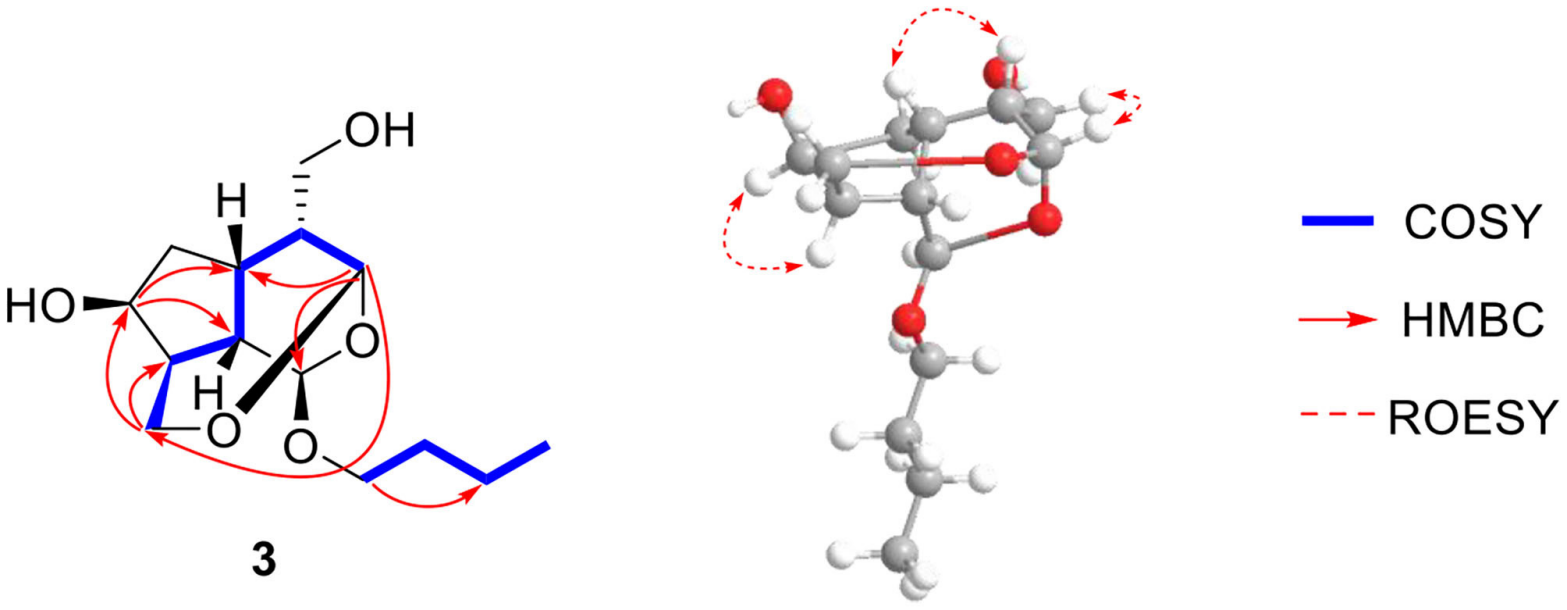
Key ^1^H-^1^H COSY, HMBC, and ROESY correlations of compound **3**.

**Figure 7:**
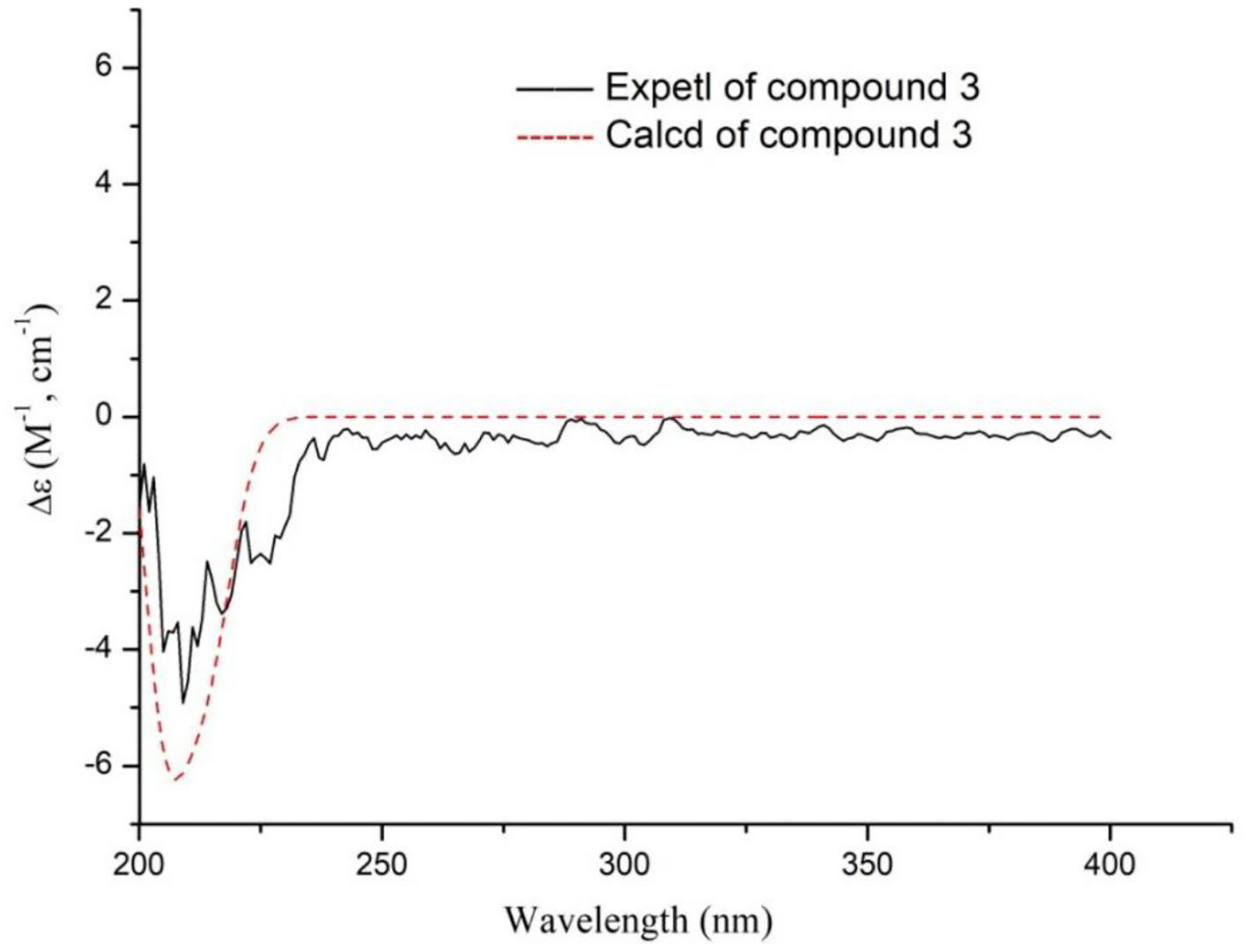
Calculated and experimental ECD spectra of compound **3** at TDDFT/B3LYP/6-31G(d) level.

Compound **3** was found to have a molecular formula of C_14_H_24_O_5_, determined by its positive HRESIMS data ([M + Na]^+^, *m/z* 295.1515, calcd. for 295.1516) and ^13^C-NMR spectrum (Table 1), with 3 degrees of unsaturation. From the NMR data, compound **3** showed spectral characteristics of iridoid at δ_H_ 5.19 (d, *J* = 2.8 Hz, H-1) and δ_C_ 99.5 (d, C-1), with a n-butanol group [δ_C_ 67.5 (t), 32.2 (t), 19.7 (t), 14.0 (q)]) located in C-1, which was confirmed by the correlation from H-1 to C-1' in HMBC (Figure 6). However, the biggest difference from other iridoids' skeletons (such as compounds **2**, **5**-**9**) was the added 1 unsaturation, of which the former's degree was 2, and the latter was 3. Meanwhile, the NMR spectra exhibited the disappeared double bonds and the appearance of two methines, among which the chemical shift was δ_C_ 94.7 (d, C-3), but there was no other unsaturated group. Therefore, it was presumed that there was a ring that existed in the skeleton of compound **3**. The new ring was formed by the ether bond between C-3 and C-10, which was further verified by the fragment of H-3—H-4—H-5—H-9—H-8—H-10 in COSY, and the correlation from H-3 to C-10 in HMBC (Figure 7). According to the *S*-configuration of H-5 and H-9 and *R*-configuration of H-1, the configuration of H-4 was determined as *R*-configuration and the configurations of H-3 and H-8 were determined as *S*-configuration by the correlations of H-4/H-5, H-3/H-11, and H-7/H-8 in ROESY (Figure 6). There was good agreement between experimental and calculated ECD spectra, which was further verified these configurations (Figure 7). Hence, the structure of **3** was established as (1*R*,3*S*,4*R*,5*S*,7*S*,8*S*,9*S*)-1*-n*-butoxy-3,10-epoxy-7-hydroxy-3,4,5,6-tetrahydrovaltrate hydrinm, named patriscabioin O.

**Figure 8:**
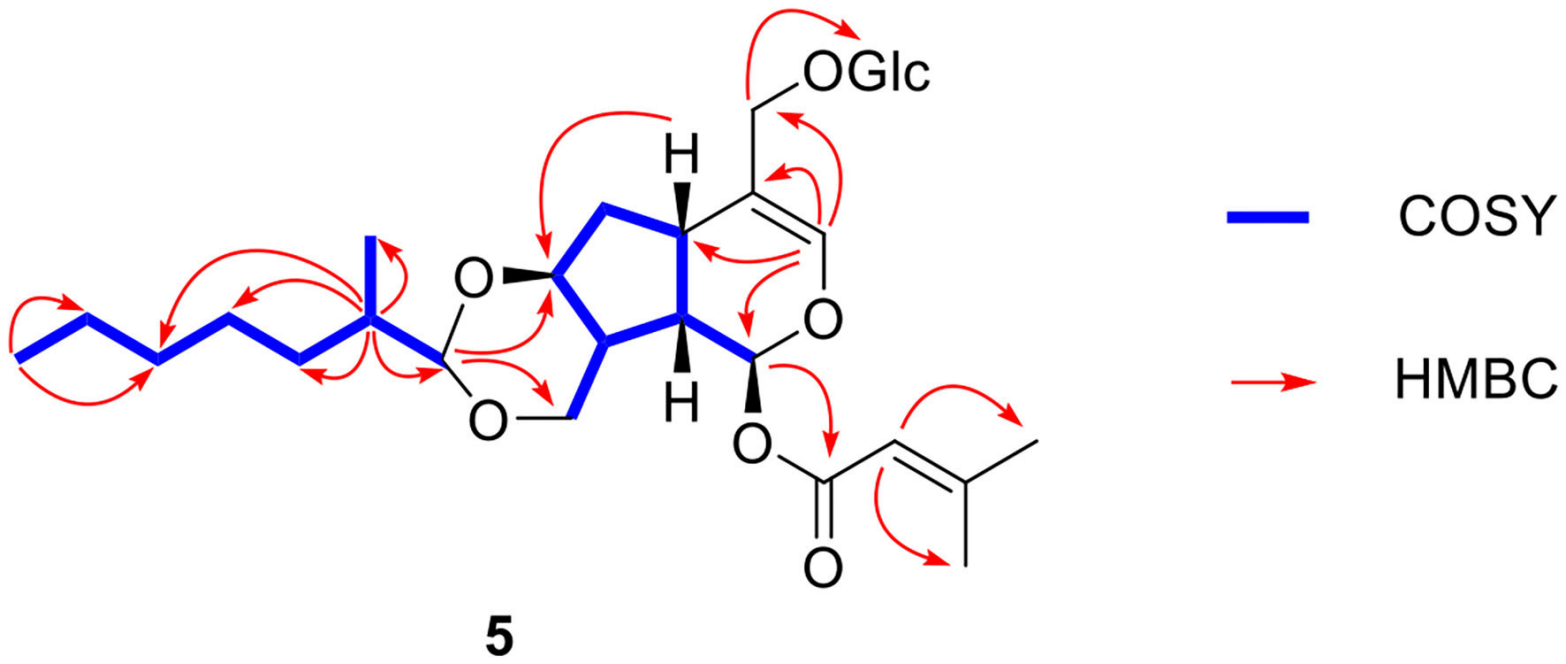
Key ^1^H-^1^H COSY and HMBC correlations of compound **5**.

Compound **5** was obtained as a slight yellow oil. Its molecular formula was assigned as C_29_H_46_O_11_ on the basis of positive-ion HR-ESI-MS at *m*/*z* 593.2931 [M + Na]^+^ (calcd for 593.2932) and ^13^C-NMR data (Table 2). Carefully analysis of ^1^H-NMR and ^13^C-NMR led to the discovery of a hemiketal methine at δ_H_ 5.90 (1H, d, *J* = 5.0 Hz, H-1) and δ_C_ 93.0 (d, C-1), a trisubstituted olefinic bond at δ_H_ 6.35 (1H, s, H-3), δ_C_ 139.8 (d, C-3), and δ_C_117.3 (s, C-4), an oxymethine at δ_C_ 79.3 (C-7) and two oxygenated methylenes at δ_C_ 67.1 (t, C-10) and δ_C_ 69.7 (t, C-11). The above data indicated the existence of 7,10,11-trihydroxy-3-en valtratehydrin. It was found that **5** had a 3-methylcrotonyl group at δ_C_ 166.4 (s), δ_C_ 116.1 (d), δ_C_ 161.2 (s), δ_C_ 27.6 (q), δ_C_ 20.6 (q) and a glucopyranose unit at δ_C_ 103.2 (d), δ_C_ 78.2 (d), δ_C_ 78.0 (d), δ_C_ 75.1(d), δ_C_ 71.7 (d), δ_C_ 62.8 (t) through further detailed analysis of its 1D-NMR and 2D-NMR. The 3-methylcrotonyl group and glucopyranose were arranged at C-1 and C-11 by the correlations from δ_H_ 5.90 (H-1) to δ_C_ 166.4 (C-1'), and δ_H_ 4.29 (H-1”) to δ_C_ 69.7 (C-11”) in HMBC spectrum (Figure 8). The typical signal for β-configuration (δ_H_ 4.29, d, *J* = 7.8 Hz) of H-1” could also be observed. Thus, eight carbons remained, including two methyl groups, four methylene groups, and two methine groups. The ^1^H-^1^H COSY correlations (Figure 8) showed the fragments: CH(H-1”')—CH(H-2”')—CH_2_ (H-3”')—CH_2_(H-4”')—CH (H-5”', H-6”')—CH_3_ (H-7”') and CH(H-2”')—CH_3_(2”'-Me). Combined with the correlations of H-2”'/2”'-Me, and H-1”'/ C-10, C-7 in HMBC, these revealed the presence of a 1,3-dioxane through acetalation between OH-7, OH-10 and C-1.”' It meant that 1,3-dioxane was formed by hemiacetal formation from hydroxyl groups of C-7 and C-10 and aldehyde of 2-methyl-heptaldehyde.

The relative configuration of **5** was similar to patriscabioins A-L and patriscabiobisins A and B (Liu et al., 2017a,b) from *P*. *scabiosaefolia* through comparison with their ROESY and CD spectrum (**Figure 11**). The ROESY cross peaks of H-1”'/H-8 and 2”'-Me/H-1”' suggested the α-orientation of H-1”' and 2”'-Me. Thus, the structure of **5** was identified as shown and named patrinoside B.

Compound **6** had the molecular formula C_25_H_38_O_11_ based on its quasimolecular ion peak at *m*/*z* 537.2302 [M + Na]^+^ (calcd for 537.2306) in HR-ESI-MS spectrum and ^13^C-NMR data (Table 2). Comparison of the ^13^C NMR and DEPT spectra of **6** with those of **5** revealed that **6** shared the same aglycone, a glucose at C-11, and the 3-methylcrotonyl group at C-1 as compound **5**, and the significant difference between them was the disappearance of two methylenes, one methine and one methyl in the high field region, and the molecular weight of **6** was 56 less than that of **5**. This indicated that the fragment of 2-methyl-heptaldehyde in compound **5** was changed to *n*-butyraldehyde in compound **6**, which was further verified by the correlations from H-1”' (δ_H_ 4.52) to C-2”' (δ_C_ 38.2), and H-4”' (δ_H_ 0.90) to C-2”' (δ_C_ 38.2), C-3”' (δ_C_ 18.2) in HMBC. Therefore, the structure of **6** was deduced as shown and named patrinoside C.

The molecular formula of **7** was identified as C_26_H_40_O_11_ from HR-ESI-MS at *m/z* 551.2467 [M + Na]^+^ (calcd for 551.2463). Comparing ^1^H-NMR and ^13^C-NMR data of **7** with those of **6**, it was found that they shared similar NMR data, and the obvious distinction between them was the appearance of a methylene (δ_C_ 34.8) and the change in chemical shift of methyl signal of C-4' (δ_C_ 27.6→ 12.3). It indicated that a 3-methylcrotonyl group at C-1 in **6** was replaced by the 3,4-dimethylcrotonyl group in **7**. This was ultimately confirmed by 1D and 2D NMR spectra. In HMBC spectrum, the correlations between H-2' (δ_H_ 5.68) and C-4' (δ_C_ 34.8) and C-5' (δ_C_ 12.3) provided solid evidence for the existence of the 3,4-dimethylcrotonyl group. Finally, the structure of **7** was elucidated as shown and named patrinoside D.

Compound **8** was analyzed to have a molecular formula of C_22_H_34_O_11_ by HR-ESI-MS at *m/z* 497.1988 [M + Na]^+^ (calcd for 497.1993), whose molecular weight is 54 less than that of **7**. Careful analysis of its NMR data found that it had the same aglycone as compound **7**, as well as a glucose at C-11 and a 3,4-dimethylcrotonyl group at C-1, but lacked an oxymethine at δ_C_ 102.0, two methylenes and a methyl, which suggested that it did not have a 1,3-dioxaneformed at C-7 and C-10. This could be further verified by the chemical shifts at C-7 and C-10 which were shifted up field from δ_C_ 79.2 to 72.7 and form δ_C_ 67.1 to 62.1, respectively, compared with compound **7**. Finally, the structure of compound **8** was characterized as (1*S*,5*S*,7*S*,8*S*,9*S*)-1-*O*-(3,4-dimethylcrotonyl)-7,10-dihydroxy-11-β-D-glucose-5,6-dihydrovaltrate hydrin and named patrinoside E.

**Figure 9:**
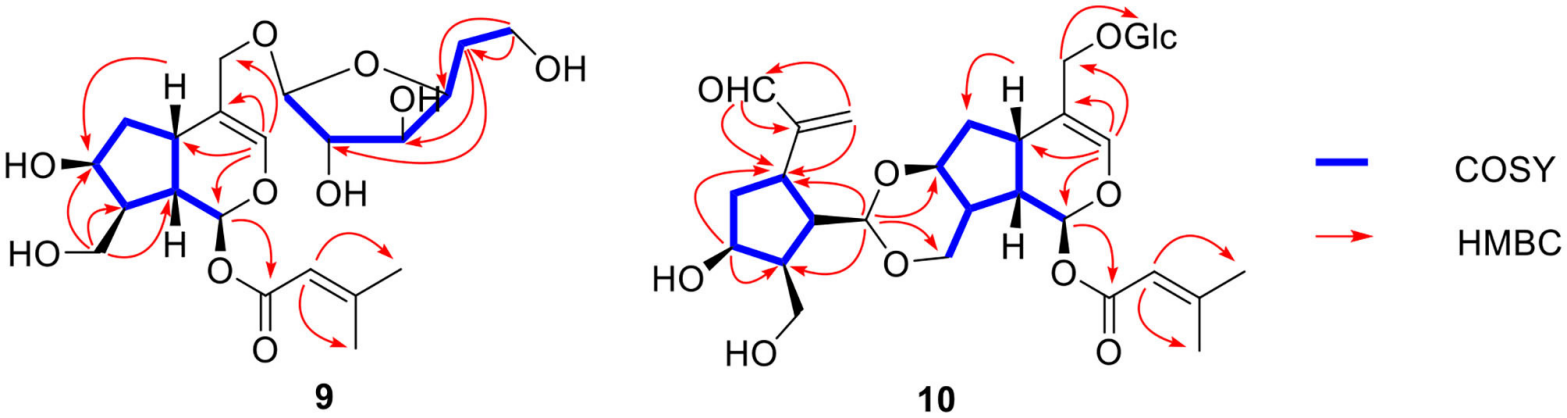
Key ^1^H-^1^H COSY and HMBC correlations of compounds **9-10**.

Compound **9** was found to have a molecular formula of C_21_H_32_O_10_ by HR-ESI-MS at *m/z* 467.1889 [M + Na]^+^ (calcd for 467.1888). Careful analysis of its 1D and 2D NMR data led to finding that it was a 7,10,11-trihydroxy-3-envaltratehydrin iridoid with a 3-methylcrotonyl group at C-1 and a sugar. The ^1^H-^1^H COSY correlations (Figure 9) showed a fragment: CH (H-1”, δ_H_ 4.22)—CH (H-2”, δ_H_ 3.09)—CH (H-3”, δ_H_ 3.59)—CH (H-4”, δ_H_ 3.52)—CH_2_ (H-5”, δ_H_ 1.35)—CH_2_ (H-6”, δ_H_ 3.56). Combined with the correlations between H-5” (δ_H_ 1.35) and C-2” (δ_C_ 76.9) and C-3” (δ_C_ 72.2) in the HMBC, all these signals hinted the sugar was 5-deoxyglucose, which was linked to C-11 from the correlation of H-11 (δ_H_ 4.06, 4.24)/C-1” (δ_C_ 103.8). The β-configuration was determined by the coupled constant of H-1” (δ_H_ 4.22, d, *J* = 7.8 Hz). Ultimately, the structure of compound **9** was confirmed as (1*S*,5*S*,7*S*,8*S*,9*S*)-1-*O*-(3-methylcrotonyl)-7,10-dihydroxy-11-(5-deoxy-β-D-glucofuranose)-5,6-dihydrovaltrate hydrin and named patrinoside F.

Compound **10** was formulated as C_31_H_44_O_14_ from HR-ESI-MS at *m/z* 663.2627 [M + Na]^+^ (calcd for 663.2623) and ^13^C-NMR data (Table 3). The carbon resonances at δ_C_ 103.3 (d), 75.1 (d), 78.1 (d), 71.7 (d), 78.8 (d), 62.8 (t) and the anomeric proton signals at δ_H_ 4.27 (d, *J* = 7.8 Hz) suggested that compound **10** contained a glucose at C-11 by the correlation from the anomeric proton (δ_H_ 4.27) to C-11 (δ_C_ 69.6) in HMBC (Figure 9). The remaining carbon signals showed two iridoid units, which means it was a bis-iridoidglycoside. Detailed analysis of the NMR data at δ_C_ 93.3 (d), δ_C_ 139.9 (d), δ_C_ 117.2 (s), δ_C_ 77.9 (d), δ_C_ 66.9 (t), δ_C_ 69.6 (t), and δ_H_ 5.80 (d, 5.7), combined with the 3-methylcrotonyl [δ_C_ 166.3 (s), δ_C_ 161.3 (s), δ_C_ 116.0 (d), δ_C_ 27.6 (q), δ_C_ 20.6 (q)] at C-1, found one iridoid was patrinoside A (Liu et al., 2019). The other iridoid was similar to 8,9-didehydro-7-hydroxydolichodial (Georg and Joerg, 1985) through the conjugated aldehyde and double bond at δ_C_ 196.9 (d), δ_C_ 151.7 (s), δ_C_ 134.1 (t), two methylene at δ_C_ 39.4 (t), and δ_C_ 63.5 (t), and four methynes at δ_C_ 37.5 (t), δ_C_ 73.2 (d), δ_C_ 46.1 (d), δ_C_ 42.1 (d) in ^13^C NMR, except for the absence of one methyl, one pair of double bonds, and an aldehyde group. However, it did include an oxygenated methylene, two methines, and an acetal at δ_C_ 102.3 (d). This suggested that the other iridoid was 7,10-dihydroxydolichodial. And two units were linked through the aldehyde group between the aldehyde group at C-1' of 7,10 -dihydroxydolichodial and hydroxyl groups at C-7 and C-10 of patrinoside A, which was further verified by the correlations of H-1'/C-7, C-10 in the HMBC spectrum. The α-orientations of H-1”', H-8”', and H-9”' were determined by the ROESY correlations of H-7”' with H-1”', H-8”,' and H-9”'. Additionally, these were further proven by the good agreement of ECD spectra between compound **10** and patriscabiobisin A and B which were similar to compound **10**. Thus, the structure of compound **10** was characterized as shown, namely patriscabiobisin C.

**Figure 10:**
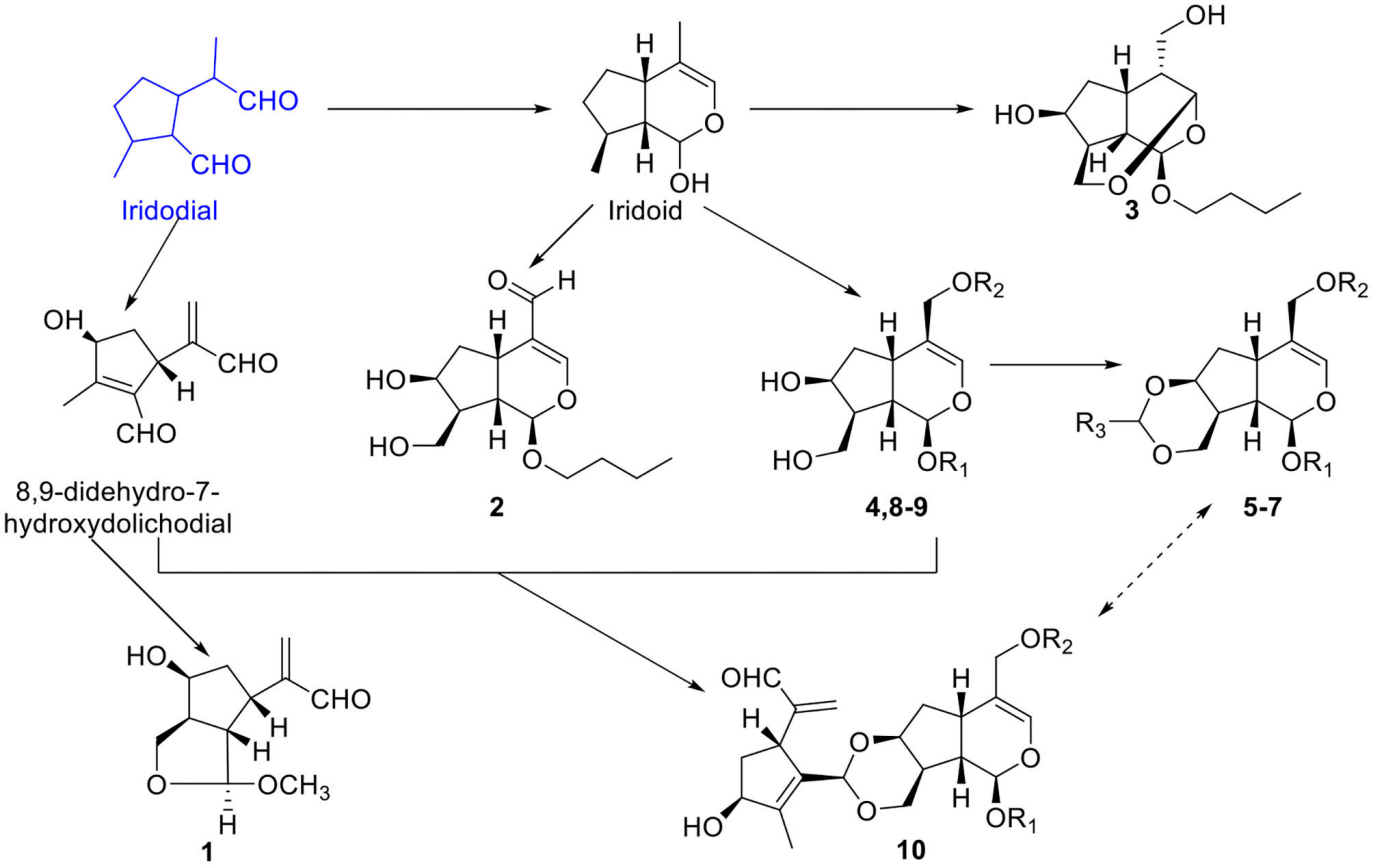
The possible pathway for the transformation of compounds **1**-**10**.

**Figure 11:**
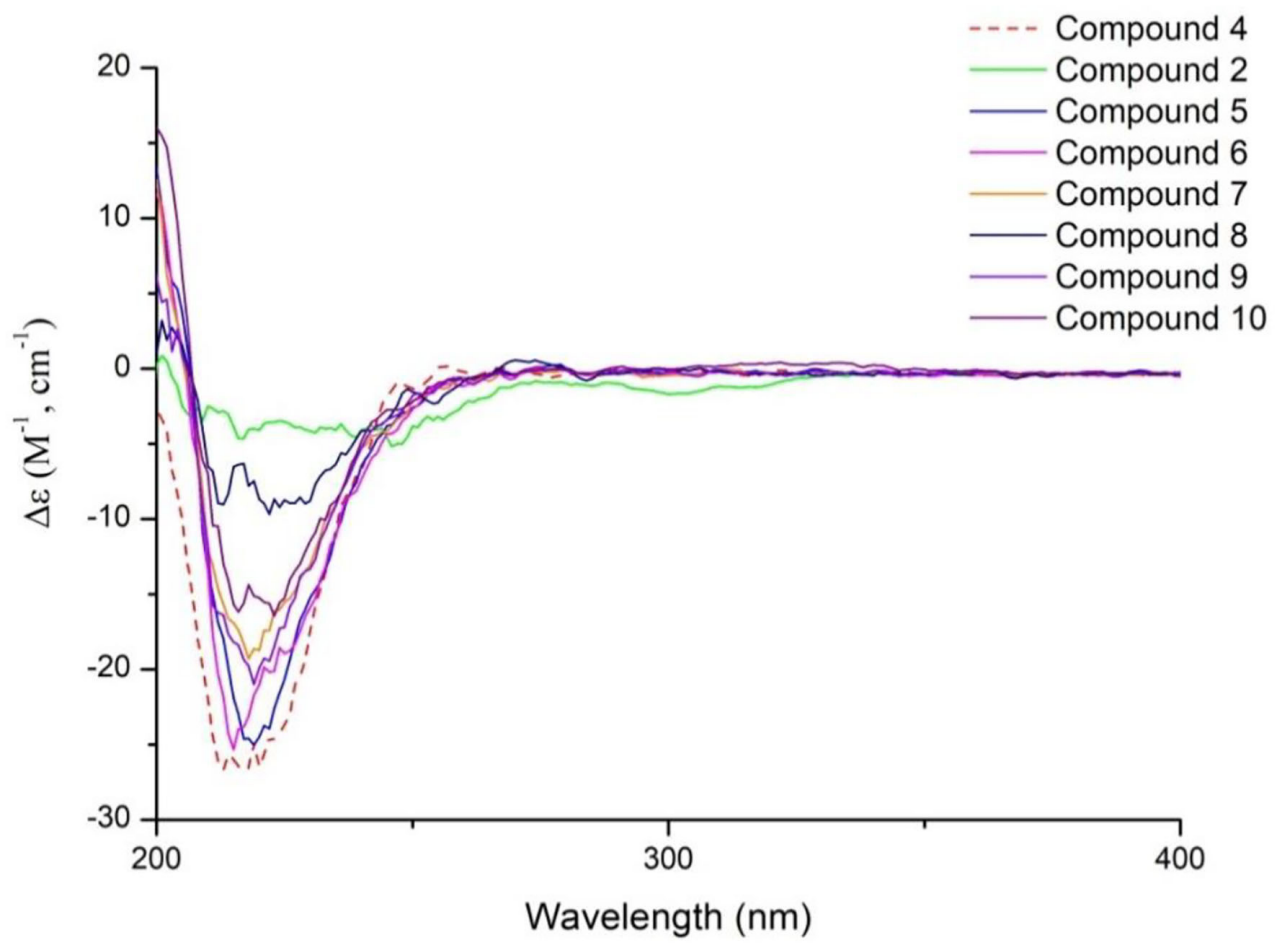
Experimental ECD spectra of compounds **2**, **4**-**10** at TDDFT/B3LYP/6-31G(d) level.

In conclusion, compounds **1**-**10** were a series of 5,-dihydrovaltrate hydrins with characteristic substituents, such as 3-methylcrotonyl group or 3,4-dimethylcrotonyl group in the Valerianaceae family, belonging to iridoids derived from iridodial. Iridoial was enoxylated and then subjected to intramolecular hemiacetal formation to form iridoid. And iridoid suffered a series of chemical changes to achieve diverse compounds (**1**-**10**) (Figure 10). Iridodial was oxidized and then underwent the hemiacetal formation between C-1 and C-10 to form the deformed iridoid—compound **1** with 5/5 rings. Iridoid was oxidized and combined with different substituents to constitute compounds **2**, **4**, **8**-**9**, and when it was dehydration condensed between C-10 and C-3 to generate a new ring, like compound **3**. However, when the hydroxyl of C-7 and C-10 of iridoid combined with the aldehyde compounds, a 1,3-dioxane group would be formed, such as compounds **5**-**7**. Furthermore, if the aldehyde group came from an iridoid, then it would generate a bis-iridoid, like compound **10**. And the configurations of compounds **1**-**10** were further confirmed by ECD (Figure 11).

**Figure 12:**
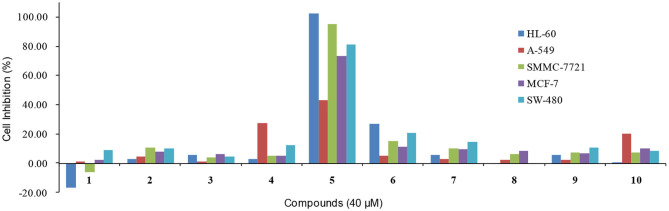
The cytotoxic activities against five human cancer cell lines of compounds **1**-**10**.

Finally, the cytotoxic activities of these compounds were evaluated against five human cancer cell lines (HL-60, A-549, SMMC-7721, MCF-7, and SW-480). Unfortunately, none of the compounds showed significant cytotoxicities at 40 μM, except for compound **5** which showed the cell inhibition of 102.42, 95.13, 73.07, and 80.93% against HL-60, SMMC-7721, MCF-7, and SW-480 respectively (Figure 12).

## Conclusion

*P. scabiosaefolia* is a medical and edible Chinese herb with high nutritional value and a wide range of biological activities. Research showed plants in the *Patrinia* genus are rich in iridoids and terpenoids. In our ongoing study, we found a series of iridoids and iridoid glycosides, including three new iridoids (**1**-**3**) and six novel irioid glycosides (**5**-**10**), and one known compound (**4**). Among them, compound **1** was a deformed iridoid, compound **3** formed a cycle between C-3 and C-10, compounds **5**-**7** with a 1,3-dioxane between C-7 and C-10, and compound **10** was a bis-iridoid glycoside, which was the first reported in *P*. *scabiosaefolia*. Cytotoxicity assays found compound **5** showed good cell inhibition against HL-60, SMMC-7721, MCF-7, and SW-480. All these results enriched the study on the chemical constituents and activities of *Patrinia* genus. However, the activities of these compounds were not thorough, just the evaluation of the cytotoxicity assays, and the other activities and their mechanisms are worth further exploration.

## Data Availability Statement

The original contributions presented in the study are included in the article/Supplementary Material, further inquiries can be directed to the corresponding author/s.

## Author Contributions

ZL, JH, and WK conceived the research subject. ZL, YN, LZ, LM, SC, and MW contributed to the development and writing of the manuscript. WK and ZL contributed in validating, reviewing, and supervising the project. All authors contributed to the article and approved the submitted version.

## Conflict of Interest

The authors declare that the research was conducted in the absence of any commercial or financial relationships that could be construed as a potential conflict of interest.

## References

[B1] Bruhn T. Schaumlöffel A. Hemberger Y. Bringmann G. (2013). Specdis: quantifying the comparison of calculated and experimental electronic circular dichroism spectra. Chirality 25, 25243–25249. 10.1002/chir.22138 23532998

[B2] Delectis Flora Reipublicae Popularis Sinicae Agendae Academiae Sinicae Edita. (1986). Flora of China. Beijing: Science Press. 73:006.

[B3] Georg S. Joerg V. (1985). Valepotriat-artefakte aus centranthus ruber (L.) Dc. Arch. Pharm. 318, 515–519. 10.1002/ardp.19853180607

[B4] Kim J. S. Kang S. S. (2013). Chemical constituents of plants from the genus Patrinia. Nat. Prod. Sci. 19, 77–119. Available online at: http://www.e-nps.or.kr/.

[B5] Liu Z. H. Hou B. Yang L. Ma R. J. Li J. Y. Hu. (2017a). Iridoids and bis-iridoids from *Patrinia scabiosaefolia*. RSC Adv. 7, 24940–24949. 10.1039/C7RA03345A

[B6] Liu Z. H. Ma R. J. Yang L. Li J. Y. Hu J. M. Zhou J. (2017b). Triterpenoids and iridoids from *Patrinia scabiosaefolia*. Fitoterapia 119, 130–135. 10.1016/j.fitote.2017.04.011 28456554

[B7] Liu Z. H. Xu L. T. Xu X. Q. Niu Y. Saadeldeen F. S. A. Kang W. Y. (2019). Effects and mechanisms of iridoid glycosides from *Patrinia scabiosaefolia*. Food Chem. Toxicol. 134, 110806–110811. 10.1016/j.fct.2019.110806 31521635

[B8] Monks A. Scudiero D. Skehan P. Shoemaker R. Paull K. Vistica. (1991). Feasibility of a high-flux anticancer drug screen using a diverse panel of cultured human tumor cell lines. J. Natl. Cancer Insit. 83, 757–766. 10.1093/jnci/83.11.757 2041050

[B9] Reed L. J. Muench H. (1938). A simple method of estimating fifty percent endo-points. Amer. J. Hygiene. 27, 493–797. 10.1093/oxfordjournals.aje.a118408

[B10] Veith J. Schneider G. Lemmer B. (1986). The influence of some degradation products of valepotriates on the motor activity of light-dark synchronized mice. Planta Med. 3, 179–183. 10.1243/PIME_PROC_1954_168_069_02 3749340

[B11] Xiao M. Zhu S. Zhang A. (2007). Research advance in medicinal and edible utilization of plants of *Parinia*. J. Jinling Inst. Technol. 23, 83–86. 10.3969/j.issn.1672-755X.2007.03.021

[B12] Zhong S. Li G. Lin H. Jin S. Qian X. (2001). An analysis of nutrient constituents of wild *Patrinia villosa*. Chin. Wild Plant Resour. 20, 45–46. 10.3969/j.issn.1006-9690.2001.01.019

