# Supplementary material for: Nine Unique Iridoids and Iridoid Glycosides From Patrinia scabiosaefolia
Source: Front Chem. 2021 Mar 29;9:657028. doi: 10.3389/fchem.2021.657028 (PMC8039314; doi:10.3389/fchem.2021.657028)
Supplement: Supplementary file 1 [file Data_Sheet_1.docx]

Supplementary Materials

**Nine unique iridoids and iridoid glycosides from *Patrinia scabiosaefolia***

Zhenhua Liu^a,b,c^, Yun Niu^a,d^, Li Zhou^a,d^, Lijun Meng^a,d^, Sitan Chen^a,d^, Mengke Wang^a,d^, Jiangmiao Hu^b,c,*^, Wenyi Kang^a,d,*^

**Affiliation**

^a^ National Center for Research and Development of Edible Fungus Processing Technology, Henan University, Kaifeng 475004, China;

^b^ State Key Laboratory of Phytochemistry and Plant Resources in West China, Kunming Institute of Botany, Chinese Academy of Sciences, Kunming 650201,China

^c^ University of Chinese Academy of Sciences, Beijing 100049, China

^d^ Joint International Research Laboratory of Food & Medicine Resource Function, Henan Province, Henan University, Kaifeng 475004, China;

*Corresponding author at: National Center for Research and Development of Edible Fungus Processing Technology, Henan University, Kaifeng 475004, China;

State Key Laboratory of Phytochemistry and Plant Resources in West China, Kunming Institute of Botany, Chinese Academy of Sciences, Kunming, Yunnan, China

*E-mail address:* [kangweny@hotmail.com](mailto:kangweny@hotmail.com); [hujiangmiao@mail.kib.ac.cn](mailto:hujiangmiao@mail.kib.ac.cn)

.

| No. | Contents | Page |
| --- | --- | --- |
| 1 | Figure 1.^1^H NMR spectrum of Patriscabioin M (**1**) in CD_3_OD | 5 |
| 2 | Figure 2.^13^C NMR spectrum of Patriscabioin M (**1**) in CD_3_OD | 5 |
| 3 | Figure 3. HSQC spectrum of Patriscabioin M (**1**) in CD_3_OD | 6 |
| 4 | Figure 4. HMBC spectrum of Patriscabioin M(**1**) in CD_3_OD | 6 |
| 5 | Figure 5.^1^H-^1^H COSY spectrum of Patriscabioin M (**1**) in CD_3_OD | 7 |
| 6 | Figure 6. ROESY spectrum of Patriscabioin M (**1**) in CD_3_OD | 7 |
| 7 | Figure 7. HREIMS spectrum of Patriscabioin M (**1**) | 8 |
| 8 | Figure 8. ^1^H NMR spectrum of Patriscabioin N (**2**) in CD_3_OD | 9 |
| 9 | Figure 9. ^13^C NMR spectrum of Patriscabioin N (**2**) in CD_3_OD | 9 |
| 10 | Figure 10. HSQC spectrum of Patriscabioin N (**2**) in CD_3_OD | 10 |
| 11 | Figure 11. HMBC spectrum of Patriscabioin N (**2**) in CD_3_OD | 10 |
| 12 | Figure 12. ^1^H-^1^H COSY spectrum of Patriscabioin N (**2**) in CD_3_OD | 11 |
| 13 | Figure 13. ROESY spectrum of Patriscabioin N (**2**) in CD_3_OD | 11 |
| 14 | Figure 14. HREIMS spectrum of Patriscabioin N (**2**) | 12 |
| 15 | Figure 15. ^1^H NMR spectrum of Patriscabioin O (**3**) in C_5_D_5_N | 13 |
| 16 | Figure 16. ^13^C NMR spectrum of Patriscabioin O (**3**) in C_5_D_5_N | 13 |
| 17 | Figure 17. HSQC spectrum of Patriscabioin O (**3**) in C_5_D_5_N | 14 |
| 18 | Figure 18. HMBC spectrum of Patriscabioin O (**3**) in C_5_D_5_N | 14 |
| 19 | Figure 19. ^1^H-^1^H COSY spectrum of Patriscabioin O (**3**) in C_5_D_5_N | 15 |
| 20 | Figure 20. ROESY spectrum of Patriscabioin O (**3**) in C_5_D_5_N | 15 |
| 21 | Figure 21. HREIMS spectrum of Patriscabioin O (**3**) | 16 |
| 22 | Figure 22. ^1^H NMR spectrum of Patrinoside B (**5**) in CD_3_OD | 17 |
| 23 | Figure 23. ^13^C NMR spectrum of Patrinoside B (**5**) in CD_3_OD | 17 |
| 24 | Figure 24. HSQC spectrum of Patrinoside B (**5**) in CD_3_OD | 18 |
| 25 | Figure 25. HMBC spectrum of Patrinoside B (**5**) in CD_3_OD | 18 |
| 26 | Figure 26. ^1^H-^1^H COSY spectrum of Patrinoside B (**5**) in CD_3_OD | 19 |
| 27 | Figure 27. ROESY spectrum of Patrinoside B (**5**) in CD_3_OD | 19 |
| 28 | Figure 28. HREIMS spectrum of Patrinoside B (**5**) | 20 |
| 29 | Figure 29. ^1^H NMR spectrum of Patrinoside C (**6**) in CD_3_OD | 21 |
| 30 | Figure 30. ^13^C NMR spectrum of Patrinoside C (**6**) in CD_3_OD | 21 |
| 31 | Figure 31. HSQC spectrum of Patrinoside C (**6**) in CD_3_OD | 22 |
| 32 | Figure 32. HMBC spectrum of Patrinoside C (**6**) in CD_3_OD | 22 |
| 33 | Figure 33. ^1^H-^1^H COSY spectrum of Patrinoside C (**6**) in CD_3_OD | 23 |
| 34 | Figure 34. ROESY spectrum of Patrinoside C (**6**) in CD_3_OD | 23 |
| 35 | Figure 35. HREIMS spectrum of Patrinoside C (**6**) | 24 |
| 36 | Figure 36. ^1^H NMR spectrum of Patrinoside D (**7**) in CD_3_OD | 25 |
| 37 | Figure 37. ^13^C NMR spectrum of Patrinoside D (**7**) in CD_3_OD | 25 |
| 38 | Figure 38. HSQC spectrum of Patrinoside D (**7**) in CD_3_OD | 26 |
| 39 | Figure 39. HMBC spectrum of Patrinoside D (**7**) in CD_3_OD | 26 |
| 40 | Figure 40. ^1^H-^1^H COSY spectrum of Patrinoside D (**7**) in CD_3_OD | 27 |
| 41 | Figure 41. ROESY spectrum of Patrinoside D (**7**) in CD_3_OD | 27 |
| 42 | Figure 42. HREIMS spectrum of Patrinoside D (**7**) | 28 |
| 43 | Figure 43. ^1^H NMR spectrum of Patrinoside E (**8**) in C_5_D_5_N | 29 |
| 44 | Figure 44. ^13^C NMR spectrum of Patrinoside E (**8**) in C_5_D_5_N | 29 |
| 45 | Figure 45. HSQC spectrum of Patrinoside E (**8**) in C_5_D_5_N | 30 |
| 46 | Figure 46. HMBC spectrum of Patrinoside E (**8**) in C_5_D_5_N | 30 |
| 47 | Figure 47. ^1^H-^1^H COSY spectrum of Patrinoside E (**8**) in C_5_D_5_N | 31 |
| 48 | Figure 48. ROESY spectrum of Patrinoside E (**8**) in C_5_D_5_N | 31 |
| 49 | Figure 49. HREIMS spectrum of Patrinoside E (**8**) | 32 |
| 50 | Figure 50. ^1^H NMR spectrum of Patrinoside F (**9**) in CD_3_OD | 33 |
| 51 | Figure 51. ^13^C NMR spectrum of Patrinoside F (**9**) in CD_3_OD | 33 |
| 52 | Figure 52. HSQC spectrum of Patrinoside F (**9**) in CD_3_OD | 34 |
| 53 | Figure 53. HMBC spectrum of Patrinoside F (**9**) in CD_3_OD | 34 |
| 54 | Figure 54. ^1^H-^1^H COSY spectrum of Patrinoside F (**9**) in CD_3_OD | 35 |
| 55 | Figure 55. ROESY spectrum of Patrinoside F (**9**) in CD_3_OD | 35 |
| 56 | Figure 56. HREIMS spectrum of Patrinoside F (**9**) | 36 |
| 57 | Figure 57. ^1^H NMR spectrum of Patriscabiobisin D (**10**) in CD_3_OD | 37 |
| 58 | Figure 58.^13^C NMR spectrum of Patriscabiobisin D (**10**) in CD_3_OD | 37 |
| 59 | Figure 59. HSQC spectrum of Patriscabiobisin D (**10**) in CD_3_OD | 38 |
| 60 | Figure 60. HMBC spectrum of Patriscabiobisin D (**10**) in CD_3_OD | 38 |
| 61 | Figure 61. ^1^H-^1^H COSY spectrum of Patriscabiobisin D (**10**) in CD_3_OD | 39 |
| 62 | Figure 62. ROESY spectrum of Patriscabiobisin D (**10**) in CD_3_OD | 39 |
| 63 | Figure 63. HREIMS spectrum of Patriscabiobisin D (**10**) | 40 |

Figure 1 ^1^H NMR (Bruker AM-800, 800 MHz, CD_3_OD) of Patrirscabioin M (**1**)


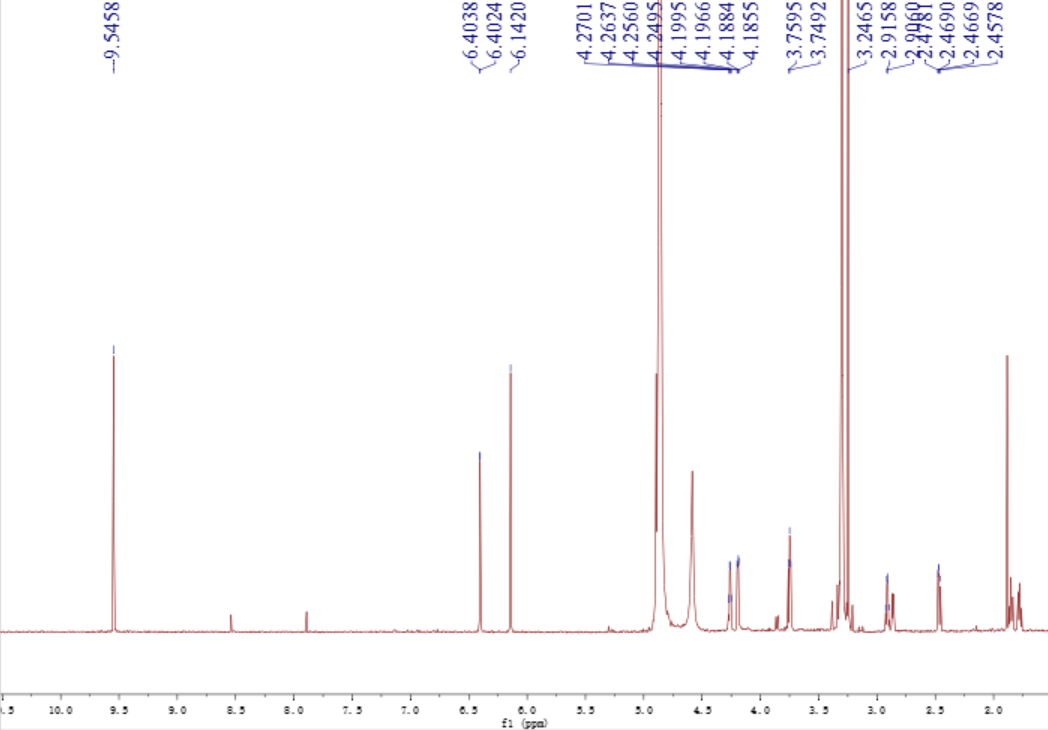


Figure 2 ^13^C NMR (Bruker AM-800, 200 MHz, CD_3_OD) of Patrirscabioin M (**1**)


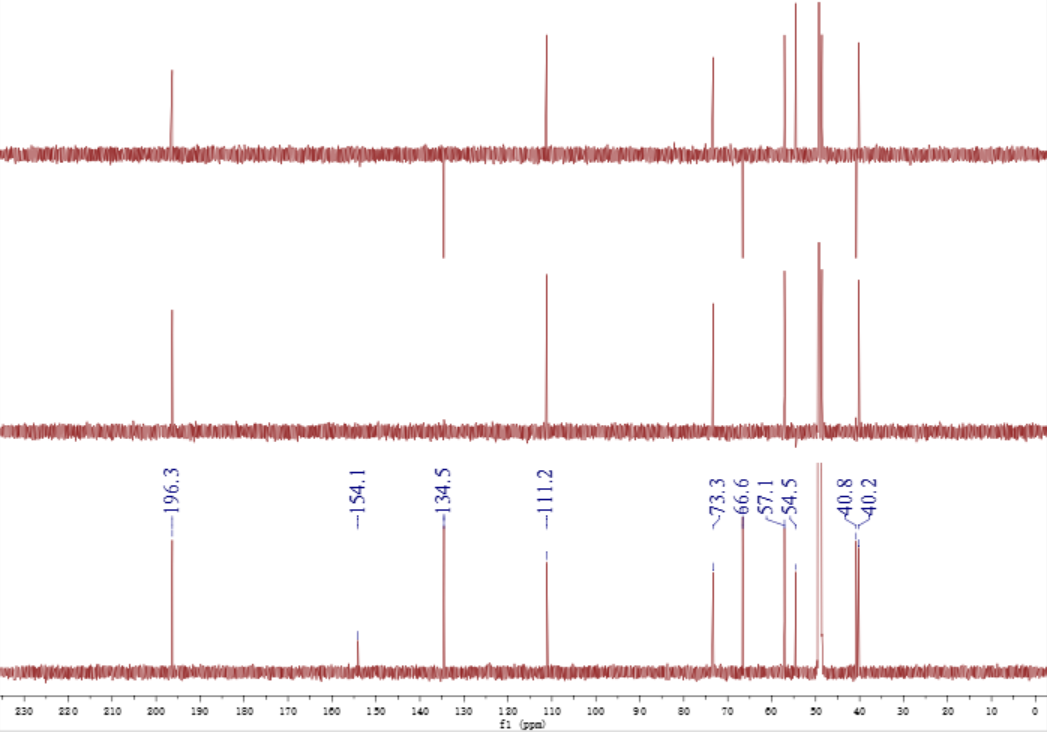


Figure 3 HSQC (Bruker DRX-800, 800 MHz, 200 MHz, CD_3_OD) of Patrirscabioin M (**1**)


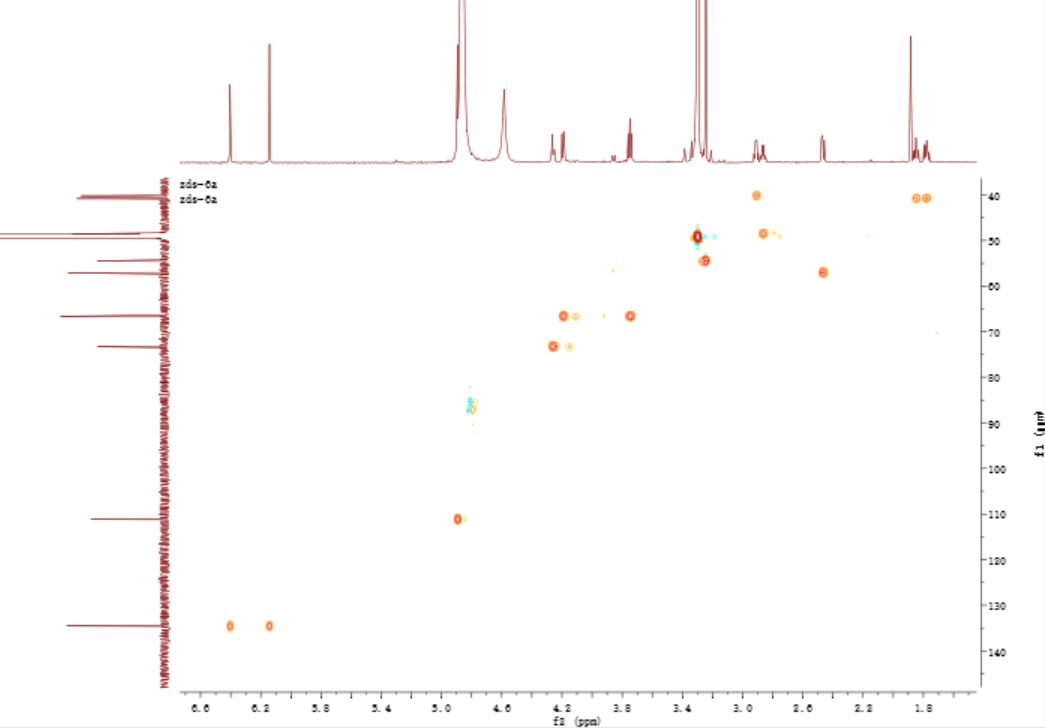


Figure 4 HMBC (Bruker DRX-800, 800 MHz, 200 MHz, CD_3_OD) of Patrirscabioin M (**1**)


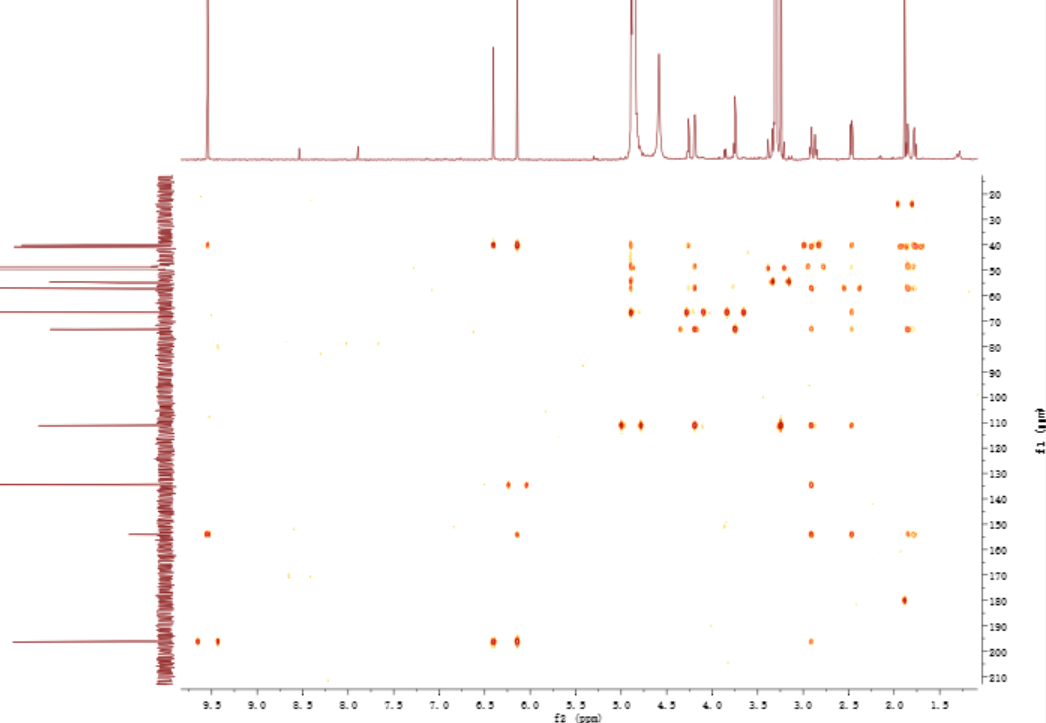


Figure 5 ^1^H-^1^H COSY (Bruker DRX-800, 800 MHz, 800 MHz, CD_3_OD) of Patrirscabioin M (**1**)


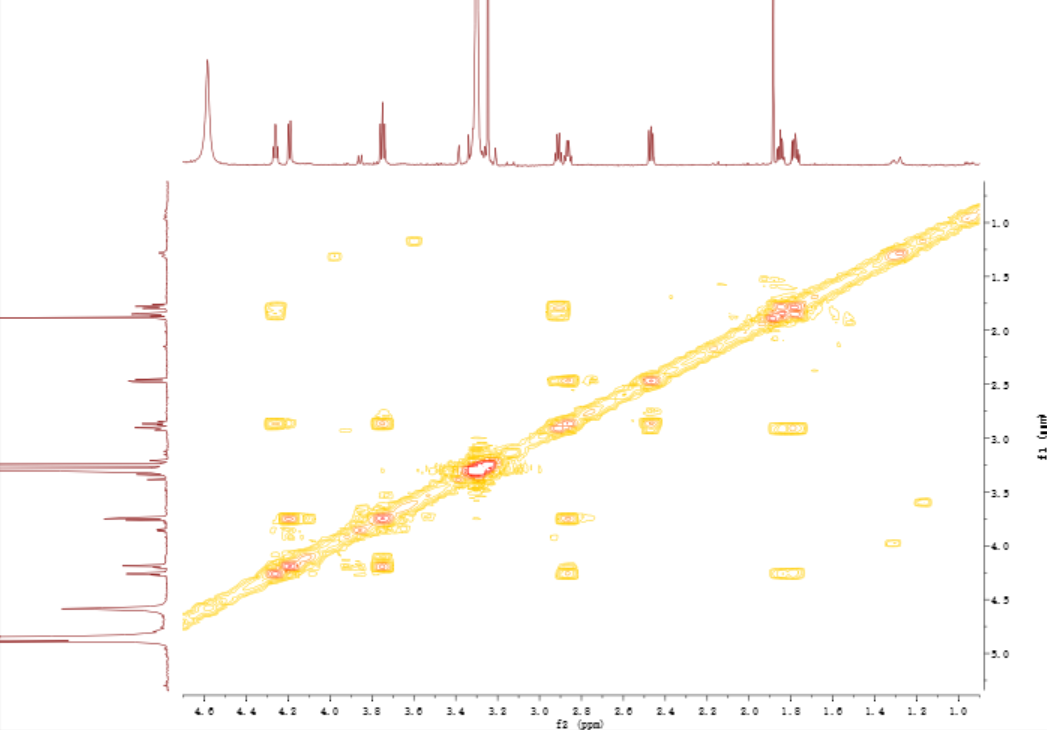


Figure 6 ROESY (Bruker DRX-800, 800 MHz, 800 MHz, CD_3_OD) of Patrirscabioin M (**1**)


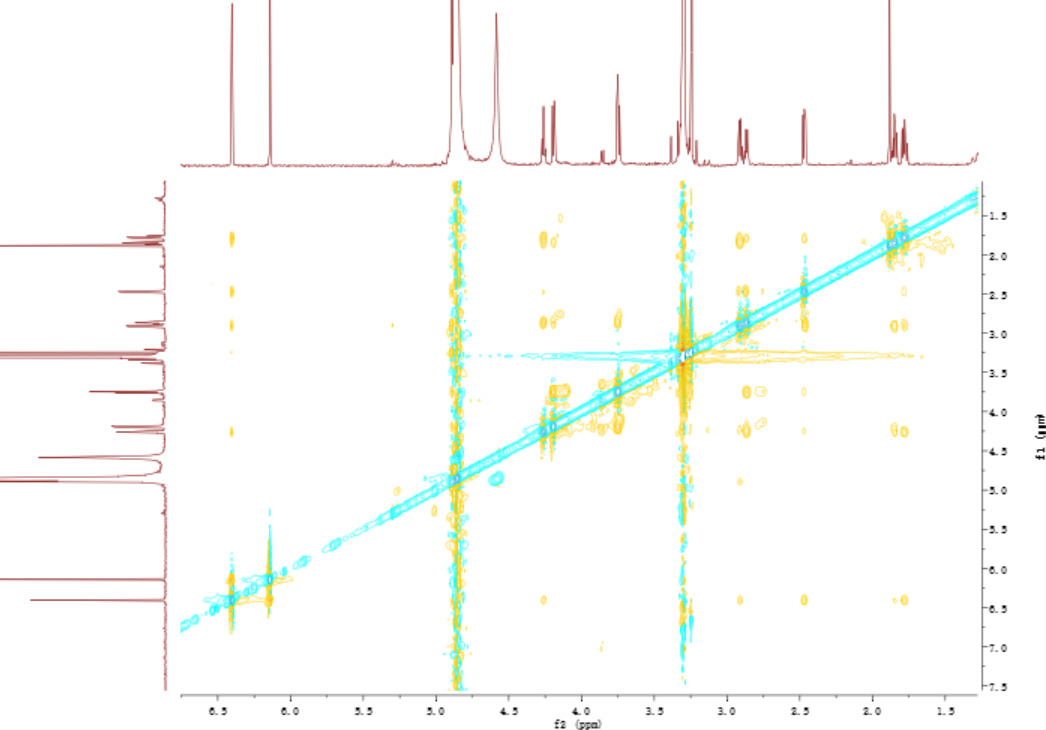


Figure 7HREIMS spectrum of Patriscabioin A (**1**)

Figure 8 ^1^H NMR (Bruker AM-500, 500 MHz, CD_3_OD) of Patrirscabioin N (**2**)


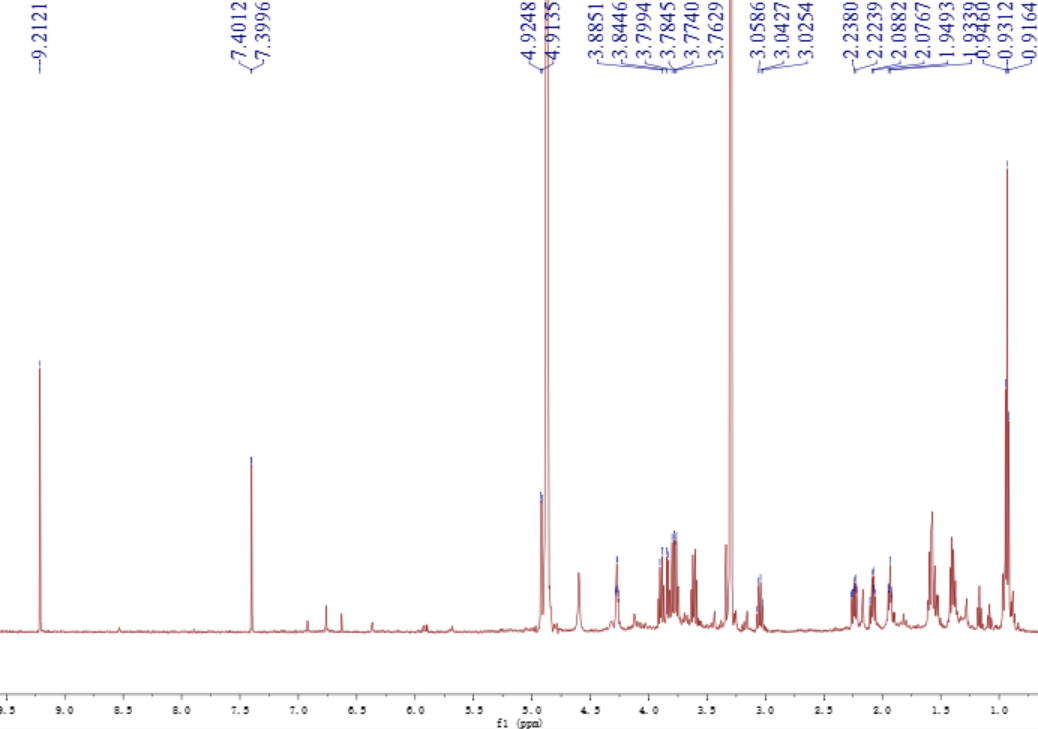


Figure 9 ^13^C NMR (Bruker AM-500, 125 MHz, CD_3_OD) of Patrirscabioin N (**2**)


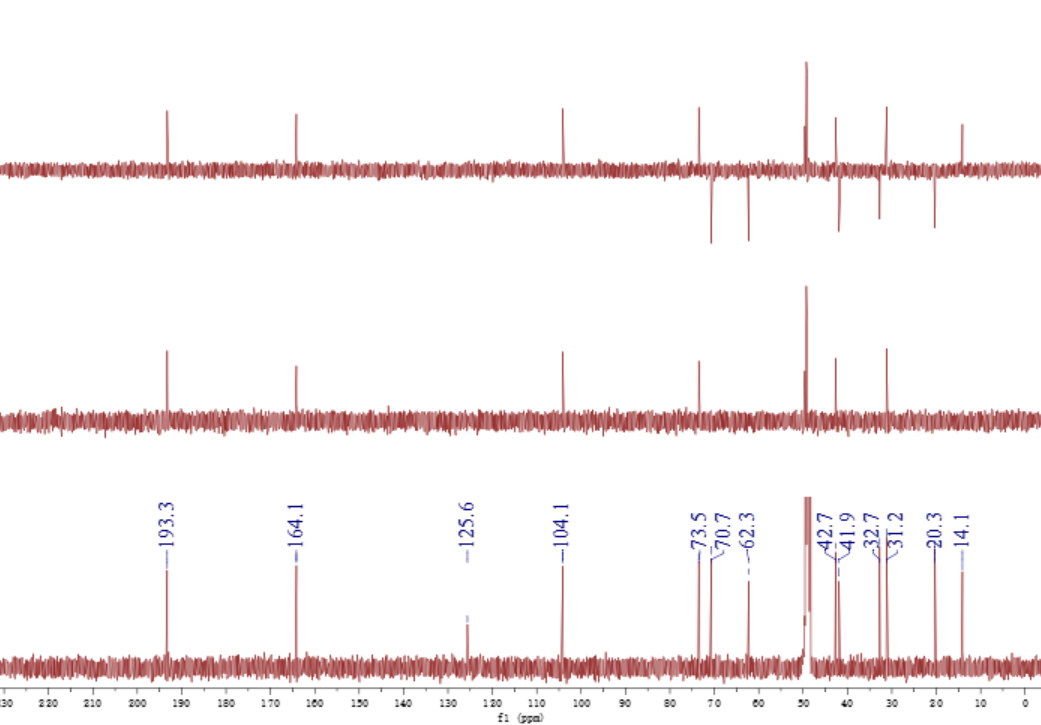


Figure 10 HSQC (Bruker DRX-500, 500 MHz, 125 MHz, CD_3_OD) of PatrirscabioinN (**2**)


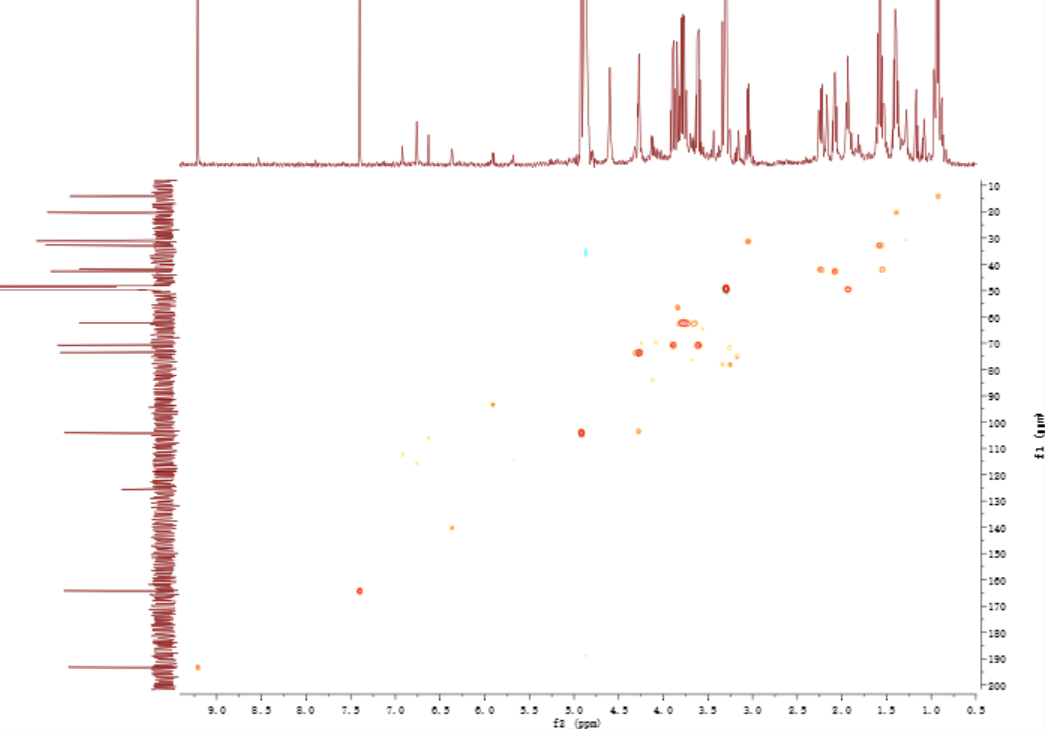


Figure 11 HMBC (Bruker DRX-500, 500 MHz, 125 MHz, CD_3_OD) of PatrirscabioinN (**2**)


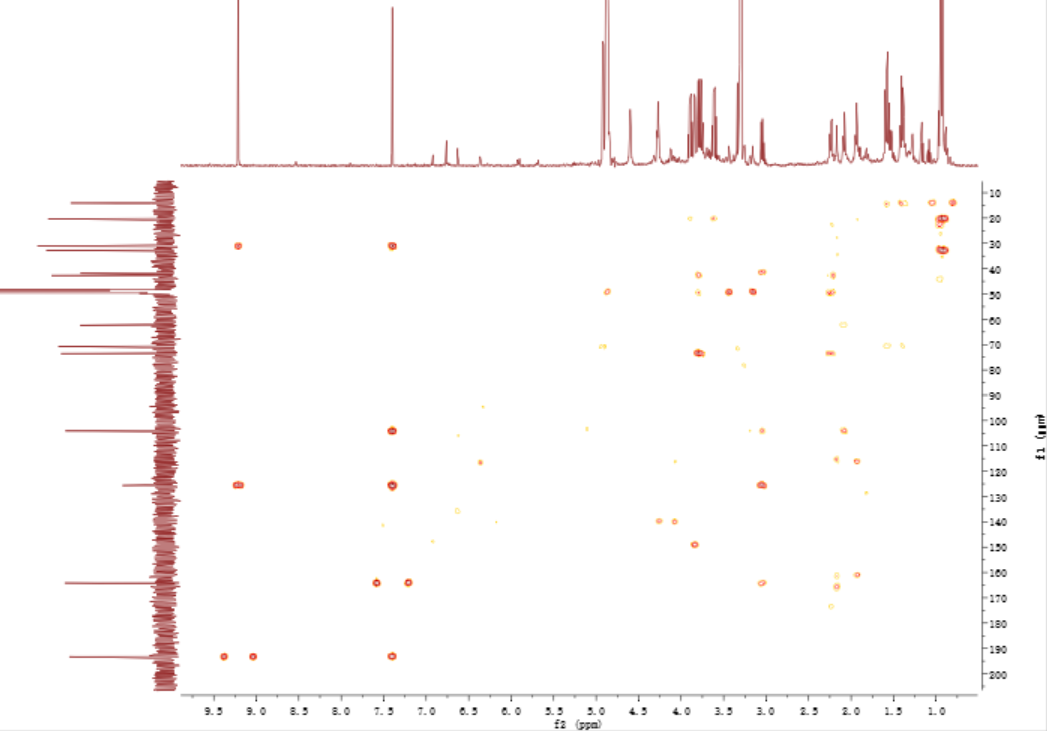


Figure 12 ^1^H-^1^H COSY (Bruker DRX-500, 500 MHz, 500 MHz, CD_3_OD) of PatrirscabioinN (**2**)


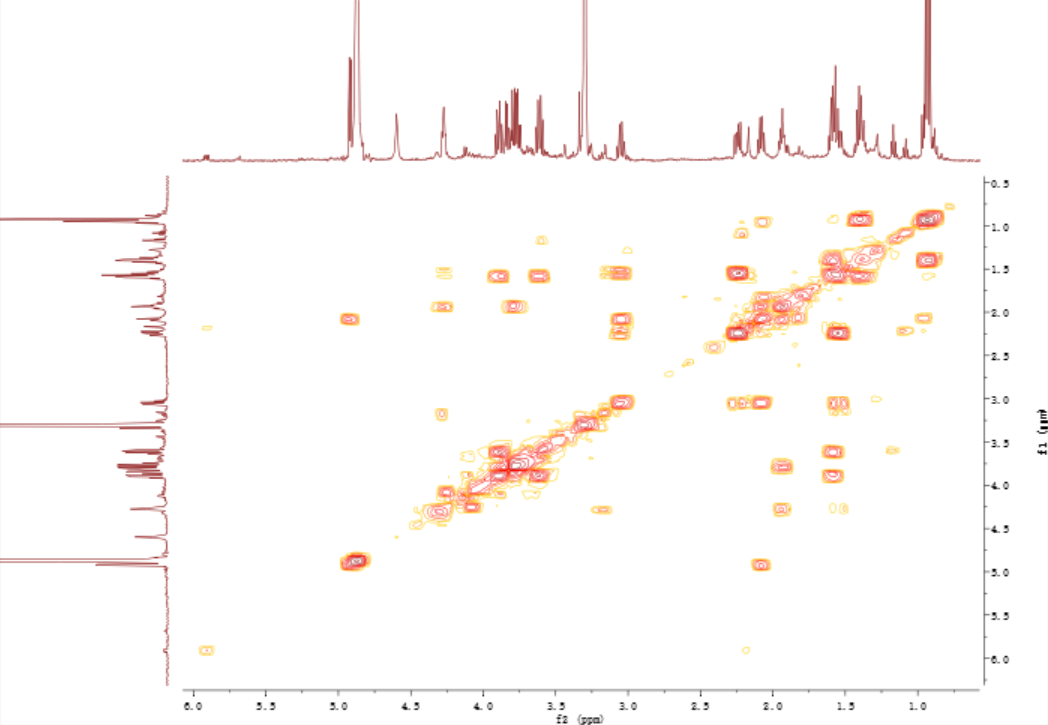


Figure 13 ROESY (Bruker DRX-500, 500 MHz, 500 MHz, CD_3_OD) of PatrirscabioinN (**2**)


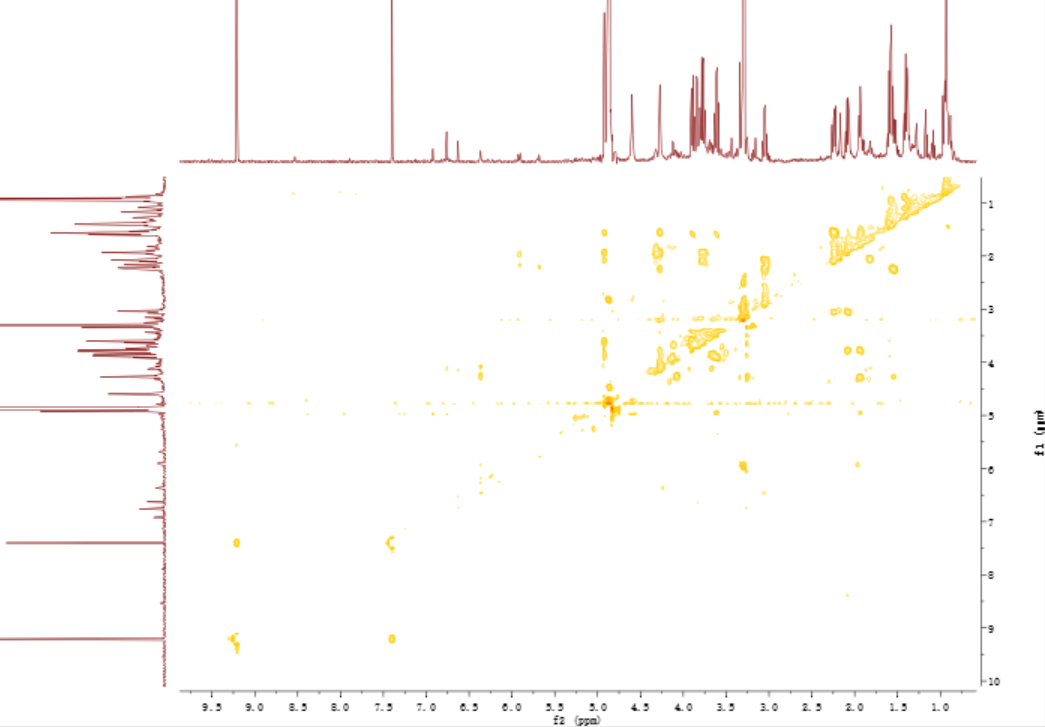


Figure 14HREIMS spectrum of PatrirscabioinN (**2**)

Figure 15^1^H NMR (Bruker AM-500, 500 MHz, C_5_D_5_N) of PatrirscabioinO (**3**)


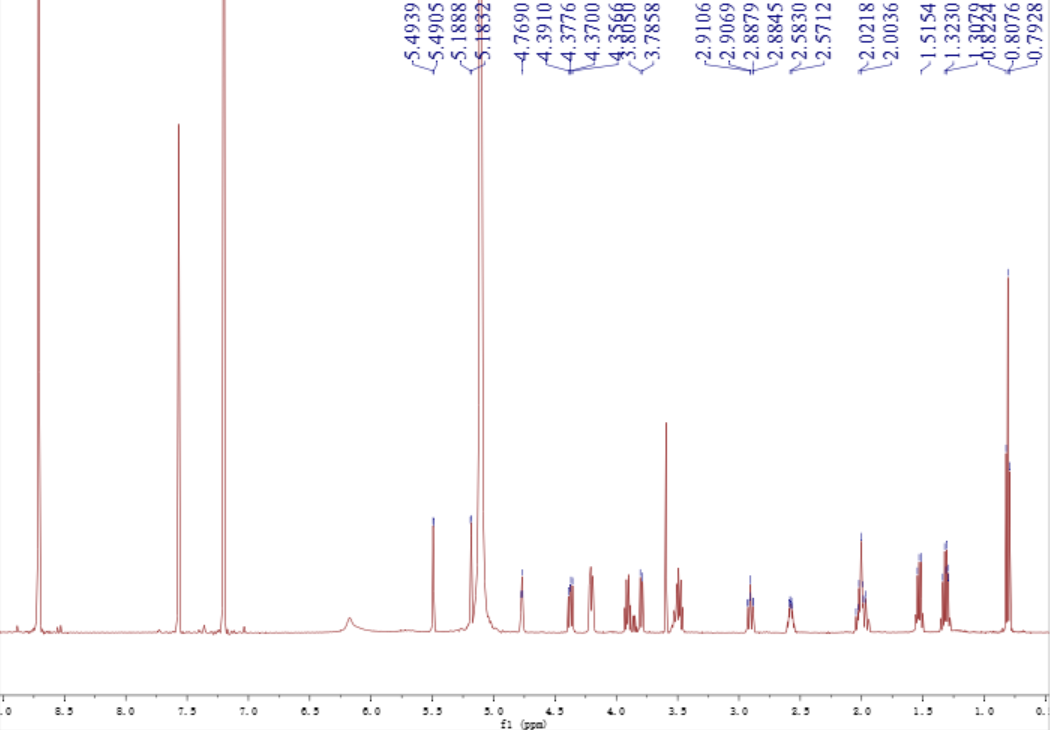


Figure 16 ^13^C NMR (Bruker AM-500, 125 MHz, C_5_D_5_N) of PatrirscabioinO (**3**)


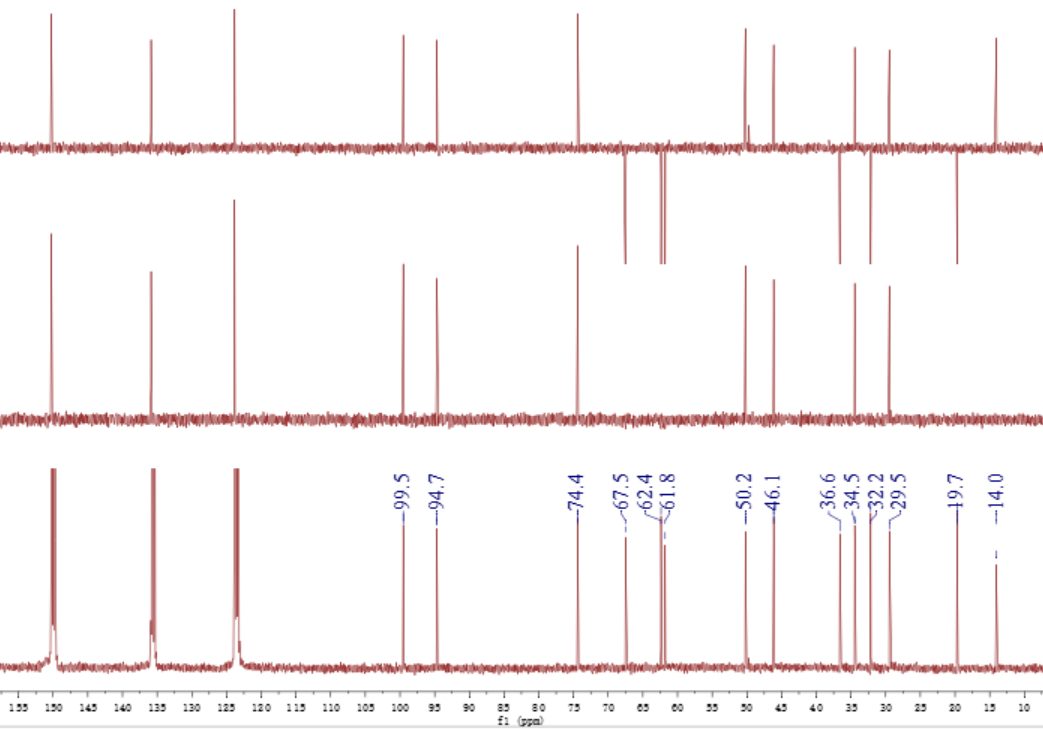


Figure 17 HSQC (Bruker DRX-500, 500 MHz, 125 MHz, C_5_D_5_N) of PatrirscabioinO (**3**)


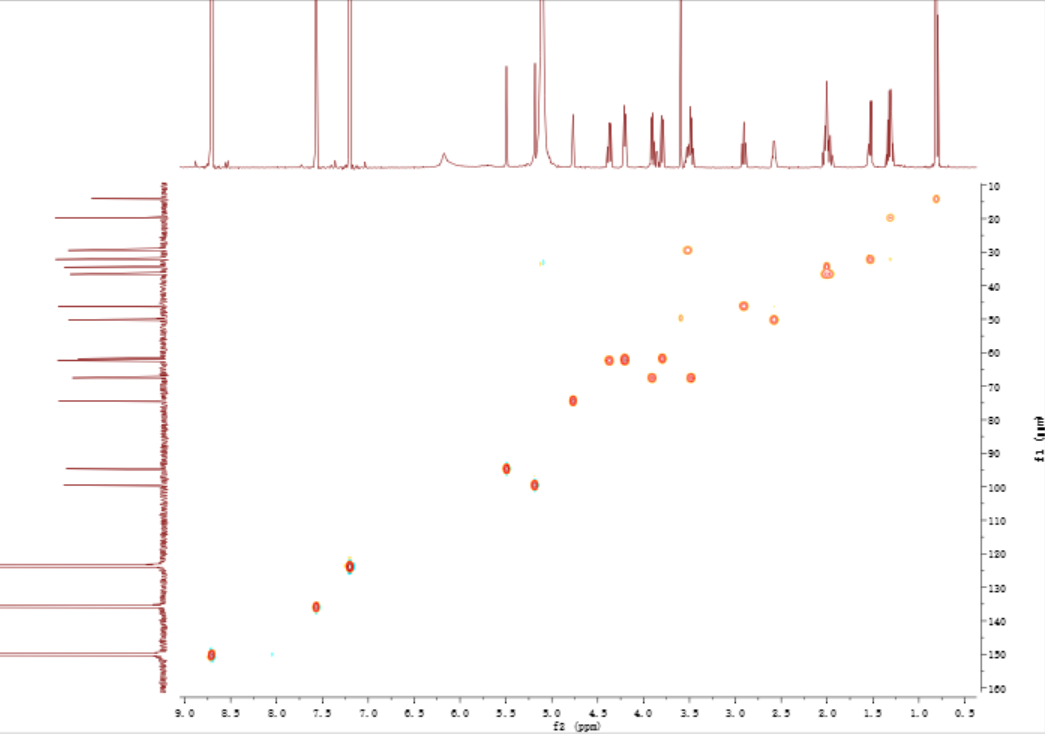


Figure 18 HMBC (Bruker DRX-500, 500 MHz, 125 MHz, C_5_D_5_N) of PatrirscabioinO (**3**)


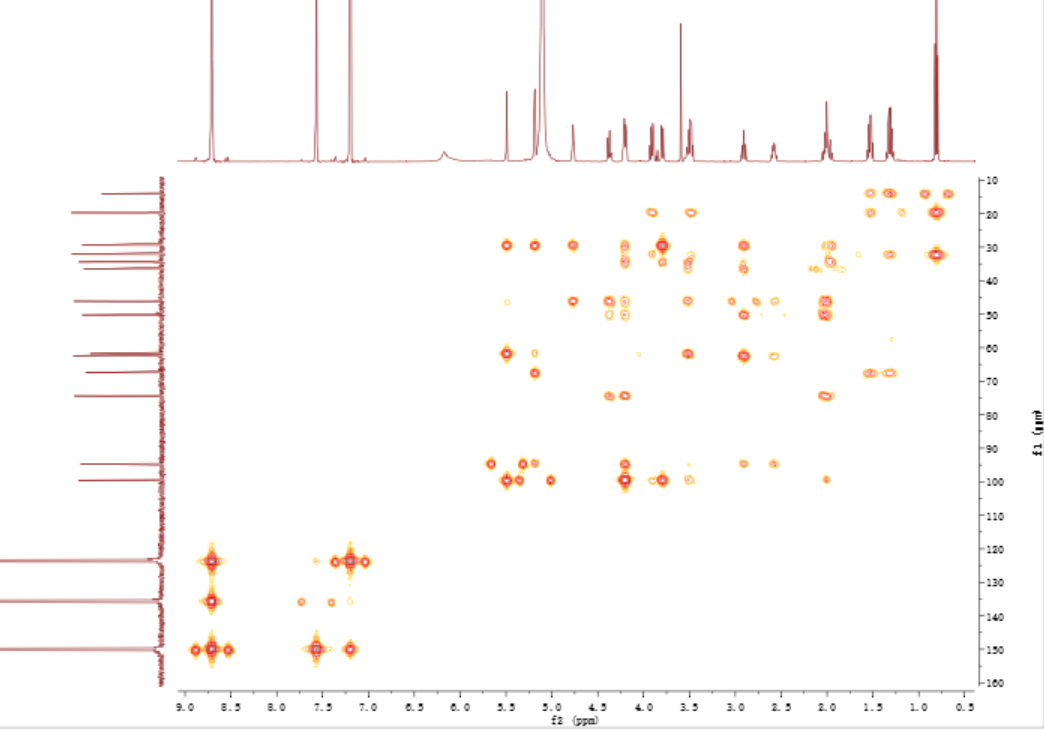


Figure 19 ^1^H-^1^H COSY (Bruker DRX-500, 500 MHz, 500 MHz, C_5_D_5_N) of PatrirscabioinO (**3**)


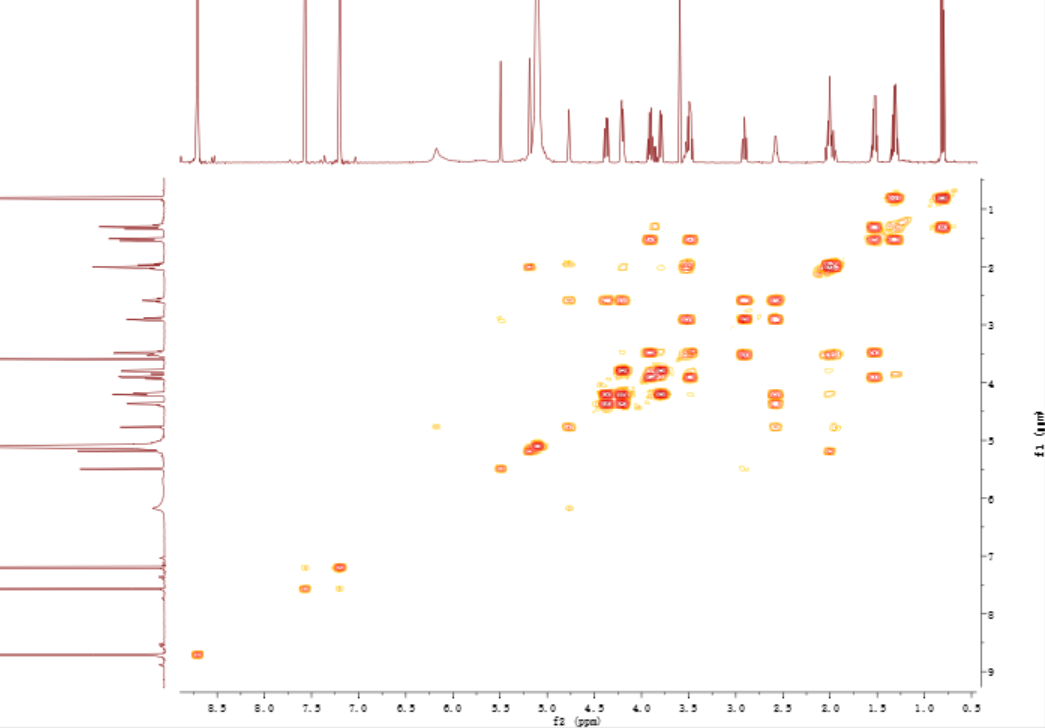


Figure 20 ROESY (Bruker DRX-500, 500 MHz, 500 MHz, C_5_D_5_N) of PatrirscabioinO (**3**)


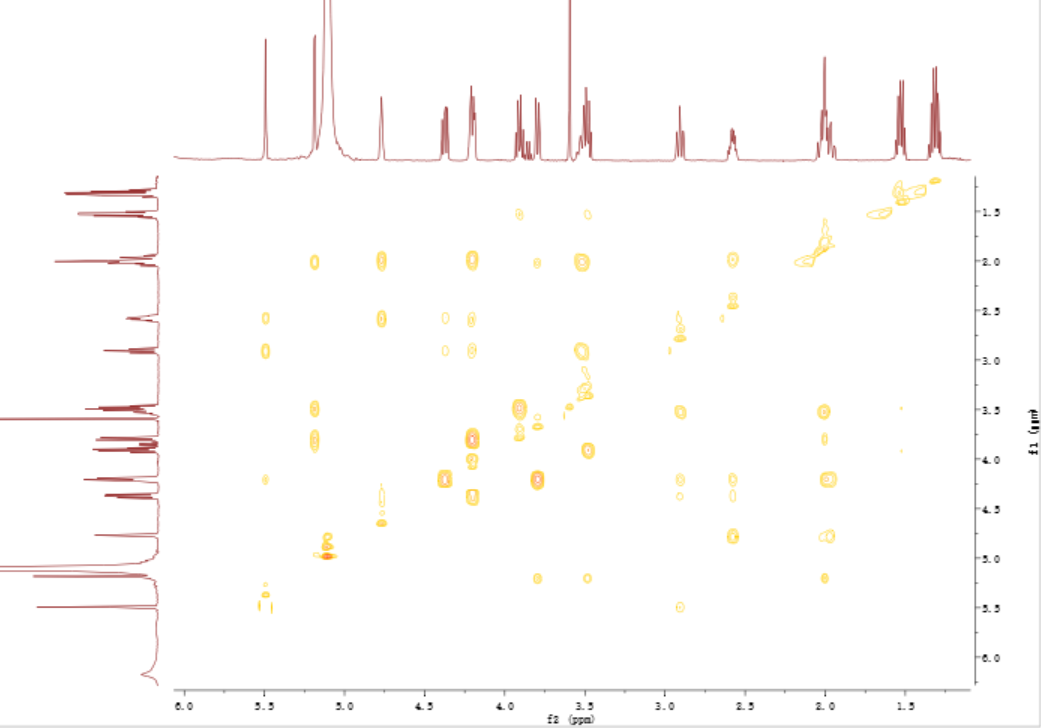


Figure 21 HREIMS spectrum of PatrirscabioinO (**3**)

Figure 22 ^1^H NMR (Bruker AM-800, 800 MHz, CD_3_OD) of Patrinoside B (**5**)


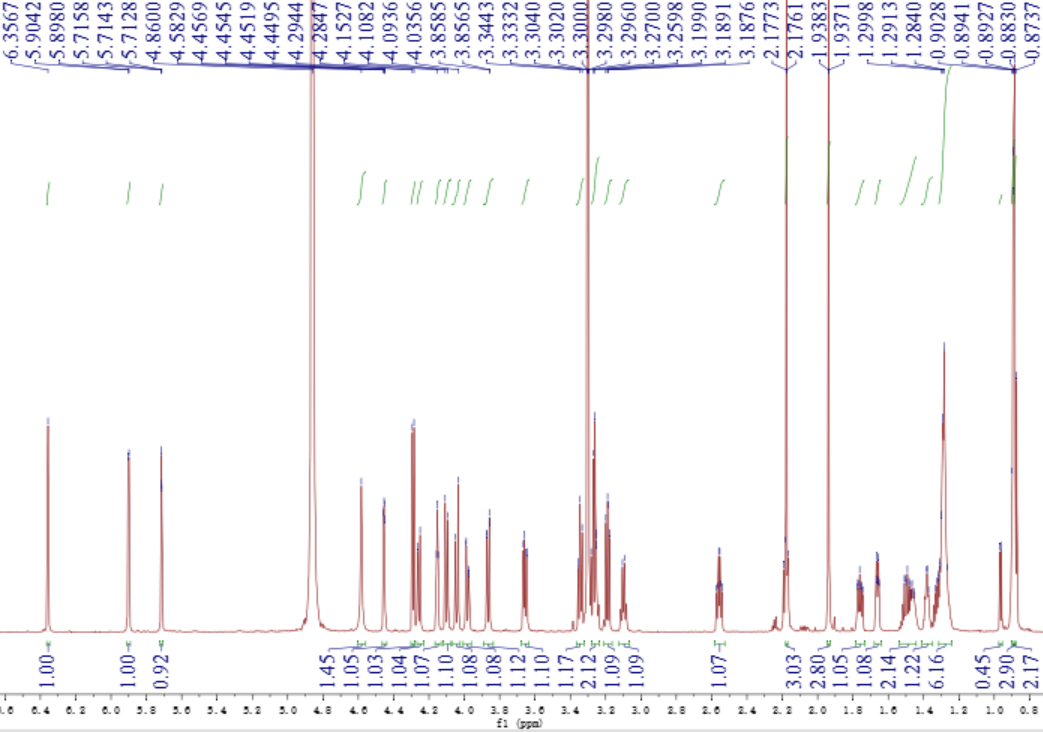


Figure 23 ^13^C NMR (Bruker AM-800, 200 MHz, CD_3_OD) of Patrinoside B (**5**)


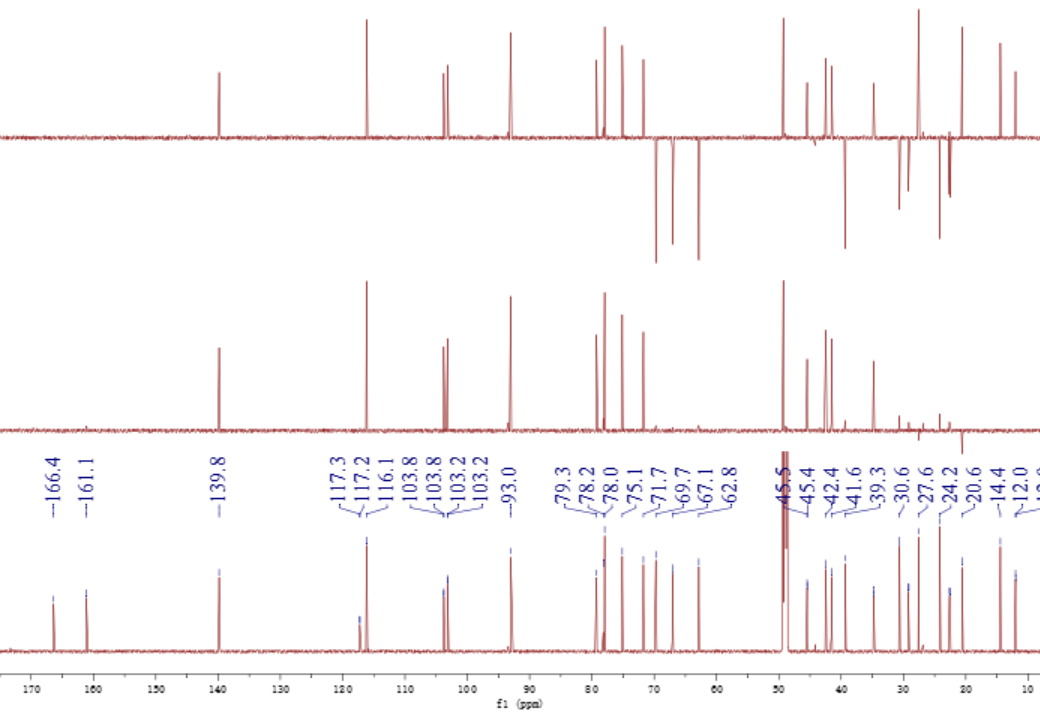


Figure 24 HSQC (Bruker DRX-800, 800 MHz, 200 MHz, CD_3_OD) of Patrinoside B (**5**)


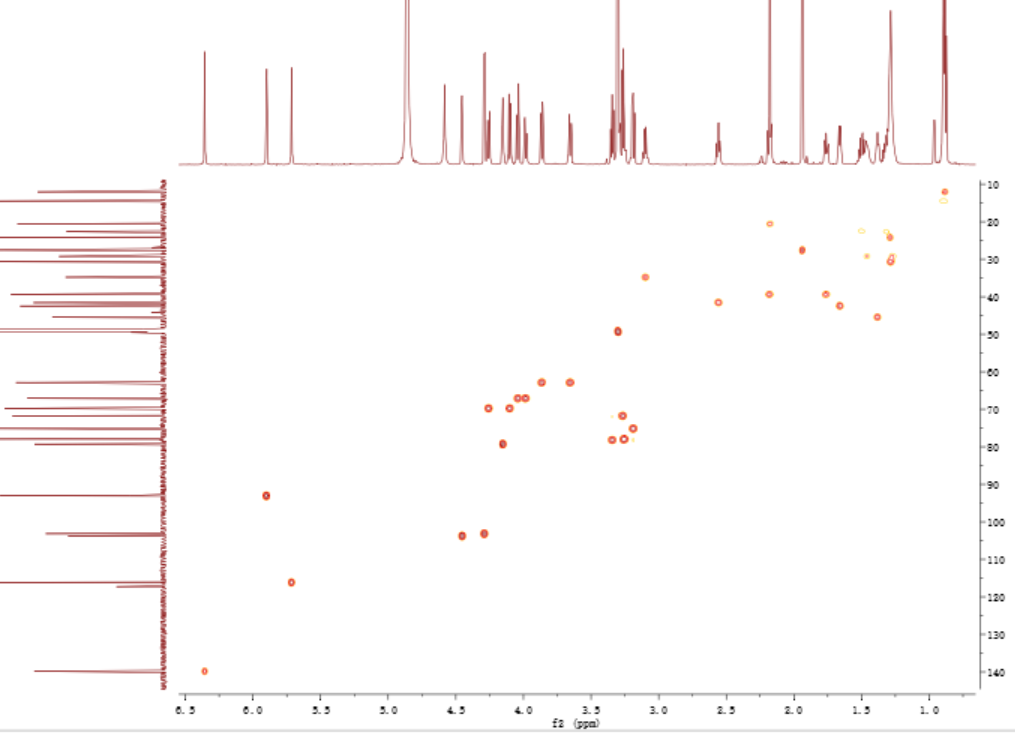


Figure 25 HMBC (Bruker DRX-800, 800 MHz, 200 MHz, CD_3_OD) of Patrinoside B (**5**)


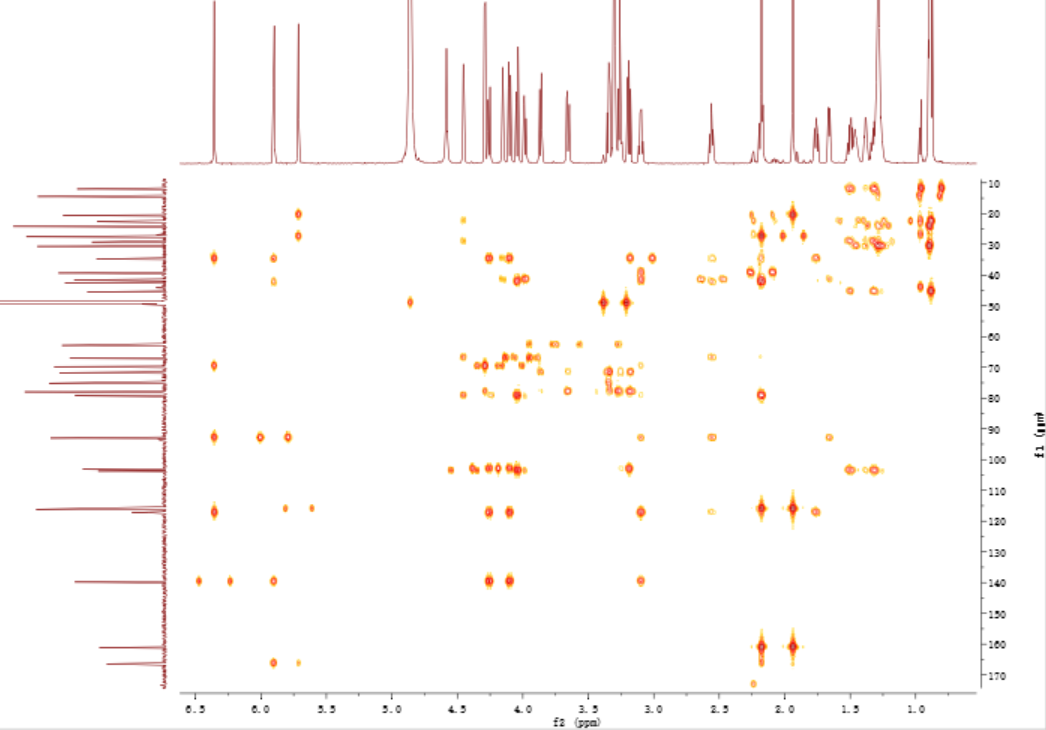


Figure 26 ^1^H-^1^H COSY(Bruker DRX-800, 800 MHz, 800 MHz,CD_3_OD) of Patrinoside B (**5**)


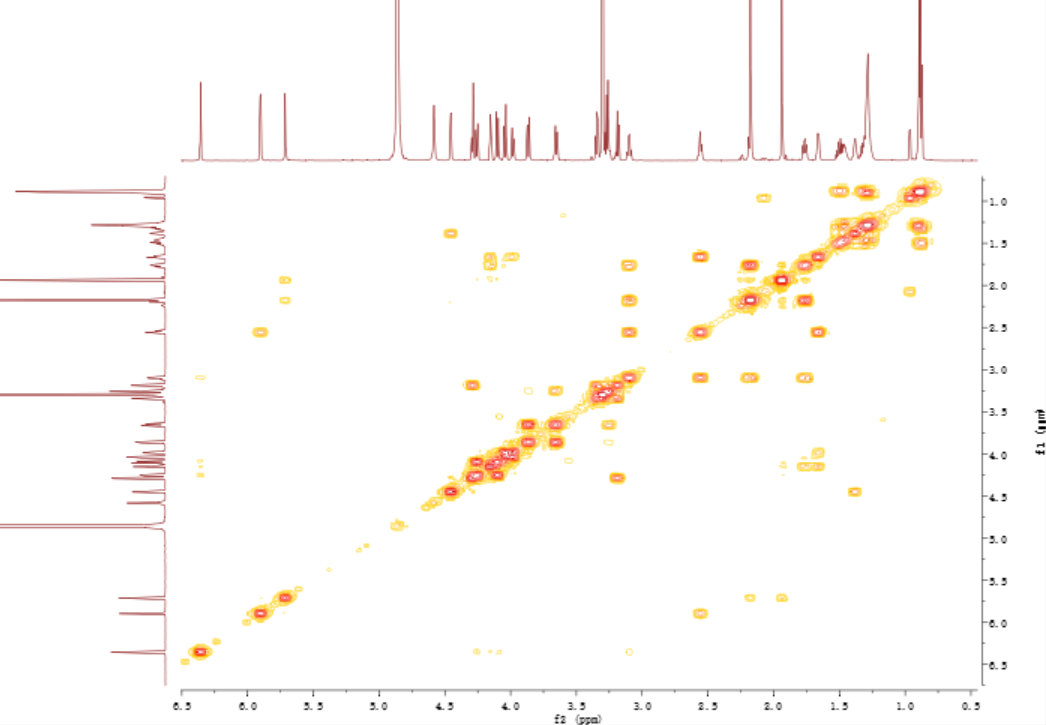


Figure 27 ROESY (Bruker DRX-800, 800 MHz, 800 MHz, CD_3_OD) of Patrinoside B (**5**)


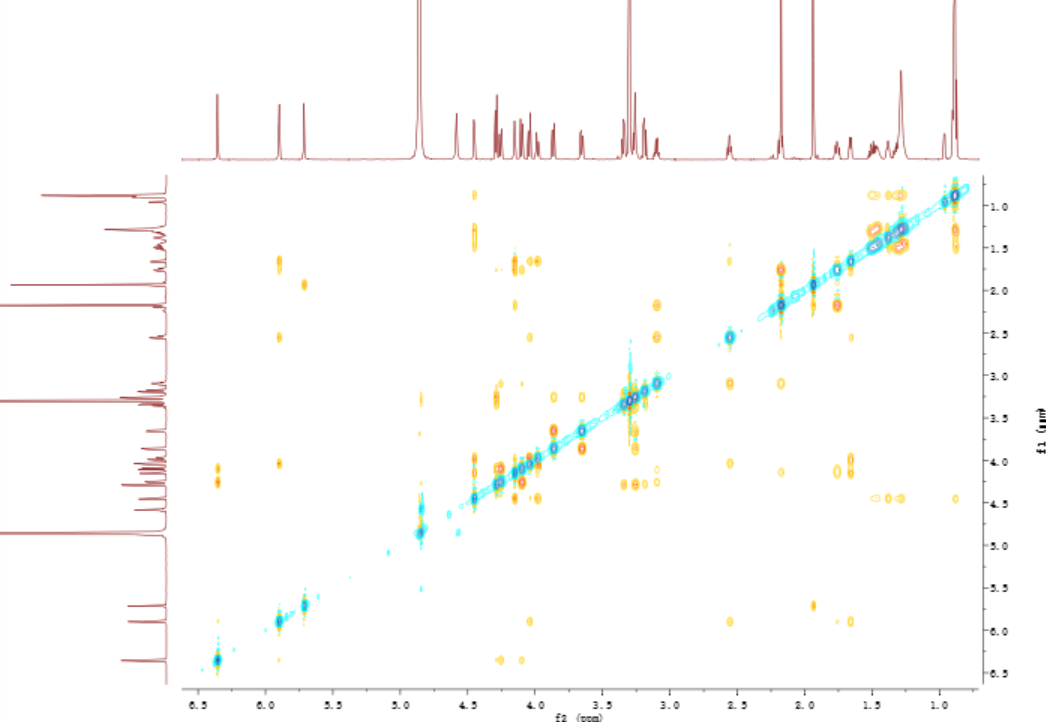


Figure 28 HREIMS spectrum of Patrinoside B (**5**)

Figure 29 ^1^H NMR (Bruker AM-500, 500 MHz, CD_3_OD) of Patrinoside C (**6**)


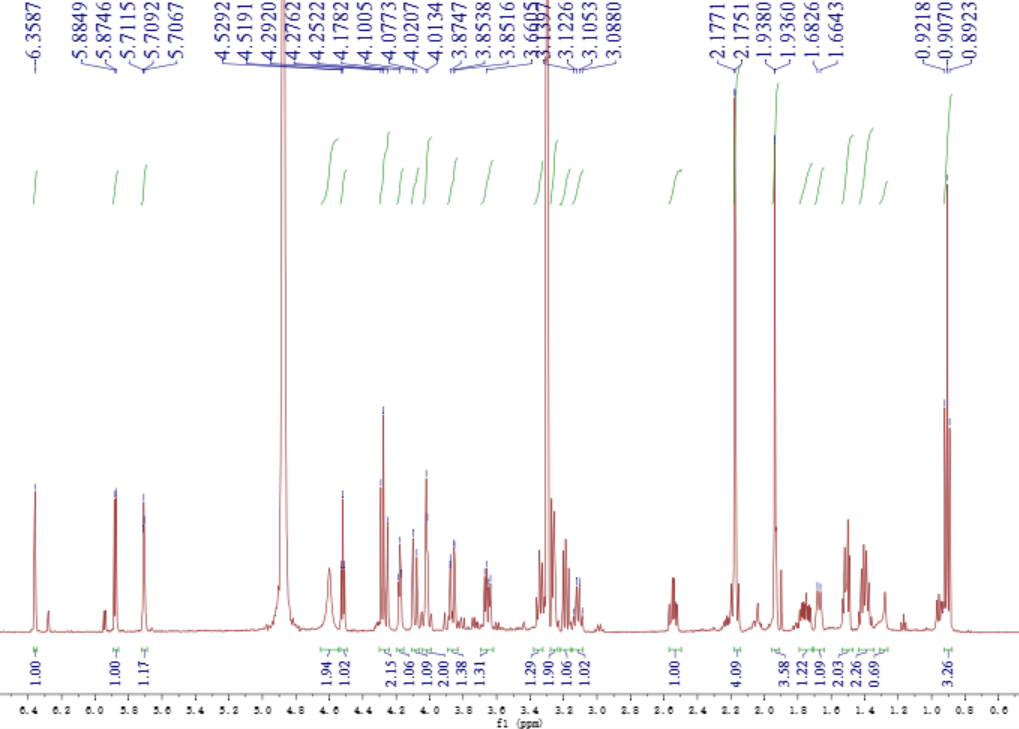


Figure 30 ^13^C NMR (Bruker AM-500, 125 MHz, CD_3_OD) of Patrinoside C (**6**)


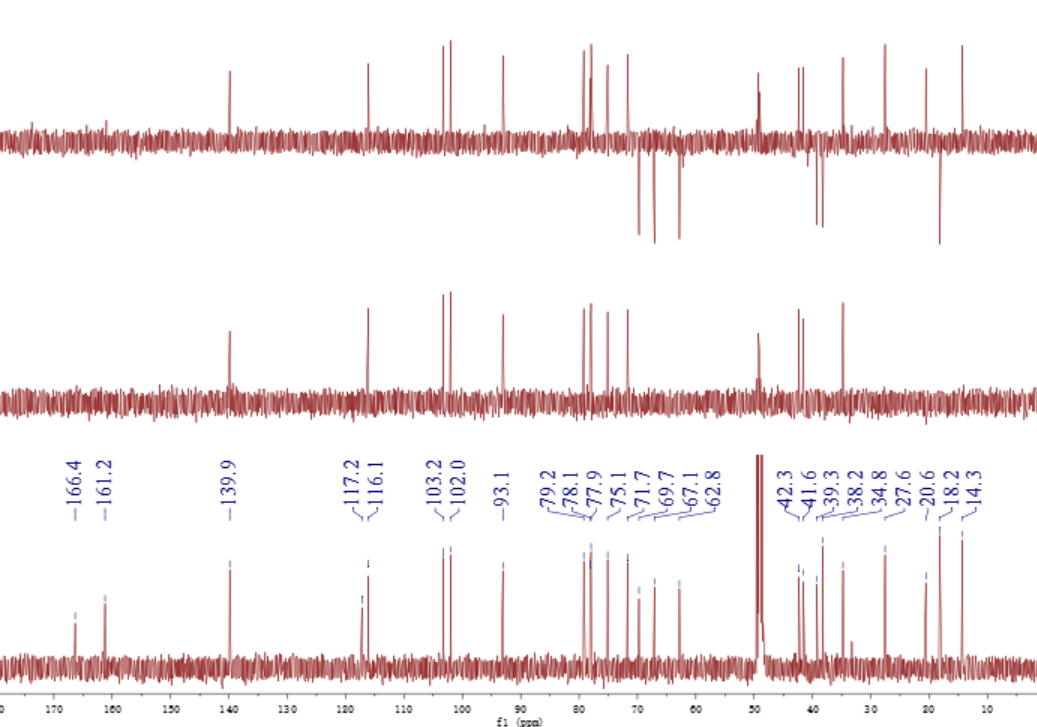


Figure 31 HSQC (Bruker DRX-500, 500 MHz, 125 MHz, CD_3_OD) of Patrinoside C (**6**)


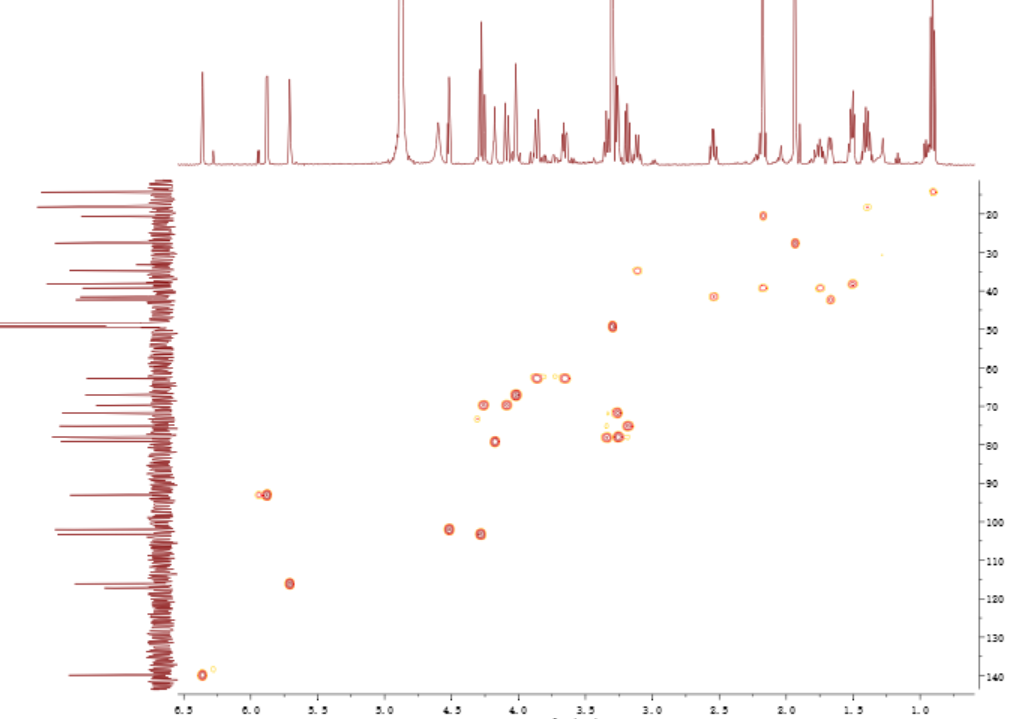


Figure 32 HMBC (Bruker DRX-500, 500 MHz, 125 MHz, CD_3_OD) of Patrinoside C (**6**)


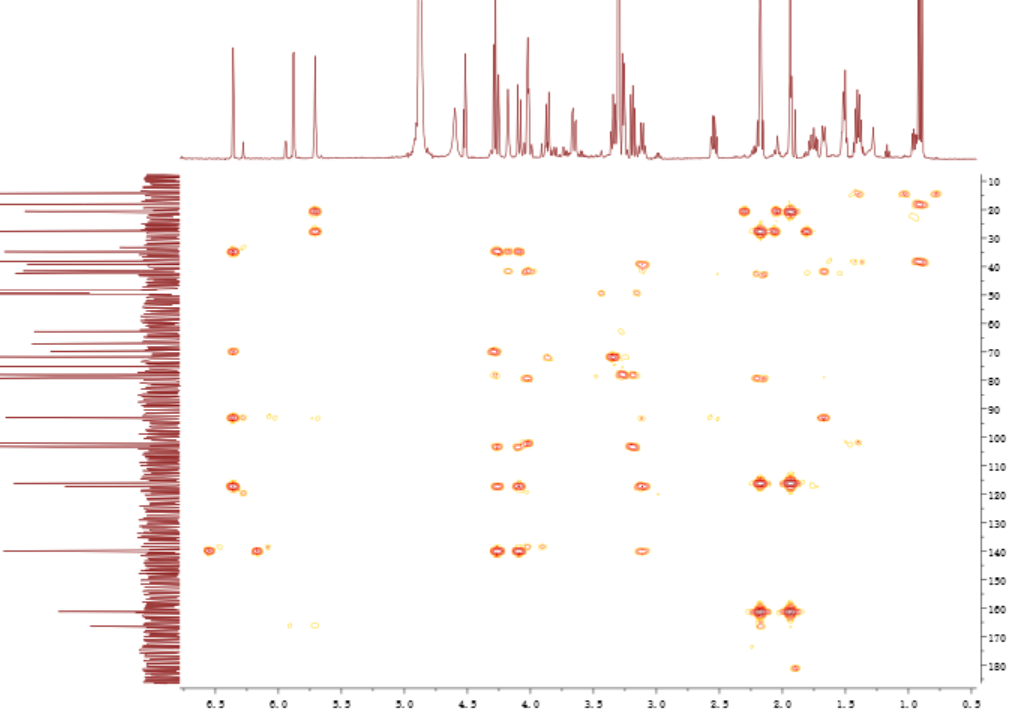


Figure 33 ^1^H-^1^H COSY (Bruker DRX-500, 500 MHz, 500 MHz, CD_3_OD) of Patrinoside C (**6**)


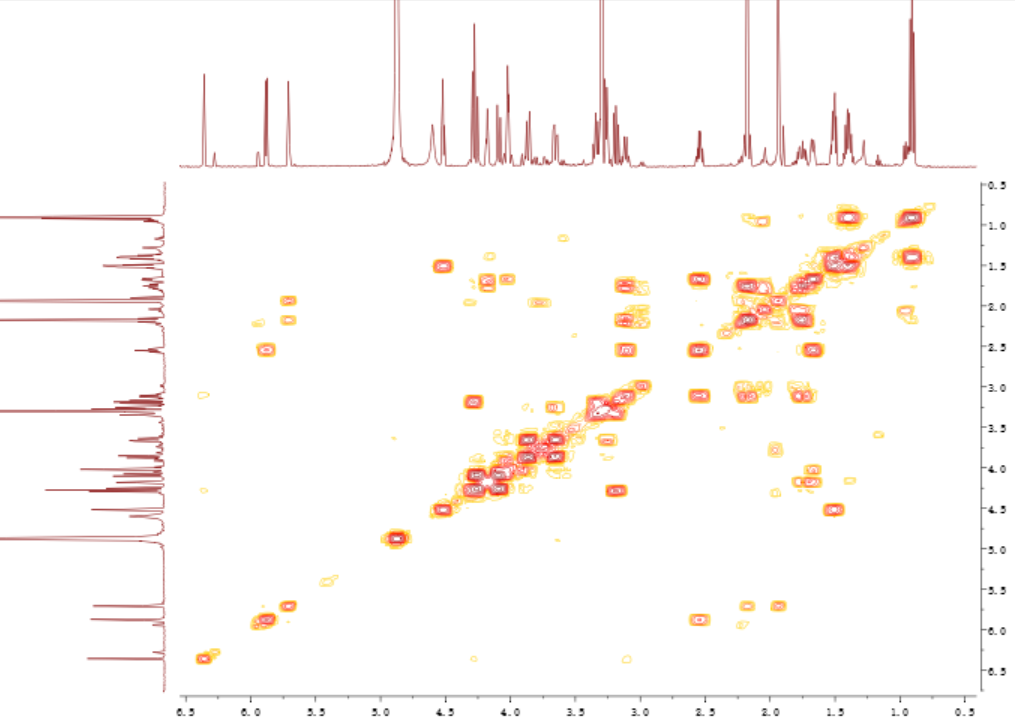


Figure 34 ROESY (Bruker DRX-500, 500 MHz, 500 MHz, CD_3_OD) of Patrinoside C (**6**)


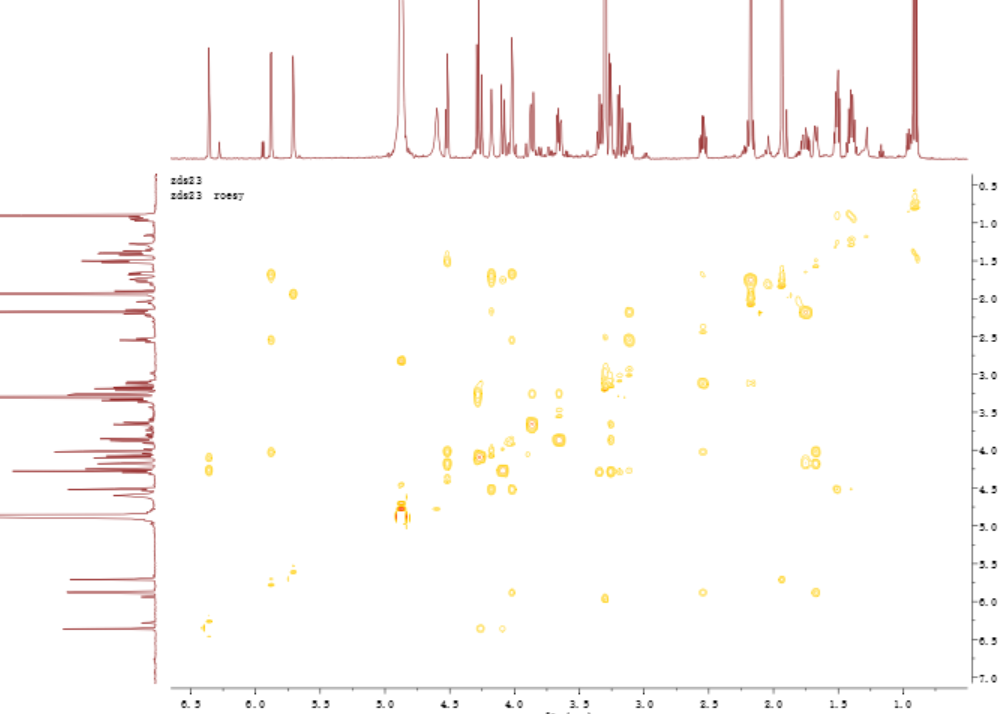


Figure 35 HREIMS spectrum of Patrinoside C (**6**)

Figure 36 ^1^H NMR (Bruker AM-800, 800 MHz, CD_3_OD) of Patrinoside D (**7**)


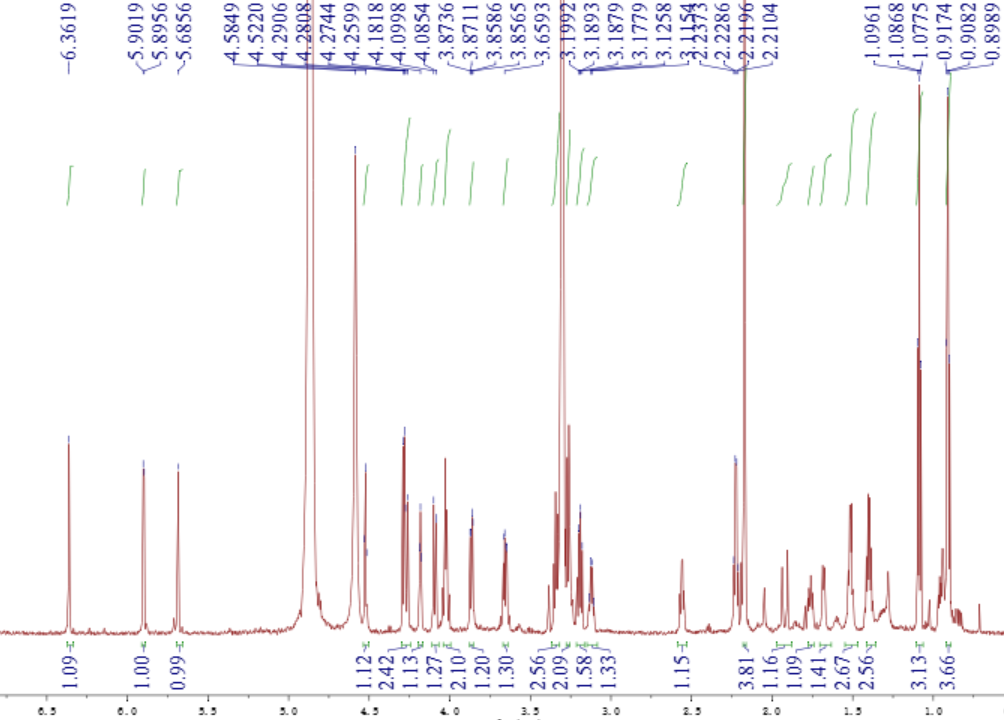


Figure 37 ^13^C NMR (Bruker AM-800, 200 MHz, CD_3_OD) of Patrinoside D (**7**)


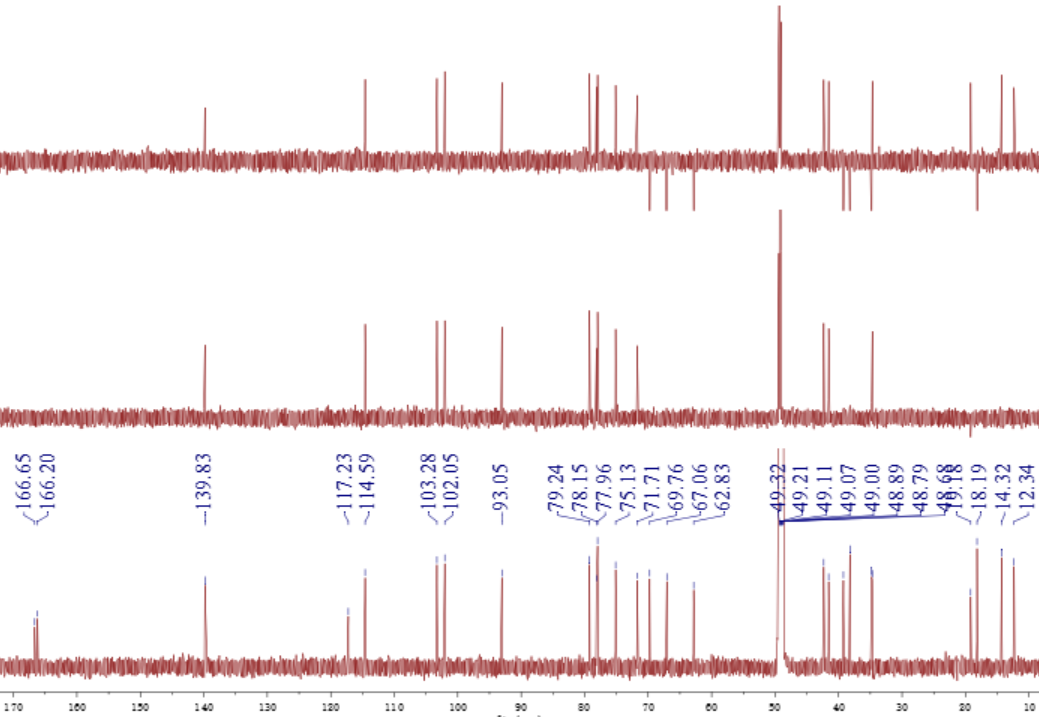


Figure 38 HSQC (Bruker DRX-800, 800 MHz, 200 MHz, CD_3_OD) of Patrinoside D (**7**)


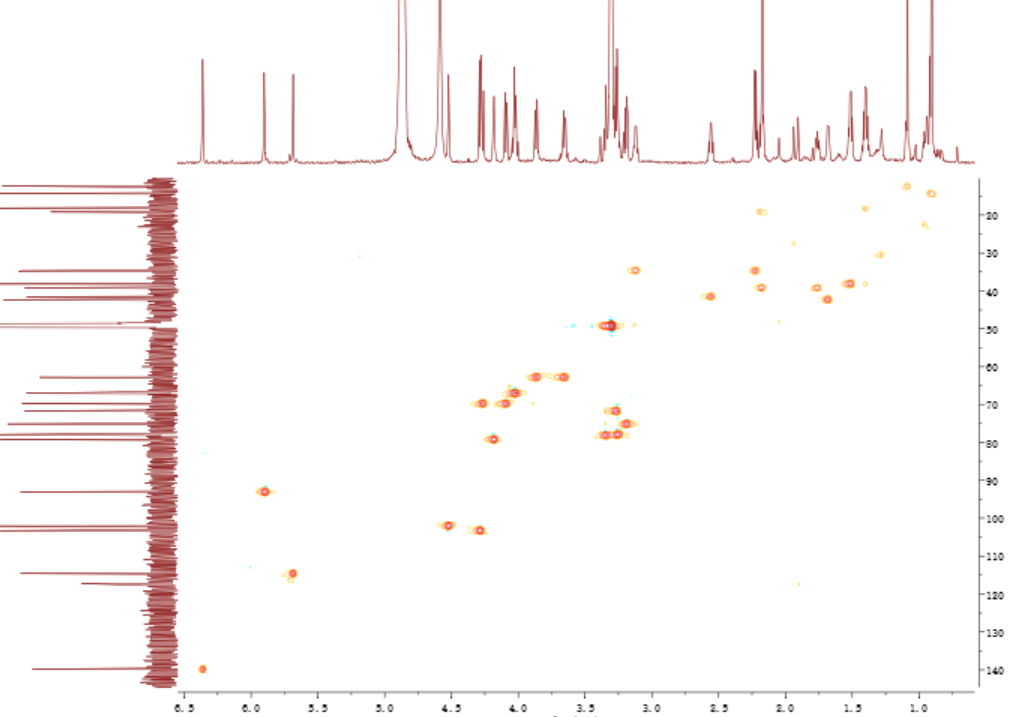


Figure 39 HMBC (Bruker DRX-800, 800 MHz, 200 MHz, CD_3_OD) of Patrinoside D (**7**)


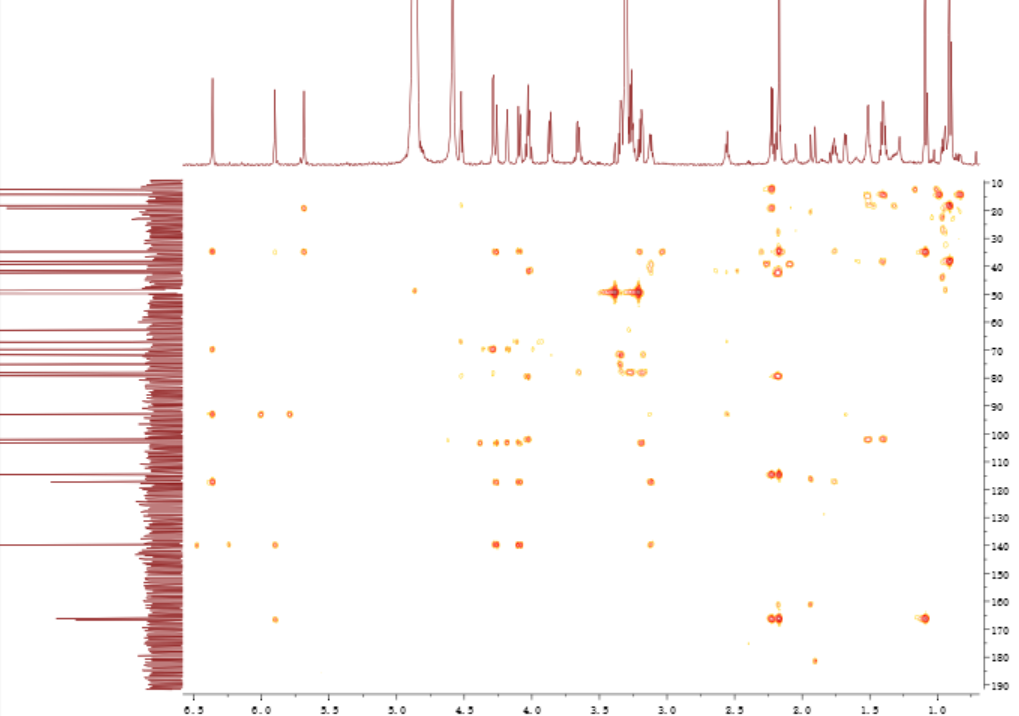


Figure 40 ^1^H-^1^H COSY (Bruker DRX-800, 800 MHz, 800 MHz, CD_3_OD) of Patrinoside D (**7**)


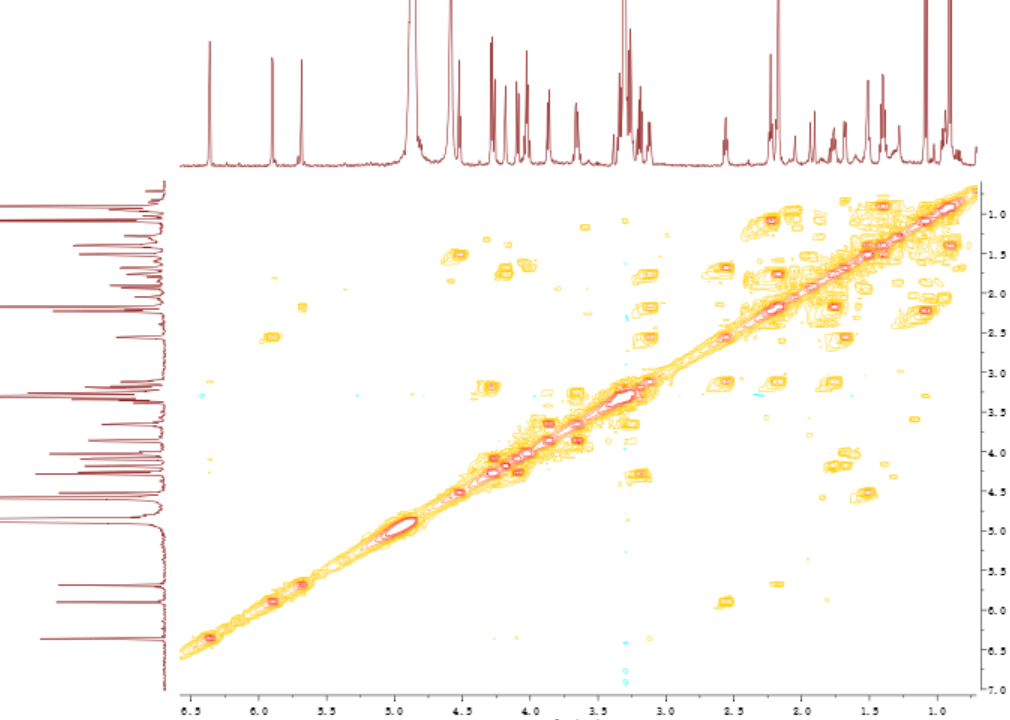


Figure 41 ROESY (Bruker DRX-800, 800 MHz, 800 MHz, CD_3_OD) of Patrinoside D (**7**)


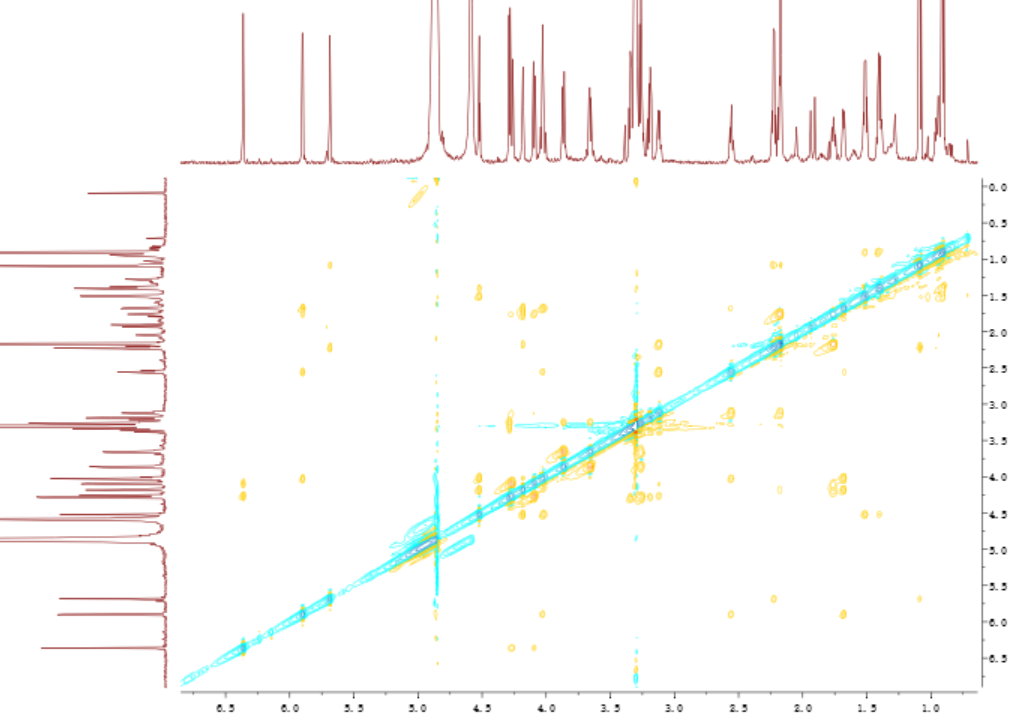


Figure 42 HREIMS spectrum of Patrinoside D (**7**)

Figure 43 ^1^H NMR (Bruker AM-500, 500 MHz, C_5_D_5_N) of Patrinoside E (**8**)


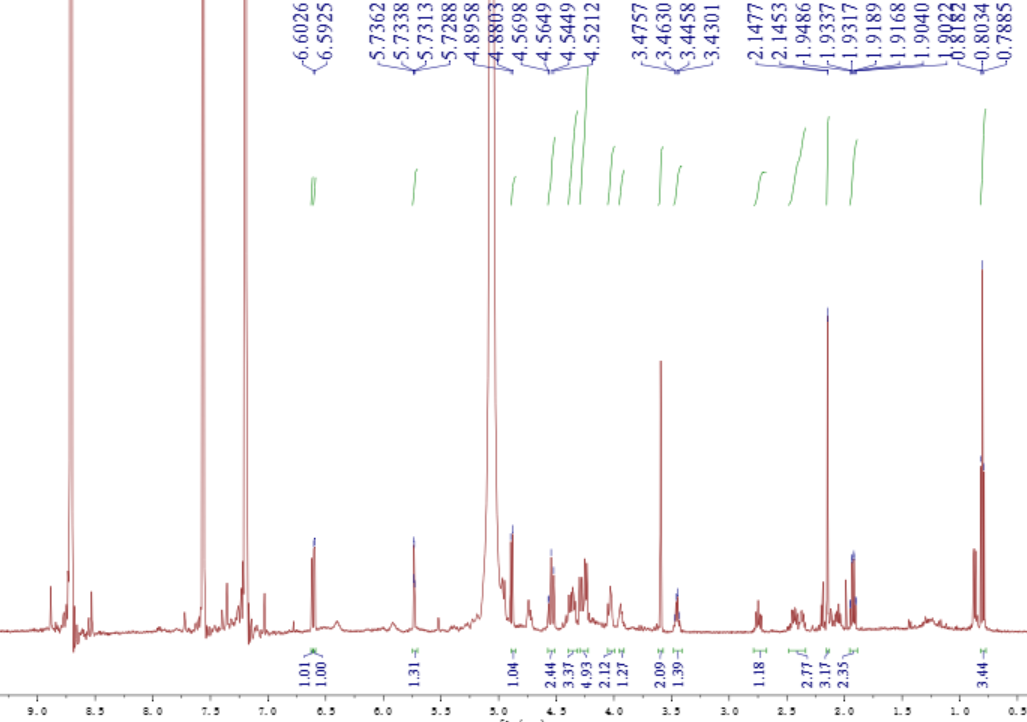


Figure 44 ^13^C NMR (Bruker AM-500, 125 MHz, C_5_D_5_N) of Patrinoside E (**8**)


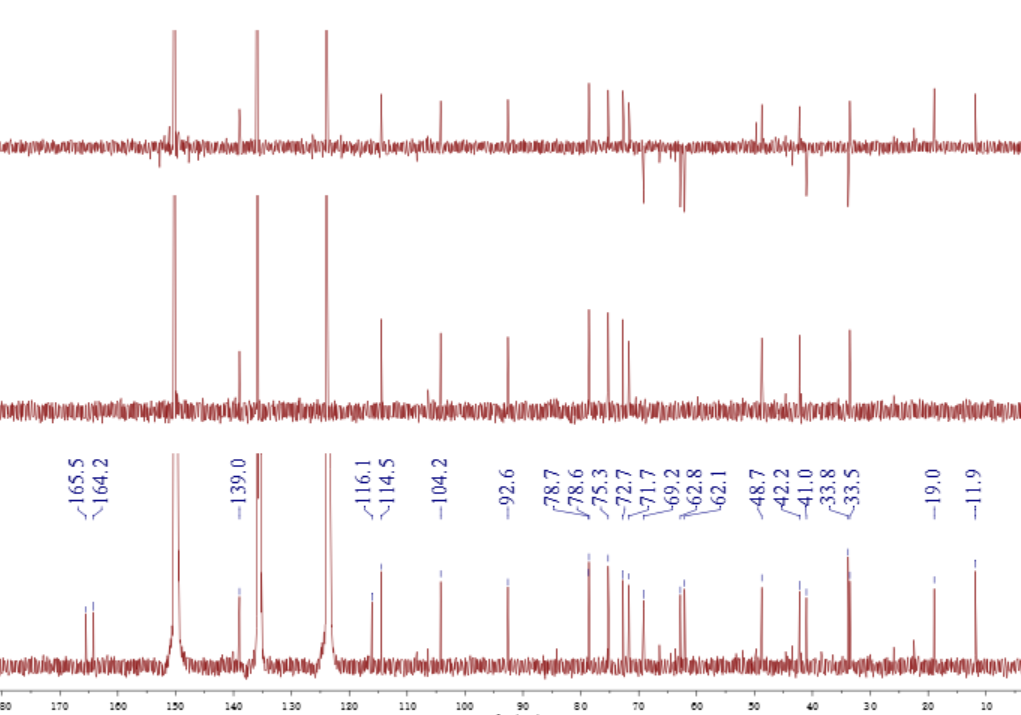


Figure 45 HSQC (Bruker DRX-500, 500 MHz, 125 MHz, C_5_D_5_N) of Patrinoside E (**8**)


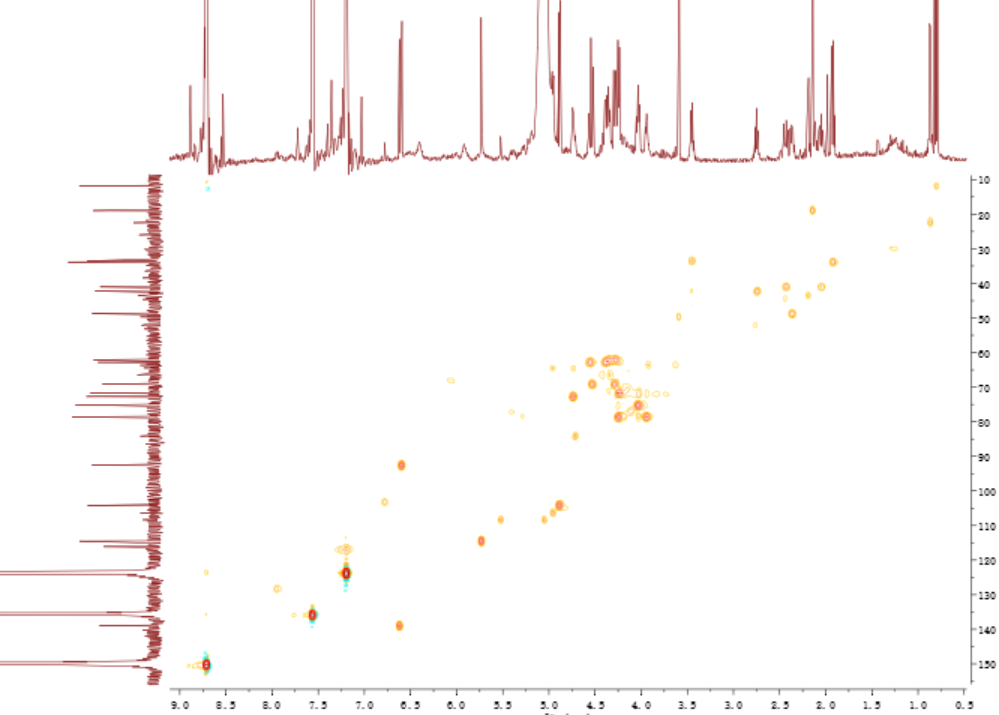


Figure 46 HMBC (Bruker DRX-500, 500 MHz, 125 MHz, C_5_D_5_N) of Patrinoside E (**8**)


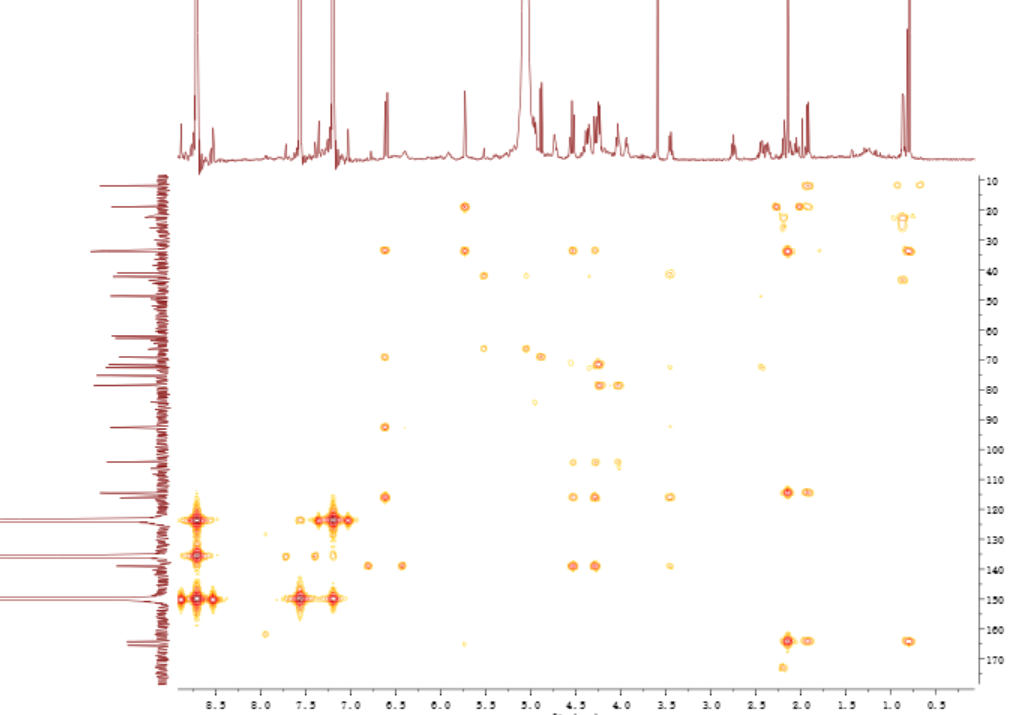


Figure 47 ^1^H-^1^H COSY (Bruker DRX-500, 500 MHz, 500 MHz, C_5_D_5_N) of Patrinoside E (**8**)


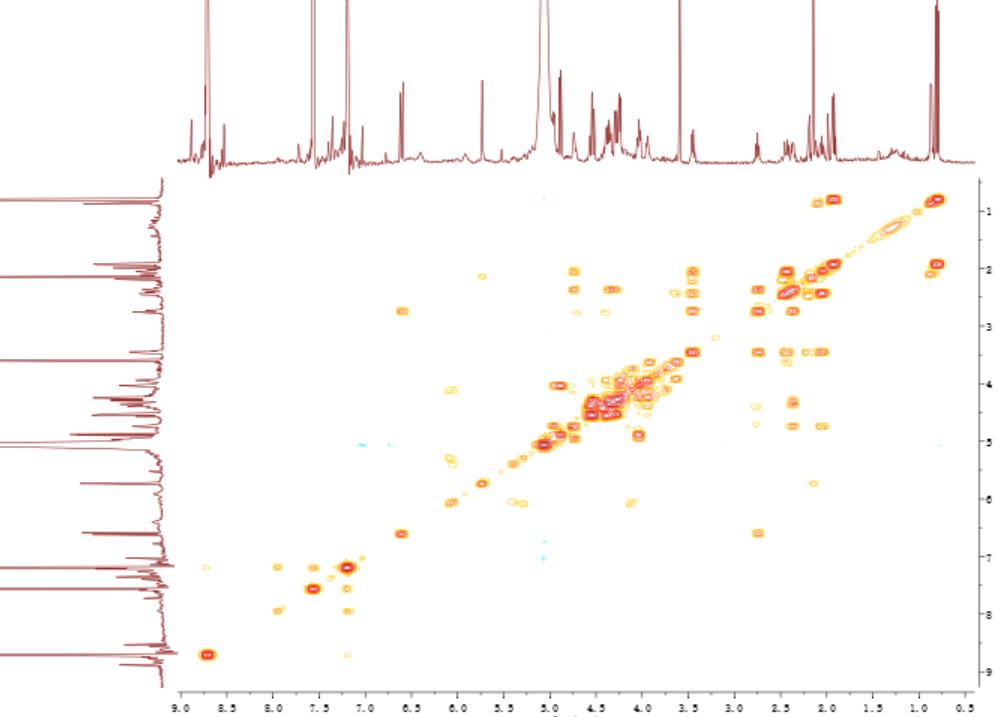


Figure 48 ROESY (Bruker DRX-500, 500 MHz, 500 MHz, C_5_D_5_N) of Patrinoside E (**8**)


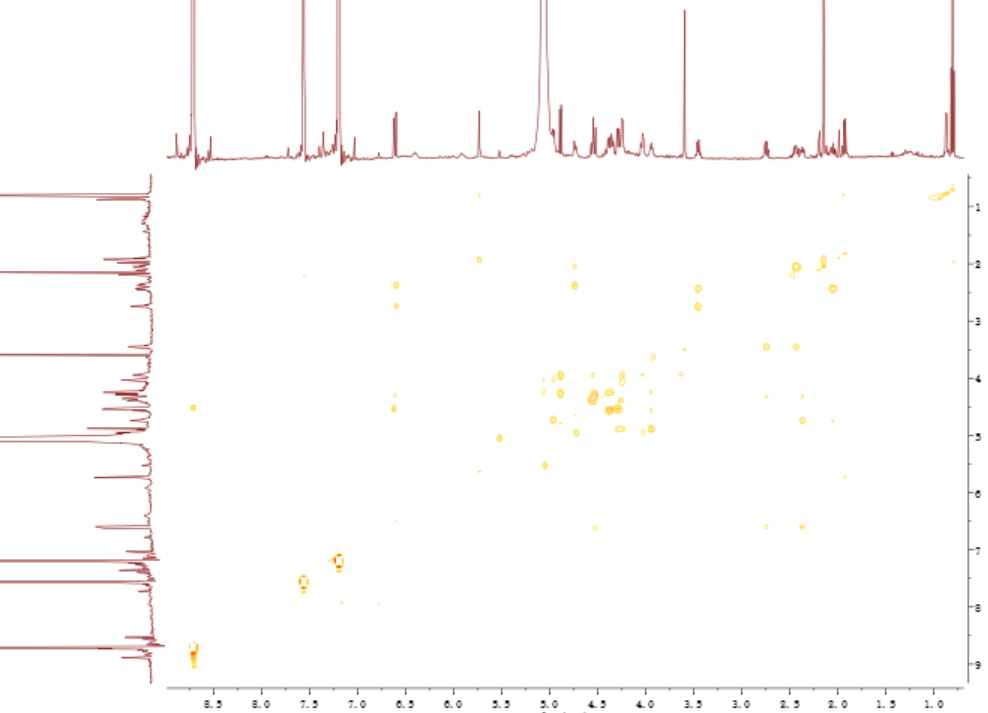


Figure 49 HREIMS spectrum of Patrinoside E (**8**)

Figure 50 ^1^H NMR (Bruker AM-500, 500 MHz, CD_3_OD) of Patrinoside F (**9**)


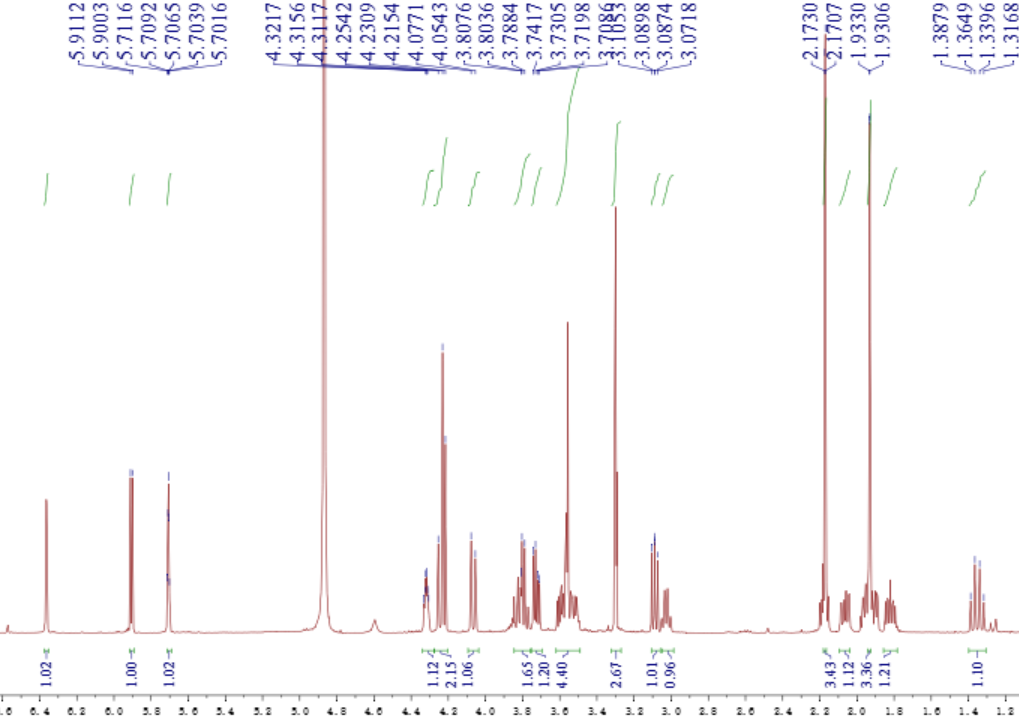


Figure 51 ^13^C NMR (Bruker AM-500, 125 MHz, CD_3_OD) of Patrinoside F (**9**)


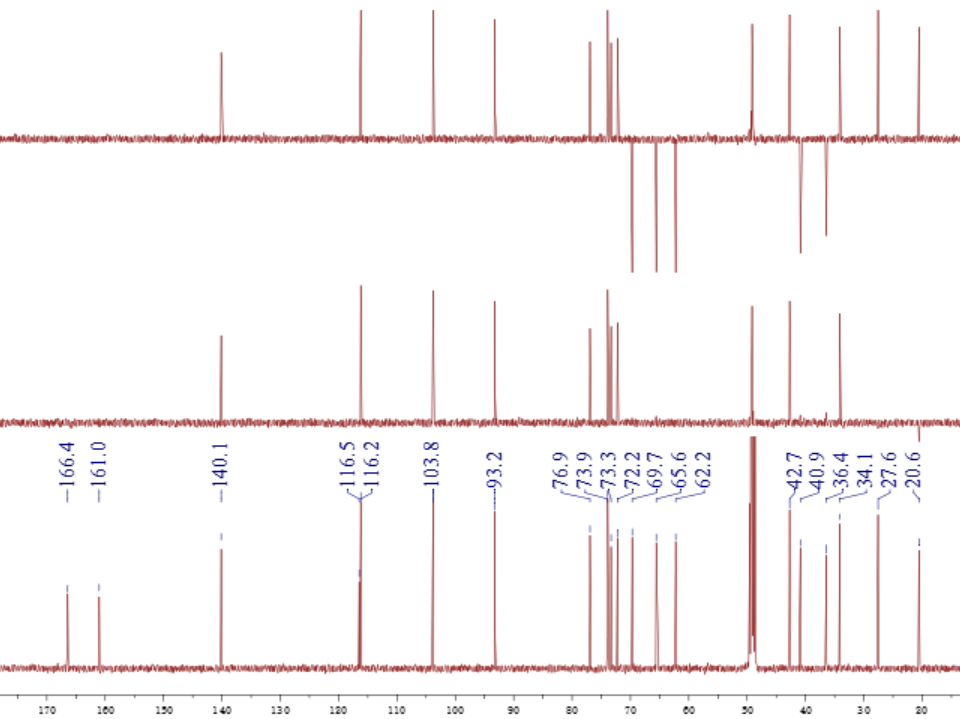


Figure 52 HSQC (Bruker DRX-500, 500 MHz, 125 MHz, CD_3_OD) of Patrinoside F (**9**)


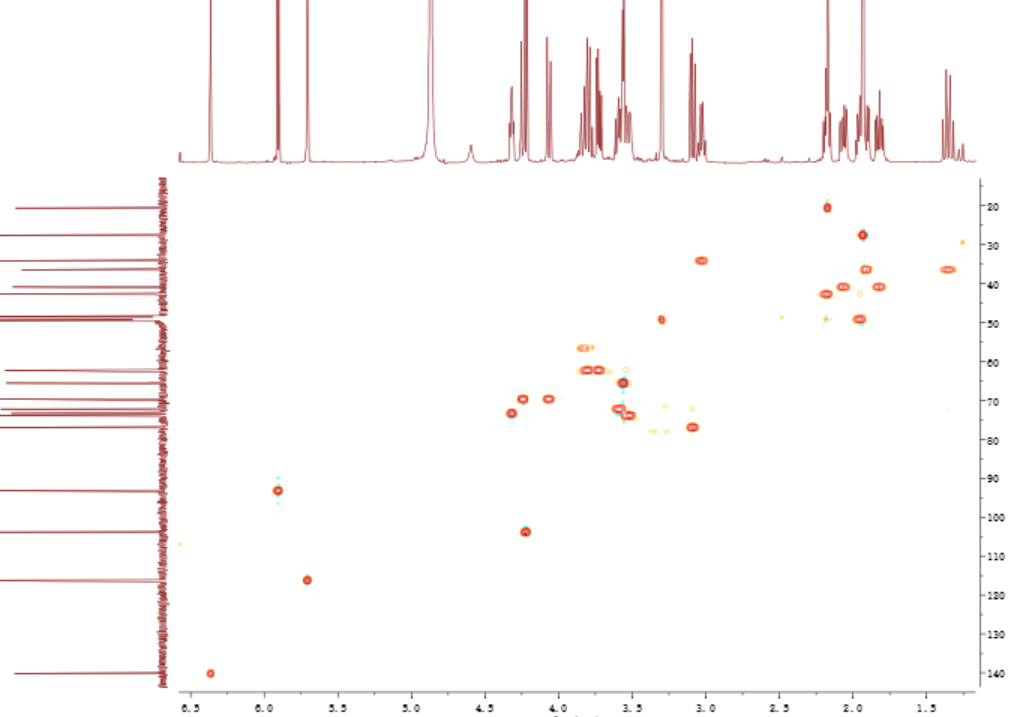


Figure 53 HMBC (Bruker DRX-500, 500 MHz, 125 MHz, CD_3_OD) of Patrinoside F (**9**)


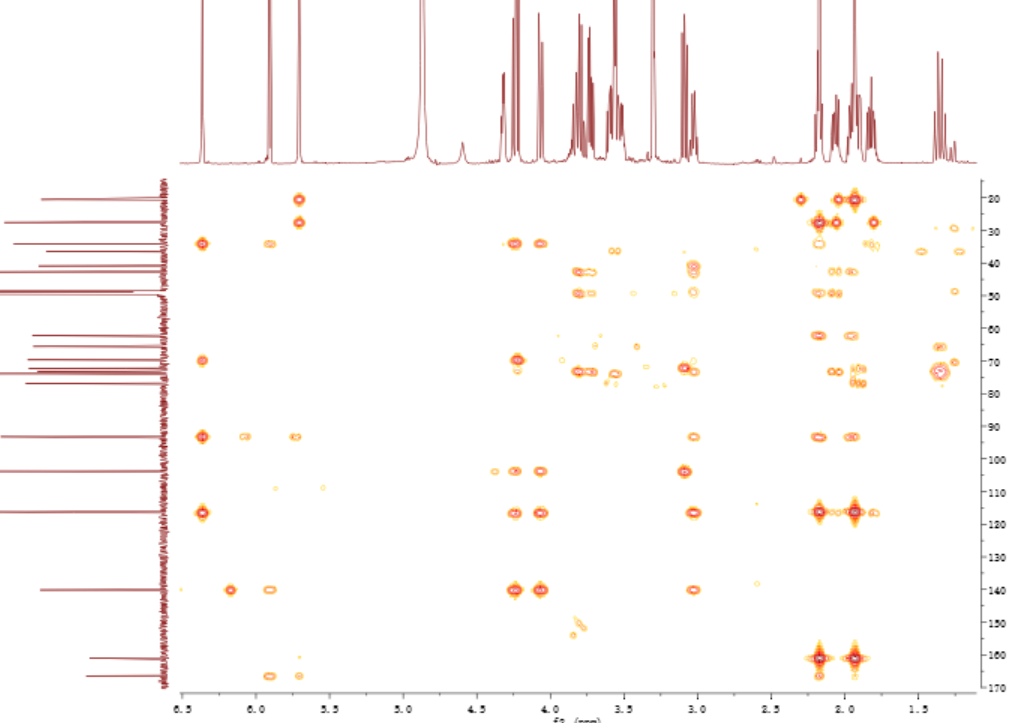


Figure 54 ^1^H-^1^H COSY (Bruker DRX-500, 500 MHz, 500 MHz, CD_3_OD) of Patrinoside F (**9**)


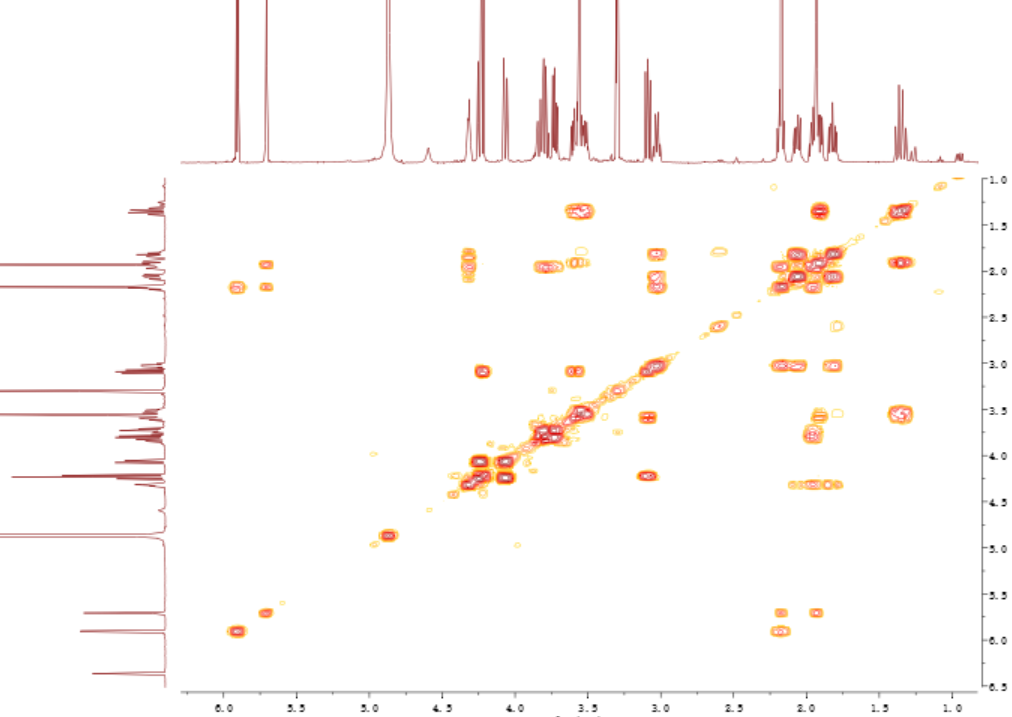


Figure 55 ROESY (Bruker DRX-500, 500 MHz, 500 MHz, CD_3_OD) of Patrinoside F (**9**)


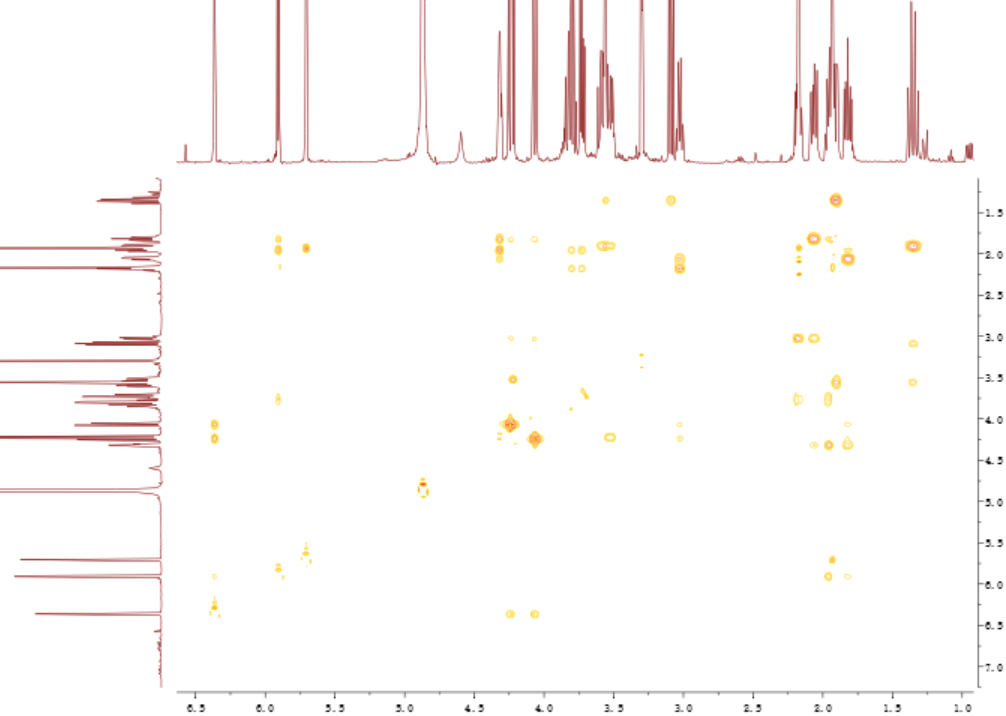


Figure 56 HREIMS spectrum of Patrinoside F (**9**)

Figure 57^1^H NMR (Bruker AM-500, 500 MHz, CD_3_OD) of Patriscabiobisin D (**10**)


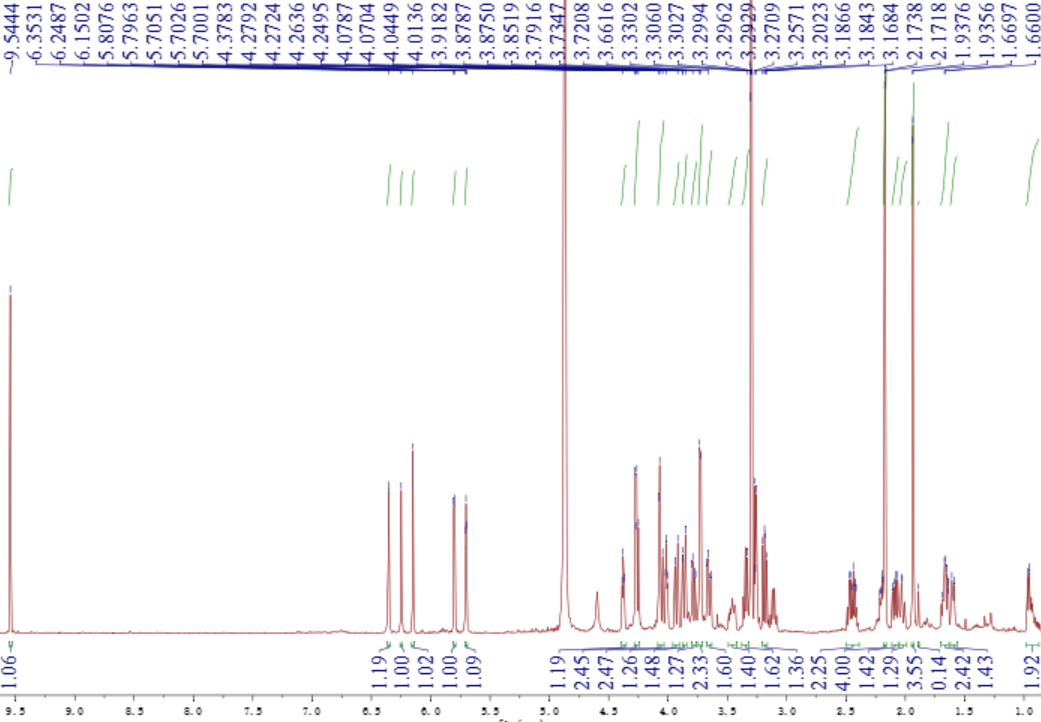


Figure 58 ^13^C NMR (Bruker AM-500, 125 MHz, CD_3_OD) of Patriscabiobisin D (**10**)


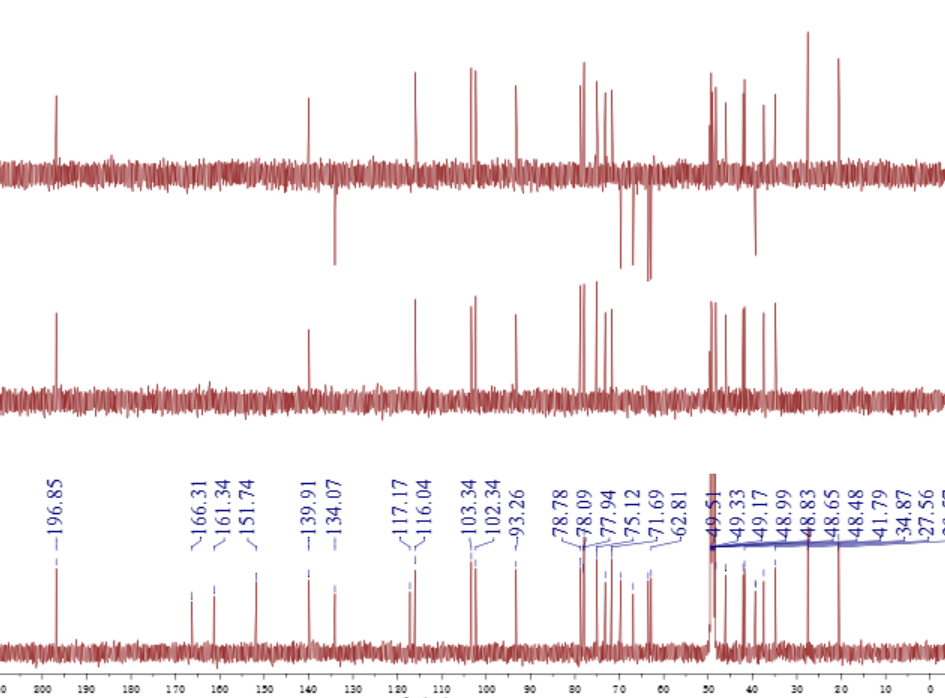


Figure 59 HSQC (Bruker DRX-500, 500 MHz, 125 MHz, CD_3_OD) of Patriscabiobisin D (**10**)


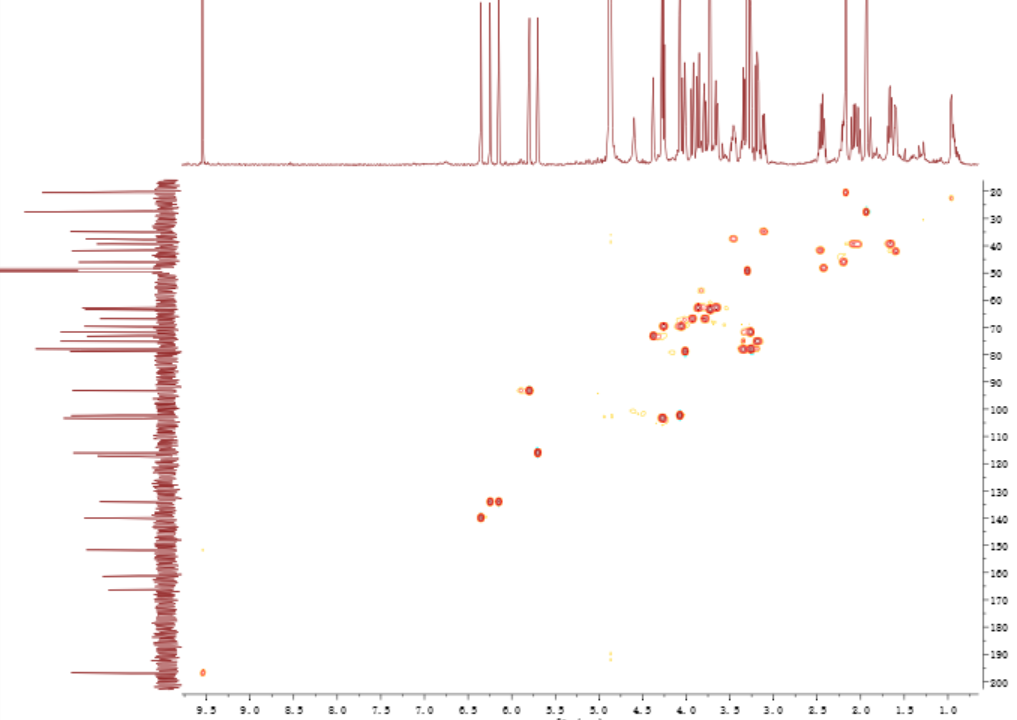


Figure 60 HMBC (Bruker DRX-500, 500 MHz, 125 MHz, CD_3_OD) of Patriscabiobisin D (**10**)


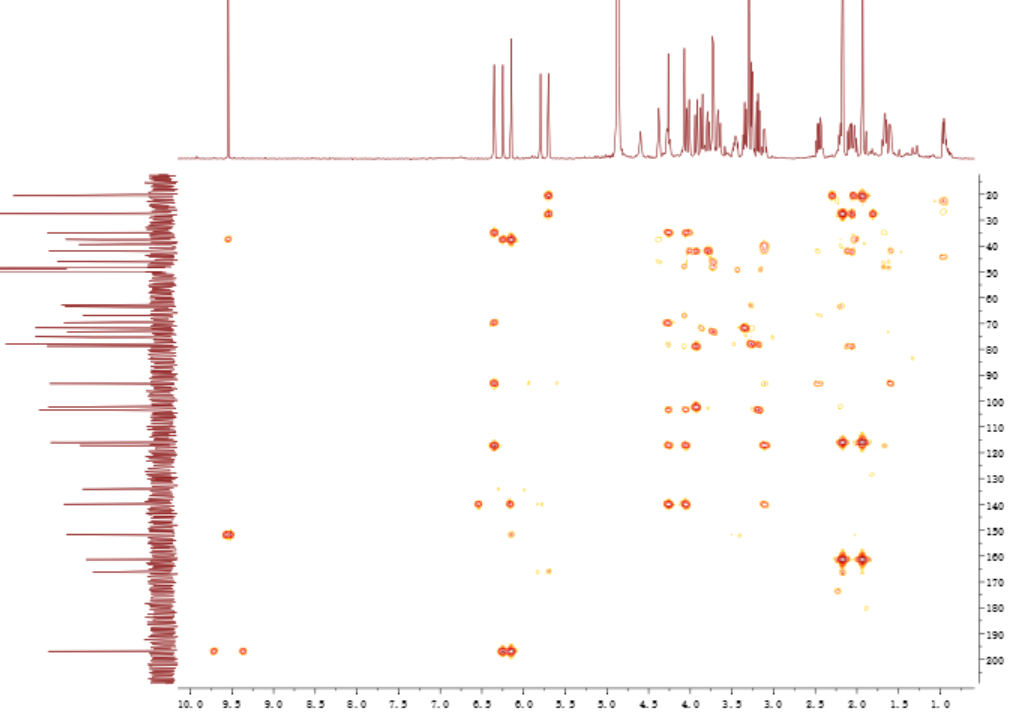


Figure 61 ^1^H-^1^H COSY (Bruker DRX-500, 500 MHz, 500 MHz, CD_3_OD) of Patriscabiobisin D (**10**)


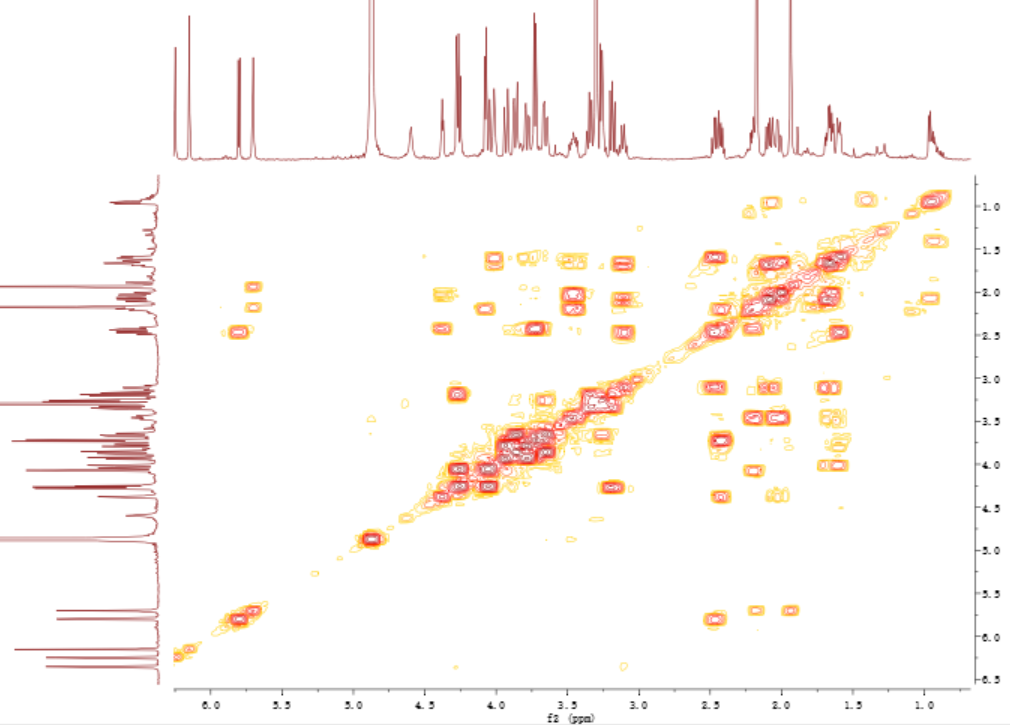


Figure 62 ROESY (Bruker DRX-500, 500 MHz, 500 MHz, CD_3_OD) of Patriscabiobisin D (**10**)


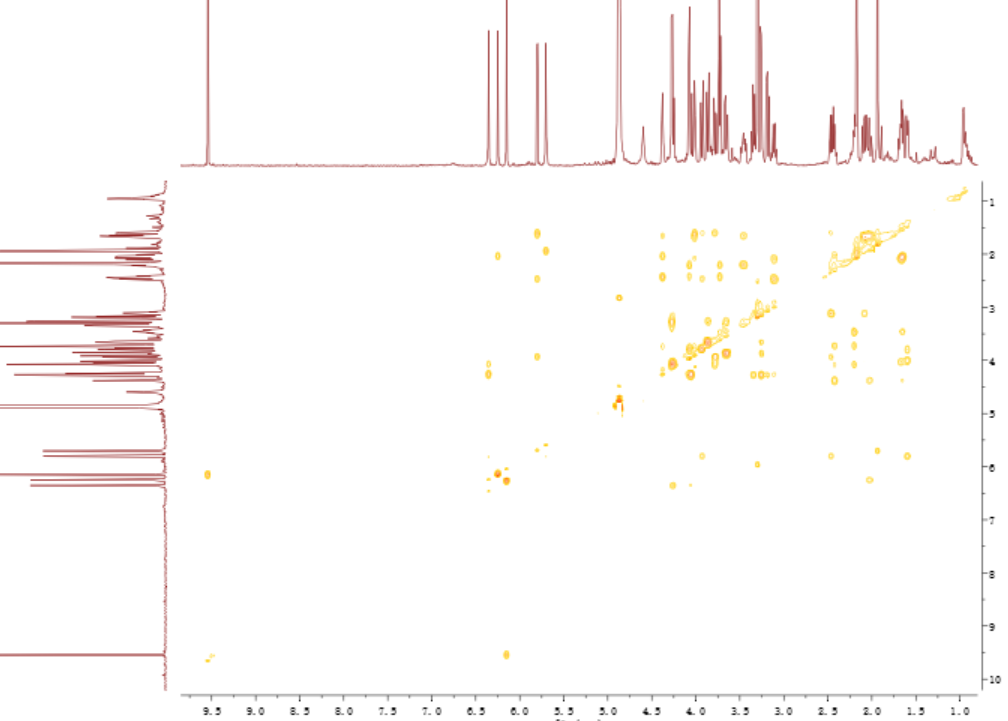


Figure 63 HREIMS spectrum of Patriscabiobisin D (**10**)
